# Dihydropyrazole-Carbohydrazide Derivatives with Dual Activity as Antioxidant and Anti-Proliferative Drugs on Breast Cancer Targeting the HDAC6

**DOI:** 10.3390/ph15060690

**Published:** 2022-05-31

**Authors:** Irving Balbuena-Rebolledo, Astrid M. Rivera-Antonio, Yudibeth Sixto-López, José Correa-Basurto, Martha C. Rosales-Hernández, Jessica Elena Mendieta-Wejebe, Francisco J. Martínez-Martínez, Ivonne María Olivares-Corichi, José Rubén García-Sánchez, Juan Alberto Guevara-Salazar, Martiniano Bello, Itzia I. Padilla-Martínez

**Affiliations:** 1 Laboratorio de Química Supramolecular y Nanociencias, Unidad Profesional Interdisciplinaria de Biotecnología, Instituto Politécnico Nacional, Avenida Acueducto s/n, Barrio la Laguna Ticomán, Ciudad de México 07340, Mexico; irving.balbu@gmail.com (I.B.-R.); astridmriveraa@gmail.com (A.M.R.-A.); 2 Laboratorio de Biofísica y Biocatálisis, Sección de Estudios de Posgrado e Investigación, Escuela Superior de Medicina, Instituto Politécnico Nacional, Plan de San Luis y Salvador Díaz Mirón s/n, Casco de Santo Tomas, Ciudad de México 11340, Mexico; marcrh2002@yahoo.com.mx (M.C.R.-H.); jesmenwej@yahoo.com (J.E.M.-W.); 3 Laboratorio de Diseño y Desarrollo de Nuevos Fármacos e Innovación Biotecnológica, Escuela Superior de Medicina, Instituto Politécnico Nacional, Plan de San Luis y Díaz Mirón, s/n, Col. Casco de Santo Tomas, Ciudad de México 11340, Mexico; syudibeth@hotmail.com (Y.S.-L.); mbellor@ipn.mx (M.B.); 4 Departamento de Química Farmacéutica y Orgánica, Facultad de Farmacia, Universidad de Granada, Campus de Cartuja, 18071 Granada, Spain; 5 Facultad de Ciencias Químicas, Universidad de Colima, Km. 9 Carretera Colima-Coquimatlán, C.P. Coquimatlán, Colima 28400, Mexico; fjmartin@ucol.mx; 6 Laboratorio de Oncología Molecular y Estrés Oxidativo de la Escuela Superior de Medicina, Instituto Politécnico Nacional, Plan de San Luis y Díaz Mirón, s/n, Col. Casco de Santo Tomas, Ciudad de México 11340, Mexico; iolivares@ipn.mx (I.M.O.-C.); jrgarcias@ipn.mx (J.R.G.-S.); 7 Departamento de Farmacología, Escuela Superior de Medicina, Instituto Politécnico Nacional, Plan de San Luis y Díaz Mirón, S/N, Ciudad de México 11340, Mexico; jguevaras@ipn.mx

**Keywords:** HDAC6, breast cancer, TNBC, 4,5-dihydropyrazole, pyrazoline, antioxidant

## Abstract

Breast cancer (BC) is the most frequently diagnosed cancer and is the second-most common cause of death in women worldwide. Because of this, the search for new drugs and targeted therapy to treat BC is an urgent and global need. Histone deacetylase 6 (HDAC6) is a promising anti-BC drug target associated with its development and progression. In the present work, the design and synthesis of a new family of dihydropyrazole-carbohydrazide derivatives (DPCH) derivatives focused on HDAC6 inhibitory activity is presented. Computational chemistry approaches were employed to rationalize the design and evaluate their physicochemical and toxic-biological properties. The new family of nine DPCH was synthesized and characterized. Compounds exhibited optimal physicochemical and toxicobiological properties for potential application as drugs to be used in humans. The in silico studies showed that compounds with –Br, –Cl, and –OH substituents had good affinity with the catalytic domain 2 of HDAC6 like the reference compounds. Nine DPCH derivatives were assayed on MCF-7 and MDA-MB-231 BC cell lines, showing antiproliferative activity with IC_50_ at μM range. Compound **2b** showed, in vitro, an IC_50_ value of 12 ± 3 µM on human HDAC6. The antioxidant activity of DPCH derivatives showed that all the compounds exhibit antioxidant activity similar to that of ascorbic acid. In conclusion, the DPCH derivatives are promising drugs with therapeutic potential for the epigenetic treatment of BC, with low cytotoxicity towards healthy cells and important antioxidant activity.

## 1. Introduction

According to the World Health Organization, breast cancer (BC) is the leading cause of women’s death, with greater than two million new cases and more than six hundred thousand deaths per year worldwide [1,2]. BC is considered a heterogeneous disease and can be classified as: luminal A, luminal B, or triple-negative breast cancer (TNBC). Luminal A is characterized by the presence of estrogenic receptor (ER+), presence or absence of progesterone receptor (PR±), and absence of epidermal growth factor receptor-2 (HER2-); luminal B is characterized by (ER+), (PR±), and (HER2+); whereas the TNBC lacks expression of the three receptors (ER-), (PR-) and (HER2-) [3,4]. Several BC subtypes are treated with different primary therapeutic protocols, but none of them use epigenetic drugs [5]. Interest in treating BC with epigenetic drugs is increasing, mostly because epigenetic alterations such as DNA methylation and acetylation status of histones have been identified as important factors that contribute to tumorigenesis and BC progression [6,7]. Currently, there are no efficient, targeted treatment options for TNBC. Therefore, the identification of new drug candidates for this treatment is an emergent field of research.

Protein acetylation balance is regulated by histone acetyltransferases (HAT) and deacetylases (HDACs); these enzymes play an essential role in post-translational modifications [8,9]. HDACs are a family of hydrolases that remove acetyl groups of lysine residues from histones [10,11] and regulate the expression of tumor suppressor genes, cell cycle progression, and epigenetic transcription [12,13,14]. Cancer, autoimmune, and psychiatric diseases are some human diseases associated with HDACs malfunctioning [15,16,17]. There are eighteen HDAC isoforms identified in mammals, classified into four classes: class I (HDAC 1, 2, 3, and 8) [18]; class II, which is subdivided into IIa (HDAC 4, 5, 7, and 9) and IIb (HDAC 6 and 10); class III, which are also called sirtuins (HDAC 12-18); and class IV (HDAC11) [19]. Classes I, II, and IV are Zn^2+^-dependent, while class III are NAD^+^-dependent [20,21,22]. HDAC6 is primarily expressed in the cytoplasm and encodes a protein of 1215 amino acid residues, the most essential protein of the HDACs family [23]. HDAC6 has a particular structure; it is the only HDAC that contains an internal dimer of two functional catalytic domains, which are named DD1 (G87-G404) and DD2 (G482-G800). They are located at the *N*-terminal and the central region of the protein-bound by the linker region (D405-T481), where dynein motor binding (DMB) domain is found (V439-V503). HDAC6 maintains the acetylation balance of a wide variety of cytoplasmic proteins [19,24,25,26,27], and its overexpression has been associated with various leading diseases such as cancer [28], neurodegenerative diseases, and pathological autoimmune response. For these reasons, selective HDAC6 inhibitors have been extensively investigated to treat these diseases [29,30,31,32,33,34]. Even when HDAC6 possesses two catalytic domains, its activity can only be attributed to DD2. Indeed, entire HDAC6 inhibitors have been developed to date target this domain, including the selective inhibitor tubacin [31,35,36].

On the other hand, oxidative stress leads to several chronic degenerative diseases and disorders, including cancer [37]. BC etiology is multifactorial; moreover, it has been clearly linked to oxidative stress as an essential risk factor [38]. The oxidative stress induced by reactive oxygen species (ROS) is considered as a dynamic imbalance between endogenous levels of antioxidants and the amount of antioxidants lost to ROS scavenging and protects against their harmful effects [39]. In this context, some studies support that antioxidant supplements may reduce the risk of BC recurrence or BC-related mortality [40,41] more than exerting a protective effect [42]. Within tumor cells, increased ROS levels create an inflammatory environment conducive to tumor progression and dissemination to distant organs [43]. Therefore, attenuation of oxidative stress with an antioxidant should result in reduced size and likelihood of metastasis. In this sense, several efforts have been made to combine anti-inflammatory [44] and antioxidant [45,46] effects in the same molecule or in mixtures [47] for potential anticancer treatments.

**Figure 1:**
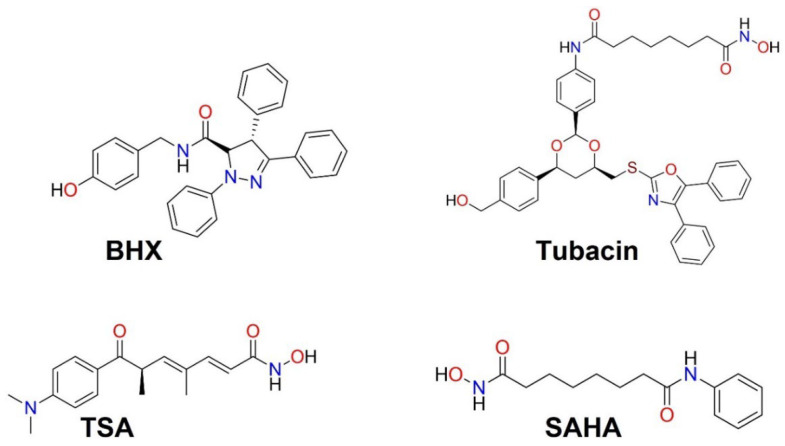
BHX, tubacin, trichostatin A (TSA), and suberoylanilide hydroxamic acid (SAHA).

Therefore, attention to the development of novel and effective anticancer agents with more selectivity and fewer associated side effects, is required for the disease’s eradication. In this context, 4,5-dihydropyrazole derivatives have attracted attention due to their biological activities, such as anti-inflammatory, antidepressant, and potent antiproliferative activity, specifically against BC [48,49,50]. Particularly relevant is *N*-(4-hydroxybenzyl)-1,3,4-triphenyl-4,5-dihydro-1*H*-pyrazole-5-carboxamide (BHX), Figure 1, whose activity against cancer as a Wnt/β-catenin-signaling inhibitor has been demonstrated [51].

In this work, a set of nine dihydropyrazole-carbohydrazide derivatives (DPCH) with potential inhibitory activity on the DD2-HDAC6 domain were modelled in silico. They were synthesized and evaluated in their physicochemical and toxicobiological properties. Moreover, docking simulations on the DD2-HDAC6 domain were performed in order to obtain the non-bonding interactions and the binding free energy values (ΔG°). The results were compared with tubacin, trichostatin A (TSA), and suberoylanilide hydroxamic acid (SAHA) as reference compounds, Figure 1. Antiproliferative assays on BC cell lines MCF-7 and MDA-MB-231 and the nonmalignant cells 3T3/NIH (fibroblast cells) and MCF10A (breast epithelial cells) were performed. MCF-7 and MDA-MB-231 cell lines were considered as models of the most common and the most aggressive subtypes of BC, respectively. Additionally, the overexpression of HDAC6 in both BC cell lines has been demonstrated [28,52]. MCF-7 is the A luminal type, ER and PR (+); MDA-MB-231 is the C-type (claudin-low) and triple-negative—ER, PR and HER2 (−)—also known as triple negative BC (TNBC). Finally, the in vitro inhibition of HDAC6 enzyme as well as its antioxidant properties was demonstrated. The results were supported by quantitative structure–activity relationship analysis (QSAR).

## 2. Results and Discussion

### 2.1. Design Features of Compounds ***2a***–***i***

**Figure 2:**
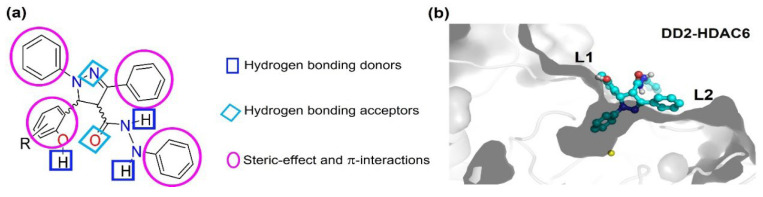
(**a**) Steric effects and noncovalent interactions considered as structural design elements of compounds **2a**–**i**. (**b**) The expected method for compounds **2a**–**i** to cap the entrance of the active site cleft through noncovalent binding interactions in the shallow L1 and L2 pockets.

Several studies have suggested that the catalytic tunnel of DD2-HDAC6 is wider and shallower than other isoforms [53]. Thus, the inclusion of large, bulky aromatic rings in the designed molecules could be useful in fitting into the cap region of the enzyme, more specifically into pockets L1 and L2 [54]. In fact, the aromatic rings are useful for increasing affinity and selectivity by DD2-HDAC6 over the other isoforms, which are mediated by noncovalent interactions [53,55,56]. On the other hand, the hydroxamic acid is the most extended chelating zinc group used in HDACs inhibitors, even though this group is susceptible to be degraded and it is not stable in the organism [57,58]. Additionally, several researchers have focused on replacing the hydroxamic group for other functionalities [54]. In this context, we provide a set of novel compounds based on 4,5-dihydropyrazole heterocycle with a pending 4-carbohydrazide group and bearing several aromatic rings, Figure 2. Despite the bulky effect contribution, they are expected to bind to the cap region of DD2-HDAC6 domain through hydrogen bonding and π-interactions.

### 2.2. Synthesis of DPCH Derivatives

**Scheme 1:**
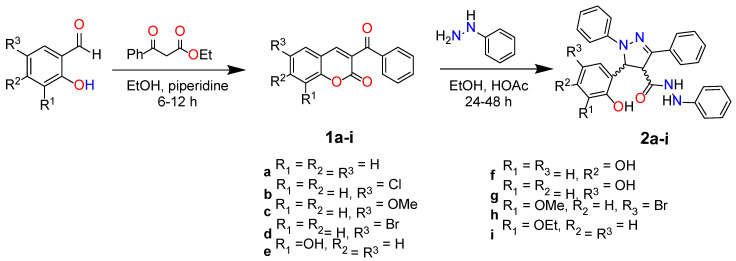
Synthesis of DPCH derivatives **2a**–**i**.

The synthesis of DPCH derivatives (**2a**–**i**) is depicted in Scheme 1. This approach includes two reactions. Firstly, a Knoevenagel condensation between the substituted salicylaldehyde and ethyl 3-oxo-3-phenylpropanoate was achieved to yield the corresponding 3-benzoyl-2*H*-1-benzopyran-2-one **1a**–**i**. In the second step, compounds **1a**–**i** were treated with phenylhydrazine and glacial acetic acid as a catalyst under reflux of EtOH to obtain the corresponding DPCH derivatives **2a**–**i** with poor-to-good yields (20–60%) as a racemic mixture. The compounds precipitated, leaving in solution the more soluble 1,3-diphenylchromene[4,3-c]pyrazole-4(1*H*)-ones and their corresponding phenylhydrazones [59]. It is worth highlighting that the final products required no further purification (purity > 98%). At this point, a brief comparison with BHX synthesis, a closely related compound to **2a**–**i**, seems appropriate, Figure 1. The synthesis of BHX is attained after four steps, with yields after chromatography of 98.9, 10.4, 72.0, and 56.5%, respectively, to give a final 4.2% overall yield [51]. This yield highlights the benefits of the method herein reported.

One of the striking structural features of compounds **2a**–**i** is the cis disposition between H4 and H5. This stereochemistry was suggested by the coupling constant value between these hydrogen atoms (*^3^J*), which is around 12 Hz. A nuclear Overhauser effect (nOe) experiment was performed for the assignment of the ^1^H signals belonging to compound **2a**, Figure S1. The selected signals were H4, H5, and H21, which are at lower frequencies in the spectrum and separated from each other. In a nOe spectra, all signals are vanished except those corresponding to the hydrogen atoms that are coupled or close in space to the irradiated signal. Then, nOe on H4 (d, δ 4.95) allowed assignment of the amide proton (δ 9.79) and H11 (δ 7.81) and irradiation on H5 allowed assignment of H7 (δ 6.92), whereas the absence of nOe on H19 indicates that the phenolic ring is out of the plane of the pyrazole ring and opposite to H5. Finally, the nOe on H21 (δ 6.03) allowed the NHPh to be assigned at δ 7.56. Once the signals for the *ortho* protons of the three monosubstituted rings had been identified, the other signals were assigned with homo- and heteronuclear two-dimensional spectra.

**Scheme 2:**
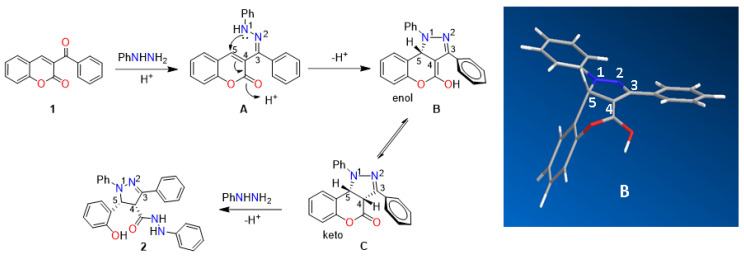
Mechanistic proposal for the conversion of **1** to **2**. The structure of the enol intermediate **B** is shown in the right panel.

The reaction proceeded through the intramolecular 1,4-addition of N1H to the α,β-unsaturated lactone carbonyl of the 3-benzoyl-coumarin-phenyl hydrazone **A** to form the pyrazole ring. The pyranol ring in **B** adopts a boatlike conformation with cyclic oxygen and C5 atoms positioned on the vertexes out of the plane of the boat conformer. The enol form **B** is then tautomerized to the keto form **C** to give the cis isomer. The stereoselectivity of this reaction is explained because of the steric effect exerted by both the coumarin benzofused ring and the C3-Ph ring that limit the approaching of H^+^ to the opposite face occupied by H5, leading to the formation of the cis isomer as a single diastereoisomer. The final product **2** is achieved as a racemic mixture after the amidation and ring opening of the pyrone ring of the intermediate **C** by a second phenylhydrazine molecule, Scheme 2.

### 2.3. Molecular Structure of Compound ***2a***

**Figure 3:**
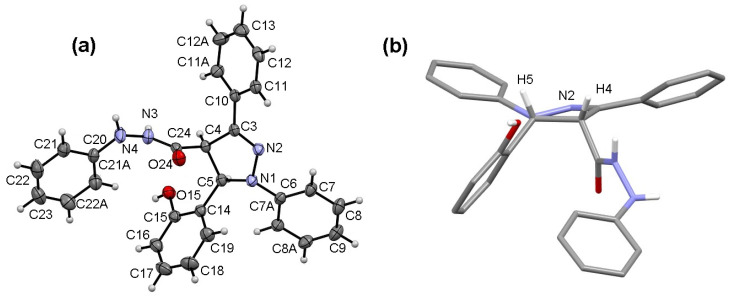
(**a**) Ortep plot at 50% probability level of the DMSO (not shown) solvate of compound **2a**-(4*R*, 5*R*) enantiomer. (**b**) Stick plot of compound **2a**—view through the 4,5-dihydro-pyrazole ring—to appreciate the steric effect of the four phenyl rings and the cis disposition between H4 and H5.

The structure of compound **2a** was confirmed by single-crystal X-ray diffraction; it crystallized in the monoclinic crystal system and *P21/c* space group as the DMSO solvate. The molecular structure is shown in Figure 3; the bond lengths, bond angles, and torsion angles are listed in Table S1. The DPCH ring adopts an envelope conformation with C5 positioned in the vertex, as is revealed by the torsion angles’ values: C4—C3—N2—N1, -0.7(3)°; C3—C4—C5—N1, 14.20(19)°; and N2—N1—C5—C4, -12.7(2)°. This conformation is the most frequently observed in the 22 hits of dihydro-pyrazole scaffold retrieved from the CCDC [60]. The torsion angle of C24—N3—N4—C20 of 113.6(3)°, in the phenyl-hydrazone fragment, is closer than that observed in a similar compound (146.3(3)°) (CCDC-188945) [61]. Additionally, the cis disposition between H4 and H5 is confirmed by C24—C4—C5—C14’s torsion angle value of −21.0(3)°. The supramolecular architecture is given by the following hydrogen bonding interactions (D—H∙∙∙A): N3—H3∙∙∙O24, N4—H4···O25 (DMSO), O15—H15∙∙∙O25 (DMSO), and C4—H4A∙∙∙O24. The geometric parameters of these interactions are listed in Table S2.

### 2.4. In Silico Studies

#### 2.4.1. Docking Simulation

The affinity of DD2-HDAC6 enzyme towards **2a**–**i** was theoretically studied using molecular docking simulations. Docking studies allowed us to obtain the free energy of ligand–receptor binding (ΔG_b_°) as well as the dissociative equilibrium constant K_d_ of the nine 2a–i-DD2-HDAC6 complexes and reference compounds (tubacin, TSA, and SAHA). Analysis of the two enantiomers (4S, 5S) and (4R, 5R) of **2a**–**i** present in the racemic mixture of reaction was performed to elucidate the effect of the configuration on ΔG_b_°. Molecular docking results are listed in Table S3. According to the docking calculations, all compounds are active towards DD2-HDAC6. ΔG_b_° values are in the −7.78 to −6.85 kcal/mol range, close to the values obtained for the reference compounds—TSA had a value of −8.59 kcal/mol and SAHA had a value of −7.02 kcal/mol—but 3.1–2.2 kcal/mol smaller than the ΔG_b_° value of tubacin of −9.97 kcal/mol. Moreover, the ΔG_b_° difference between the (4S, 5S) and the (4R, 5R) enantiomers is small—from 0.61 to −0.01 kcal/mol—and in most cases is in favor of the first enantiomer. Therefore, further in silico calculations were performed only on the (4S, 5S) enantiomer.

**Figure 4:**
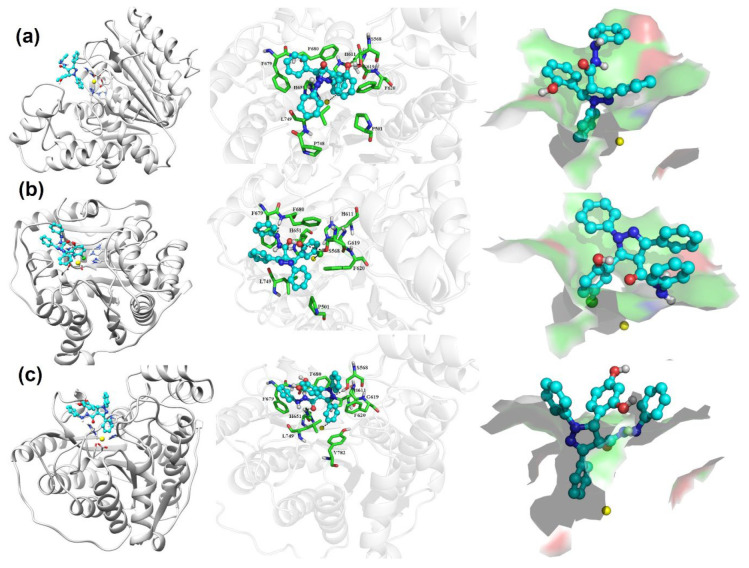
Binding poses at DD2-HDAC6 catalytic domain obtained through blind docking of the target compounds. Compounds: (**a**) **2a**, (**b**) **2b**, and (**c**) **2e**; Zn^2+^ is depicted as yellow sphere. In the left panel, HDAC6 is depicted in white cartoon; in the middle panel, a zoom of the catalytic domain is shown where the AAR are as sticks, and the ligands are shown using a ball and stick representation; in the right panel, a surface representation of the catalytic tunnel is depicted in gray where the insertion of the N-Ph (**a**), C5-PhOH (**b**), and C3-Ph (**c**) rings are appreciated. Figures were built with Pymol and UCSF Chimera software.

The calculated binding modes of **2a**–**i**-DD2-HDAC6 and tubacin-DD2-HDAC6 complexes show that all ligands reached the catalytic binding site of DD2-HDAC6. Compounds **2a**–**i** are anchored on the surface binding domain, and one of the four aromatic rings is slipped into the hydrophobic channel. Those compounds bearing a substituent in the *para* position relative to the phenolic group (**2b**, **2c**, **2g**, and **2h**; not **2d**) seem to favor the positioning of the C5-PhOH ring into the hydrophobic channel over those *ortho*-substituted (**2e** and **2i**), *meta*-substituted (**2f**), or unsubstituted (**2a**), which favor C3-Ph or N-Ph insertion into the hydrophobic channel, respectively, Figure 4. CONHNHPh residue contributes through NH∙∙∙N, NH∙∙∙O, and OH∙∙∙O hydrogen bonding to the ligand anchorage into the rim of the DD2 domain. All ligands interact with S568, F620, F680, H651, F679, and L749 amino acid residues (AAR) of DD2-HDAC6—the same as tubacin, TSA, and SAHA, the compounds used as reference—as well as with H611 (67% of incidence), G619 (72%), D567, T678, and Y782 (33% each), Table S4, through hydrogen bonding, electrostatic, π−π type, and mostly hydrophobic interactions, Figure 4. It is worth mentioning that these interactions are common with other recently reported HDAC6 inhibitors [62,63,64]. As can be seen, compounds **2a**–**i** are locking the entrance to the catalytic tunnel by the 4,5-dihydro-pyrazole moiety, effectively guarding the active site of HDAC6, Figure 4. This binding mode is similar to that shown by tubacin, whose HDAC6 inhibitory activity has been attributed to its bulky and relatively complex capping group [65]. The complete set of binding conformation of complexes between compounds **2a**–**i** and monomeric DD2-HDAC6 is displayed in Figure S2.

#### 2.4.2. Evaluation of Physicochemical and Toxicobiological Properties

The theoretical physicochemical and toxicobiological properties of compounds **2a**–**i**, tubacin, TSA, and SAHA (the triad of compounds used as reference) were analyzed trough Osiris DataWarrior and Osiris Property Explorer software, respectively. The results are listed in Table 1, where it can be seen that most of the tested compounds satisfy Lipinski’s five rules [66]. In general, narrow intervals were observed for MW (448.52–557.44 g mol^−1^), logP (2.2–3.4), number of hydrogen acceptors (HA = 6–7), and hydrogen donors (HD = 3–4), as well as for the number of rotatable bonds (RB = 6–7). Particularly, compounds **2d** and **2h**, substituted with bromine, are out of the range for optimal MW. These theoretical predictions are of high importance for the analysis of hundreds of drugs. Many of them are approved by the U.S. Food and Drug Administration (FDA), even when they violated more than two of Lipinski’s rules [67]. Additionally, the LogS, topological polar surface area (TPSA = 77.0–97.2 Å^2^), absorption percentage by passive diffusion (%ABS = 109 ± 0.345–TPSA), and molar refractivity (MR = 132.1–147.0 cm^3^ mol^−1^) [68] values of each compound were obtained [69]. The whole set of DPCH derivatives is predicted to have good lipid membrane absorption, with %ABS values in the 76–82% range. These values are similar to TSA (85%) and SAHA (82%) and are much better than those for tubacin (51%). Finally, good toxicobiological properties were predicted for **2a**–**i**, Table S5, except for the high risk of being as tumorigenic as TSA. However, it is very common that treatments used for cancer have a critical toxicity profile and cause a number of side effects [70]. Even though substantial progress has been made in antitumor drugs, drug resistance and high toxicity still limit their clinical application [71,72,73].

### 2.5. In Vitro Pharmacological Evaluation

#### 2.5.1. Cell Viability Assays

The antiproliferative activities of **2a**–**i** were evaluated on two types of BC cancer cell lines (MCF-7 and MDA-MB-231). The cytotoxic evaluation of **2a**–**i** was conducted by MTT assay in MCF-7 cells (Figure S3), MDA-MB-231 (Figure S4), the nontumorigenic 3T3/NIH cells lines (Figures S5 and S6), and the nonmalignant breast epithelial cells MCF10A (Figures S7 and S8) and compared with SAHA and TSA as reference drugs. The IC_50_ for SAHA was similar to that reported elsewhere [74]. Results showed that the cytotoxicities of **2a**–**i** are dose-dependent toward both BC cell lines, with IC_50_ values in the µM range, Table 2. The compounds with the best antiproliferative activity in MCF-7 cells were **2c–g** (IC_50_ = 23–28 µM), whereas those with the best activity in MDA-MB-231 cells were **2b** and **2d** (IC_50_ = 24–26 µM), followed by **2c** and **2e–g** (IC_50_ = 32–33 µM). Moreover, the unsubstituted compound **2a** and compounds **2c** and **2i**, substituted with –OR (R = Me, Et) group, were less cytotoxic to normal 3T3/NIH cells (IC_50_ > 100 µM) than the rest of the compounds, particularly those substituted with an –OH group (**2e–g**). Although the tested compounds were not better than TSA and SAHA, they exhibited cytotoxic activities similar to pyrimethamine-hydroxamic acid derivatives towards MCF-7 and MDA-MB-231 cell lines [74]. However, they were better than pyrrolo[2,3-d]pyrimidine-based HDAC inhibitors in MDA-MB-231 cells [75]. In the case of compound **2c**, the IC_50_ value in MDA-MB-231 cells was slightly larger (33 ± 1 μM) than the value of BHX (19.3 μM) but less cytotoxic to nonmalignant MCF-10A (>100μM) than BHX (31.06 μM) [50]. The last comparison allows us to conclude that the cis disposition between the C5-Ph and 4-CONHNHPh groups, in contrast to the trans disposition between C5-Ph and C4-Ph in BHX, could be related with of the lower cytotoxicity of compounds **2c**, **2h,** and **2i** against nontumorigenic cells compared to BHX.

#### 2.5.2. Wound Closure Assays in the MDA-MB-231 BC Line

**Figure 5:**
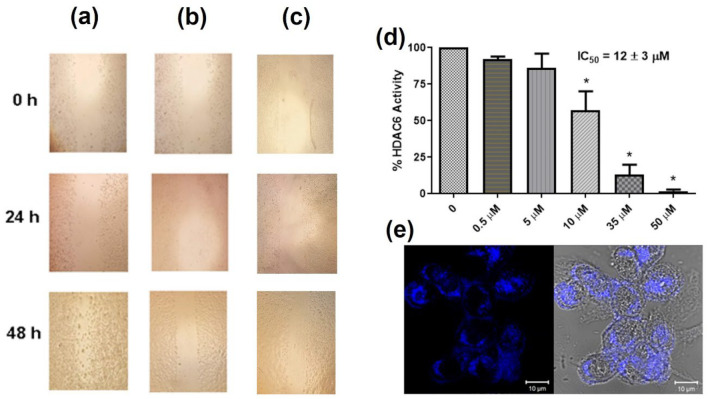
In vitro wound closure cell migration assay with the BC cell line MDA-MB-231 at 0, 24, and 48 h of incubation. (**a**) Control, (**b**) compound **2b,** and (**c**) compound **2c** at 15 µM concentration. (**d**) Dose-dependent curve of HDAC6 inhibition by compound **2b**. Data represent mean ± SEM [* significantly different from AA (*p* < 0.05)]. (**e**) Confocal microscopy images, MCF-7 cells exposed to compound **2b** at a concentration of 10 µM: increased resolution of lasser (left) and low-resolution image (right).

MDA-MB-231 cells are a very aggressive and metastatic cancer line that tends to migrate to other organs. It is known that overexpression of HDAC isoforms (1, 4, 6, and 8) in both MDA-MB-231 and MCF-7 cells is responsible of the invasiveness and migration capabilities of human breast cancer cells [52]. Compounds **2b** and **2c** at ½-IC_50_ concentration (15 µM) were assayed to establish their capability to prevent cell migration. The percent of wound closure was measured after 16, 24, and 48 h of being inflicted. Figure 5a–c shows that the wound closure begins at 24 h, reaching more than 80% after 48 h in the control, whereas treated cells were less than 20% after 48 h of incubation. This result confirms the capability of compounds **2b** and **2c** to prevent the migration of MDA-MB-231 cells.

#### 2.5.3. In Vitro HDAC6 Human Recombinant Inhibition

Compound **2b** was selected to perform the HDAC6 inhibition assay because it showed the best ΔG_b_ of the **2b**-DD2-HDAC6 complex of -7.86 kcal mol^−1^. The assay was validated using TSA as positive control, finding a *Ki* similar to the reported value [76]. Compound **2b** inhibited HDAC6 in a dose-dependent manner at IC_50_ = 12 ± 3 µM, Figure 5d. Although the IC_50_ value is higher than the reported for other HDAC6 inhibitors (nM) [64,74,77], this result could be explained due to the absence of a N-OH group, which is capable of chelating the Zn^2+^ present in the HDAC6 catalytic site. The capability of reaching the cytoplasm, where the HDAC6 is overexpressed [78], was investigated. The intracellular location of compound **2b** was confirmed with confocal laser microscopy taking advantage of the fluorescence exhibited by this compound. The MCF-7 cells were exposed to **2b** at 10 µM for 30 min. Figure 5e is a live cell imaging that shows the entrance of the compound (blue) into the cell. This result agrees with those obtained from the in silico study since, according to the physicochemical properties determined by the Lipinski’s rules, the compounds presented optimal properties for crossing the cell membrane.

### 2.6. QSAR Analysis

**Figure 6:**
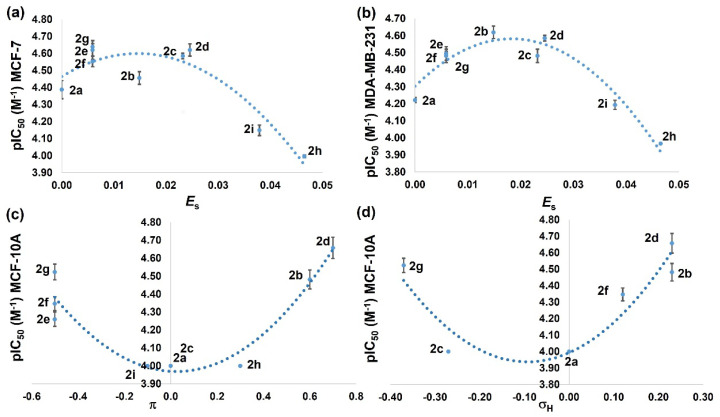
Quantitative structure–activity relationship (QSAR) between the pIC_50_ (M^−1^) and *Es* values of DPCH derivatives on BC cellular lines (**a**) MCF-7 and (**b**) MDA-MB-231. QSAR between the pIC_50_ (M^−1^), (**c**) π, and (**d**) σ_H_ values of DPCH derivatives on healthy cellular line MCF-10A. Polynomial regression of second order through one-way ANOVA test and constant values were analyzed by Student’s *t*-test: (**a**) pIC_50_ = -638.5 ± 1.6 *E_S_*^2^ + 18.6 ± 0.7 *E_S_* + 4.46 ± 0.04 (*n* = 9, *p* < 0.05, *r* = 0.9266); (**b**) pIC_50_ = -831 ± 36 *E_S_*^2^ + 30.4 ± 1.3 *E_S_* + 4.30 ± 0.02 (*n* = 9, *p* < 0.001, *r* = 0.9634); (**c**) pIC_50_ = 1.50 ± 0.15 π^2^–0.076 ± 0.011 + 3.969 ± 0.002 (*n* = 9, *p* < 0.01, *r* = 0.9584); (**d**) 6.4 ± 0.6 σ_H_^2^ + 1.20 ± 0.12 σ_H_ + 3.993 ± 0.002 (*n* = 6, *p* < 0.05, *r* = 0.9186). The *p* < 0.05 and *p* < 0.01 values represent statistically significant differences at 95.0% and 99.0% confidence, respectively.

The correlations found through a QSAR analysis of DPCH derivatives on the most common (MCF-7) and most aggressive (MDA-MB-231) BC cell lines are described below. The pIC_50_ values of the DPCH derivatives show a parabolic correlation with the *E*_s_ descriptor proposed by Taft [79], in the MCF-7 cell line. Therefore, the inhibitory activity on proliferation is dependent on the size of the molecules, the relationship shows that derivatives with MR values between 134 and 139 cm^3^ mol^−1^ have the best activities (**2c**–**g**), while smaller or larger derivatives are significantly less active, Figure 6. Likewise, a similar correlation was found for DPCH derivatives on the MDA-MB-231 cell line with the steric descriptor (*Es*). However, the relationship between the molecular size and antiproliferative activity is more evident; that is, derivatives with medium sizes (**2b**, **2c**, **2d**) showed the best activity in relation to those compounds of smaller (**2a**–**g**) or larger size (**2h**, **2i**), Figure 6, see Figure S9 for 3T3/NIH.

On the other hand, the pIC_50_ values of the DPCH derivatives showed parabolic correlations with the liposolubility descriptor proposed by Hansch (π) [80] and the electronic descriptor proposed by Hammett (σ_H_) [81] on the 3T3/NIH and MCF-10A cell lines. The less toxic derivatives with both cell lines lie at the minimum of the curves. These have π values between −0.1 and 0.3, corresponding to logP values between 2.6 and 3.0 (**2a**, **2c**, **2i** and **2h**), and small, negative values for σ (**2a** and **2c**). It is worth mentioning that in the case of the σ descriptor, only those derivatives with substitutions in the *meta* and *para* positions were considered since σ cannot be appropriately estimated in the *ortho* positions due to the overlap with steric effects.

### 2.7. Antioxidant Activity

**Figure 7:**
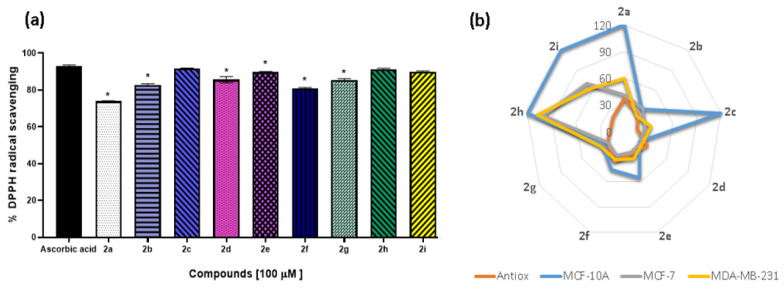
(**a**) DPPH radical scavenging activity of compounds **2a**–**i** vs ascorbic acid (AA) at 100 µM. Data represent mean ± SEM [* significantly different from AA (*p* < 0.05)]. The assays were performed in triplicate. Antioxidant IC_50_ values (μM): 38 ± 3 (**2a**), 26 ± 2 (**2b**), 16 ± 4 (**2c**), 32 ± 3 (**2d**), 26 ± 2 (**2e**), 35 ± 3 (**2f**), 23 ± 3 (**2g**), 17 ± 3 (**2h**), 21 ± 2 (**2i**), and 13 ± 2 (**AA**). (**b**) Comparative graph between the antioxidant and antiproliferative (MCF-10A, MCF-7 and MDA-MB231 cell lines) IC_50_ values (radial axis) of compounds **2a**–**i** (periphery).

The radical scavenging activity (RSA) of **2a**–**i** was assessed by the DPPH test, using ascorbic acid (AA) as control. This assay is widely used to evaluate the antioxidant capabilities of natural and synthetic compounds, where DPPH is the free radical which can accept an electron or hydrogen atom and become reduced [82]. The results are shown in Figure 7a; all compounds exhibited antioxidant activities above 75% at 100 µM, with **2c**, **2e**, **2h**, and **2i** being the most active (up to 90%) and similar to AA (93%). The DPPH RSA IC_50_ values of all compounds are in the 16–38 μM range; the best performances are shown by compounds **2c** (16 ± 4 μM) and **2h** (17 ± 3 μM) with values comparable to AA (13 ± 2 μM, 13.9 μM) [83]. The complete dose–response curves are displayed in Figure S10. It is worth noting that small IC_50_ values are desired for antioxidant and antiproliferative activities against malignant cells (MCF-7 and MDA-MB231) and large IC_50_ values for antiproliferative activity against nonmalignant cells (MCF-10A). This relationship is clearly appreciated for compounds **2c**, **2a**, and **2i** in the spiderweb chart shown in Figure 7b. Therefore, these compounds can be considered as effective dual anticancer–antioxidant agents with reduced cytotoxicity in normal breast cells by decreasing ROS production—characteristics desired for diminishing some undesirable side effects of chemotherapy [47]—and also for BC treatment [84].

## 3. Materials and Methods

### 3.1. Instrumental and Chemicals

All reagents and solvents were purchased from commercial suppliers and used without further purification. Thin-layer chromatography (TLC) developments were performed on silica gel coated (Merck 60 F254) aluminum foils. Yields are reported after final isolated products with 98–100% purity (HPLC-Agilent Technologies 1260 Infinity Series system). Melting points were measured in an Electrothermal IA 91000 devise and are uncorrected. Proton and carbon-13 nuclear magnetic resonance (^1^H and ^13^C NMR) spectra were recorded on a Varian Mercury NMR spectrometer operating at 300 MHz (^1^H, 300.08; ^13^C, 75.46 MHz), using deuterated dimethylsulfoxide (DMSO-*d6*) as a solvent; chemical shift values (δ) are reported in parts per million (ppm), using as reference the residual solvent peaks (^1^H, δ 2.50; ^13^C, δ 39.52) and coupling constants *^n^J*(H–H) are in Hz. Multiplicity of the signals are expressed as: s (singlet), d (doublet), t (triplet), q (quartet), or m (multiplet), Figures S11–S28. The complete assignment of ^1^H and ^13^C-NMR were performed with COSY and HETCOR 2D experiments, Figures S29 and S30, and nOe 1D spectra, Figure S1. The numbering scheme for NMR assignments is shown in Figure 3a. Infrared (IR) spectra were recorded neat in a Perkin-Elmer Spectrum GX series with an FT-IR System Spectrophotometer using the ATR devise, the intensity of the signals was indicated as: weak (w), medium (m), strong (s), or very strong (vs), Figures S31–S39. Mass spectrometry was performed on an Agilent UHPLC-Mass Spectrometer 6545 Q-TOF LC/MS, using acetonitrile as solvent, Figures S40–S53, for purity.

### 3.2. X-ray Structure Determination

General crystallographic data for **2a** has been deposited in the Cambridge Crystallographic Data Centre as supplementary publication number CCDC 2108339. A summary of the collection and refinement of the X-ray data is listed in Table S6. Single crystal X-ray diffraction data were collected on an Oxford Xcalibur Ruby Gemini area detector diffractometer at 293(2) K with Mo Kα radiation (λ = 0.71073 Å). Cell refinement and data reduction were carried out with the CrysAlis RED software [85]. The structures were solved by direct methods using the SHELXS2014 program [86] of the WINGX package [87]. The final refinement was performed by full-matrix least-squares methods using the SHELX2014 program [86]. H atoms on C were positioned geometrically and treated as riding atoms with C−H 0.93−0.98 Å, Uiso(H) = 1.2 eq(C), and H atoms on O or N were found by Fourier difference and freely refined. Platon [88] and Mercury [89] were used to prepare the material for publication.

### 3.3. Chemical Synthesis of Substituted DPCH Derivatives ***2a***–***i***

Compounds **1a**–**i** are known, but they are not commercially available. Therefore, they were synthesized as follows: in a 250 mL ball flask, the corresponding amount of salicylaldehyde was placed together with ethyl benzoyl acetate in 1:1.1 molar ratio in 30 mL of ethyl alcohol as solvent and 3 drops of piperidine as catalyst. It was allowed to stir at reflux for 12 h. The product was filtered under vacuum and washed with ethanol. Their spectroscopic characterization agrees with the literature [90,91,92] (see ESI).

(±)-5-(2′-Hydroxyphenyl)-*N*′,1,3-triphenyl-4,5-dihydro-1*H*-pyrazole-4-carbohydrazide (**2a**). To a solution of 0.300 g (1.20 mmol) of **1a** dissolved in 25 mL of ethanol, 0.35 mL of phenylhydrazine (3.6 mmol), 4.0 mL of distilled water and 4 drops of glacial acetic acid were added. The reaction was allowed to reflux with magnetic stirring for 24 h. After completion, the reaction mixture was allowed to cool at room temperature or until the formation of a white solid was observed. The solid was filtered under vacuum, washed with ethanol (2 × 3 mL) and allowed to dry at room temperature to obtain 0.158 g (0.35 mmol, 30% yield, 98.92% purity) of a white fluorescent solid, mp = 207–210 °C. IR (cm^−1^): 3345 (w), 3253 (w) (N-H, O-H), 1645 (m, C=O), 1594 (m), 1494 (s) 1455 (m) (C=C, Ph), 1365 (m), 1223 (m), 772, 750, 688 (vs, C-H Aromatic out of plane). RMN ^1^H δ: 10.03 (s, 1H, OH), 9.79 (s, 1H, CONH), 7.81 (dd, 2H, ^3^*J* = 8.2, ^4^*J* = 1.8, H11), 7.56 (s, 1H, PhNH), 7.43 (dd, 2H, ^3^*J* = 7.6, ^4^*J* = 8.2, H12), 7.37 (dd, 1H, ^3^*J* = 7.6, ^4^*J* = 1.8, H13), 7.14 (dd, 3H, ^3^*J* = 7.3, ^3^*J* = 8.2, H8), 7.14 (t, ^3^*J* = 7.6, H17), 7.00 (d, 2H, ^3^*J* = 7.6, H16, H19), 6.92 (d, 2H, ^3^*J* = 8.2, H7), 6.86 (dd, 2H, ^3^*J* = 8.2, ^3^*J* = 7.6, H22), 6.77 (t, 1H, ^3^*J* = 7.3, H9), 6.57 (t, 1H, ^3^*J* = 7.6, H18), 6.53 (t, 1H, ^3^*J* = 7.6, H23), 6.03 (d, 2H, ^3^*J* = 8.2, H21), 5.62 (d, 1H, ^3^*J* = 12, H5), 4.95 (d, 1H, ^3^*J* = 12, H4). RMN ^13^C δ: 167.6 (CO), 154.9 (C15), 148.9 (C3), 147.7 (C20), 145.8 (C6), 132.4 (C10), 129.8 (C19), 129.2 (C13), 129.1 (C12), 129.0 (C8), 128.9 (C17), 128.8 (C22), 126.2 (C11), 122.2 (C14), 120.2 (C9), 119.4 (C18), 118.4 (C23), 115.3 (C16), 115.0 (C7), 112.2 (C21), 63.1 (C5), 54.7 (C4). Mass analysis [M-H]^+^ (m/z): 449.1978 found, 449.1978 calculated.

(±)-5-(5′-Chloro-2′-hydroxyphenyl)-*N*′,1,3-triphenyl-4,5-dihydro-1*H*-pyrazole-4-carbohydrazide (**2b**). Synthesized as described for **2a** starting from 0.300 g (1.05 mmol) of **1b**, 0.35 mL of phenylhydrazine (3.6 mmol) to obtain 0.170 g (0.35 mmol, 33% yield, 97.88% purity) of a white fluorescent solid, mp = 205–207 ºC. IR (cm^−1^): 3330 (w), 3257 (br) (N-H, O-H), 1645 (m, C=O), 1595 (m), 1494 (s) 1419 (m) (C=C, Ph), 1362 (m), 1275 (m), 809 (m, C-Cl), 753, 692 (vs, C-H Aromatic out of plane). ^1^H NMR δ: 10.43 (s, 1H, OH), 9.88 (s, 1H, CONH), 7.79 (d, 2H, ^3^*J* = 8.2, H11), 7.57 (s, 1H, PhNH), 7.40 (m, 3H, H12, H13), 7.19 (d, 1H, ^3^*J* = 8.8, H17), 7.18 (dd, 2H, ^3^*J* = 7.6, ^3^*J* = 8.2, H8), 7.01 (d, 1H, ^3^*J* = 8.8, H16), 6.93 (s, 1H, H19), 6.90 (d, 2H, ^3^*J* = 8.2, H7), 6.89 (t, 2H, ^3^*J* = 7.6, H22), 6.81 (t, 1H, ^3^*J* = 7.6, H9), 6.56 (t, 1H, ^3^*J* = 7.6, H23), 6.10 (d, 2H, ^3^*J* = 7.6, H21), 5.77 (d, 1H, ^3^*J* = 12.3, H5), 4.97 (d, 1H, ^3^*J* = 12.3, H4). RMN ^13^C δ: 167.3 (CO), 154.0 (C15), 148.8 (C3), 148.0 (C20), 145.6 (C6), 132.2 (C10), 129.4 (C13), 129.2 (C8), 129.1 (C12), 128.9 (C17), 128.8 (C22), 126.3 (C11), 124.5 (C18), 122.9 (C14), 120.5 (C9), 118.6 (C23), 117.1 (C16), 114.9 (C7, C19), 112.2 (C21), 62.8 (C5), 54.7 (C4). Mass analysis [M-H]^+^ (m/z): 483.1587 found, 483.1588 calculated.

(±)-5-(2′-Hydroxy-5′-methoxyphenyl)-*N*′,1,3-triphenyl-4,5-dihydro-1*H*-pyrazole-4-carbohydrazide (**2c**). Synthesized as described for **2a** starting from 0.300 g (1.07 mmol) of **1c**, 0.35 mL of phenylhydrazine (3.6 mmol) to obtain 0.32 g (0.66 mmol, 63% yield, 98.86% purity) of a white fluorescent solid, mp = 205–206 °C. IR (cm^−1^): 3452 (w), 3306 (w), 3225 (w) (N-H, O-H), 1654 (m, C=O), 1595 (m), 1494 (s), 1446 (m) (C=C, Ph), 1427 (m, CH_3_), 1366 (m), 1220 (m), 773 (vs), 750 (s), 689 (vs) (C-H Aromatic out of plane). ^1^H NMR δ: 9.80 (d, 1H, ^3^*J* = 1.8, CONH), 9.64 (s, 1H, OH), 7.82 (d, 2H, ^3^*J* = 8.2, H11), 7.80 (s, 1H, PhNH), 7.45 (dd, 2H, ^3^*J* = 7.6, ^3^*J* = 8.2, H12), 7.40 (t, 1H, ^3^*J* = 7.6, H13), 7.18 (dd, 2H, ^3^*J* = 7.6, ^3^*J* = 8.2, H8), 6.90 (d, 2H, ^3^*J* = 8.2, H7), 6.89 (t, 3H, ^3^*J* = 7.6, H22), 6.82 (d, 1H, ^3^*J* = 7.6, H9), 6.81 (d, 1H, ^3^*J* = 7.6, H16), 6.78 (dd, 1H, ^3^*J* = 7.6, ^4^*J =* 3.0, H17), 6.60 (d, 1H, ^4^*J* = 3.0, H19), 6.57 (t, 1H, ^3^*J* = 7.6, H23), 6.08 (d, 1H, ^3^*J* = 7.6, H21), 5.60 (d, 1H, ^3^*J* = 11.7, H5), 4.95 (d, 1H, ^3^*J* = 11.7, H4), 3.41 (s, 3H, CH_3_). ^13^C NMR δ: 167.3 (CO), 151.9 (C15), 148.6 (C3), 148.5 (C20), 147.5 (C6), 145.5 (C18), 132.0 (C10), 129.0 (C13), 128.82 (C12), 128.77 (C8), 128.5 (C22), 125.9 (C11), 122.9 (C14), 120.0 (C16), 118.1 (C23), 115.6 (C19), 115.4 (C9), 114.7 (C7), 113.4 (C17), 111.9 (C21), 62.9 (C5), 55.1 (OMe) 54.4 (C4). Mass analysis [M-H]^+^ (m/z): 479.2087 found, 479.2083 calculated.

(±)-5-(5′-Bromo-2′-hydroxyphenyl)-*N*′,1,3-triphenyl-4,5-dihydro-1*H*-pyrazole-4-carbohydrazide (**2d**). Synthesized as described for **2a** starting from 0.300 g (0.911 mmol) of **1d**, 0.35 mL of phenylhydrazine (3.6 mmol) to obtain 0.16 g (0.30 mmol, 33% yield, 98.54% purity) of a white fluorescent solid, mp = 201–203 °C. IR (cm^−1^): 3338 (w), 3252 (br) (N-H, O-H), 1641 (m, C=O), 1596 (m), 1493 (s) 1415 (m) (C=C, Ph), 1362 (m), 1274 (m), 767 (s), 753, 692 (vs, C-H Aromatic out of plane). RMN ^1^H δ: 10.50 (s, 1H, CONH), 9.93 (s, 1H, OH), 7.86 (d, 2H, ^3^*J* = 7.0, H11), 7.62 (s, 1H, PhNH), 7.48 (t, 2H, ^3^*J* = 7.5, H12), 7.46 (t, 1H, ^3^*J* = 7.8, H13), 7.36 (dd, 1H, ^3^*J* = 8.8, ^4^*J* = 2.3, H17), 7.24 (t, 2H, ^3^*J* = 7.6, H8), 7.12 (d, 1H, ^4^*J* = 2.3, H19), 7.03 (d, 1H, ^3^*J* = 8.8, H16), 6.97 (d, 2H, ^3^*J* = 7.6, H7), 6.96 (t, 2H, ^3^*J* = 7.6, H22), 6.87 (t, 1H, ^3^*J* = 7.6, H9), 6.62 (t, 1H, ^3^*J* = 7.6, H23), 6.17 (d, 2H, ^3^*J* = 7.6, H21), 5.63 (d, 1H, ^3^*J* = 11.7, H5), 5.04 (d, 1H, ^3^*J* = 11.7, H4). ^13^C NMR δ: 167.1 (CO), 154.3 (C15), 148.6 (C3), 147.8 (C20), 145.4 (C6), 132.0 (C10), 131.8 (C19), 131.6 (C17), 129.3 (C13), 129.1 (C8), 129.0 (C12), 128.7 (C22), 126.2 (C11), 124.9 (C14), 120.3 (C9), 118.4 (C23), 117.5 (C16), 114.8 (C7), 112.0 (C21), 110.5 (C18), 62.6 (C5), 54.6 (C4). Mass analysis [M-H]^+^ (m/z): 527.1088 found, 527.1083 calculated.

(±)-5-(2′,3′-Dihydroxyphenyl)-*N*′,1,3-triphenyl-4,5-dihydro-1*H*-pyrazole-4-carbohydrazide (**2e**). Synthesized as described for **2a** starting from 0.300 g (1.12 mmol) of **1e**, 0.5 mL of phenylhydrazine (5.08 mmol), and 5 drops of glacial acetic acid; after 48 h of reaction, 0.075 g (0.16 mmol, 15% yield, 100% purity) of a white fluorescent solid was obtained, mp. = 206–208 °C. IR (cm^−1^): 3531, 333, 3242 (br) (N-H, O-H), 1649 (m, C=O), 1596 (m), 1494 (s), 1477 (sh), 1366 (m), 1285 (s), 753. 691 (vs, C-H Aromatic out of plane). RMN ^1^H δ: 9.71 (s, 1H, CONH), 9.54 (s, 1H, OH), 8.89 (s, 1H, OH), 7.79 (d, 2H, ^3^*J* = 7.2, H11), 7.49 (s, 1H, PhNH), 7.42 (t, 2H, ^3^*J* = 7.2, H12), 7.39 (t, 1H, ^3^*J* = 7.1, H13), 7.14 (dd, 2H, ^3^*J* = 8.4, ^3^*J* = 7.5, H8), 6.93 (d, 2H, ^3^*J* = 7.9, H7), 6.90 (t, 2H, ^3^*J* = 7.9, H22), 6.77 (t, 1H, ^3^*J* = 7.5, H9), 6.74 (d, 1H, ^3^*J* = 8.0, H19), 6.53 (t, 1H, ^3^*J* = 7.4, H23), 6.50 (d, 1H, ^3^*J* = 8.5, H17), 6.38 (d, 1H, ^3^*J* = 7.9, H18), 6.08 (d, 2H, ^3^*J* = 7.9, H21), 5.63 (d, 1H, ^3^*J* = 11.8, H5), 4.93 (d, 1H, ^3^*J* = 11.8, H4). RMN ^13^C δ: 167.5 (CO), 149.0 (C15), 147.6 (C16), 145.9 (C3), 145.3 (C20), 145.2 (C6), 132.5 (C10), 129.2 (C13), 129.1 (C12), 129.0 (C8), 128.9 (C22), 126.2 (C11), 123.1 (C14), 120.1 (C9, C17), 119.2 (C18), 118.4 (C23), 115.0 (C7), 114.8 (C19), 112.3 (C21), 63.3 (C5), 54.7 (C4). Mass analysis [MH+] (m/z): 465.1903 found, 465.1927 calculated.

(±)-5-(2′,4′-Dihydroxyphenyl)-*N*′,1,3-triphenyl-4,5-dihydro-1*H*-pyrazole-4-carbohydrazide (**2f**). Synthesized as described for **2a** starting from 0.300 g (1.12 mmol) of **1f**, 0.35 mL of phenylhydrazine (3.6 mmol), to obtain 0.16 g (0.34 mmol, 30% yield, 98.36% purity) of a white fluorescent solid, mp = 206–208 °C. IR (cm^−1^): 3241 (br) (N-H, O-H), 1649 (m, C=O), 1606 (sh), 1596 (m) 1494 (s) (C=C, Ph), 1463 (m), 1364 (m), 1217 (m), 772, 752, 692 (vs, C-H Aromatic out of plane). ^1^H NMR δ: 9.79 (s, 1H, OH), 9.71 (d, 1H, ^3^*J* = 1.8, CONH), 9.25 (s, 1H, OH), 7.76 (d, 2H, ^3^*J* = 8.2, H11), 7.52 (d, 1H, ^3^*J* = 1.8, PhNH), 7.41 (dd, 2H, ^3^*J* = 7.1, ^3^*J* = 8.2, H12), 7.37 (t, 1H, ^3^*J* = 7.1, H13), 7.13 (t, 2H, ^3^*J* = 7.6, H8), 6.94 (d, 2H, ^3^*J* = 7.6, H7), 6.90 (t, 2H, ^3^*J* = 7.6, H22), 6.76 (t, 1H, ^3^*J* = 7.6, H9), 6.76 (d, 1H, ^3^*J* = 8.2, H19), 6.53 (t, 1H, ^3^*J* = 7.6, H23), 6.47 (d, 1H, ^3^*J* = 2.3, H16), 6.07 (d, 2H, ^3^*J* = 7.6, H21), 6.00 (dd, 1H, ^3^*J* = 8.2, ^4^*J* = 2.3, H18), 5.52 (d, 1H, ^3^*J* = 12.0, H5), 4.83 (d, 1H, ^3^*J* = 12.0, H4). ^13^C NMR δ: 167.8 (CO), 158.3 (C17), 155.8 (C15), 148.9 (C3), 147.5 (C20), 145.9 (C6), 132.6 (C10), 130.4 (C19), 129.11 (C13), 129.01 (C12), 129.0 (C8), 128.7 (C22), 126.1 (C11), 120.0 (C9), 118.4 (C23), 115.0 (C7), 112.6 (C14), 112.3 (C21), 107.2 (C18), 102.5 (C16), 62.9 (C5), 54.7 (C4). Mass analysis [M-H]^+^ (m/z): 465.1925 found, 465.1927 calculated.

(±)-5-(2′,5′-Dihydroxyphenyl)-*N*′,1,3-triphenyl-4,5-dihydro-1*H*-pyrazole-4-carbohydrazide (**2g**). Synthesized as described for **2a** starting from 0.300 g (1.12 mmol) of **1g** and 0.5 mL of phenylhydrazine (5.08 mmol) to obtain after 48 h of reaction 0.093 g (0.20 mmol, 18% yield, 99.43% purity) of a white fluorescent solid, mp = 206–208 °C. IR (cm^−1^): 3493 (w), 3302 (br), 3246 (br) (N-H, O-H), 1647 (m, C=O), 1595 (m), 1493 (s), 1453 (m) (C=C, Ph), 1369 (m), 1201 (m), 774 (vs), 748 (vs), 690 (vs), 675 (sh) (C-H Aromatic out of plane). ^1^H NMR δ: 9.77 (s, 1H, CONH), 9.34 (s, 1H, OH), 8.51 (s, 1H, OH), 7.80 (d, 2H, ^3^*J* = 8.2, H11), 7.55 (s, 1H, PhNH), 7.44 (t, 2H, ^3^*J* = 7.1, H12), 7.41 (t, 1H, ^3^*J* = 7.1, H13), 7.18 (t, 2H, ^3^*J* = 7.6, H8), 6.95 (d, 1H, ^3^*J* = 7.6, H16), 6.90 (t, 2H, ^3^*J* = 7.6, H22), 6.83 (d, 2H, ^3^*J* = 7.6, H7), 6.80 (t, 1H, ^3^*J* = 7.6, H9), 6.57 (dd, 1H, ^3^*J* = 7.6, ^4^*J* = 2.9, H17), 6.54 (t, 1H, ^3^*J* = 7.6, H23), 6.51 (d, 1H, ^3^*J* = 2.9, H19), 6.13 (d, 2H, ^3^*J* = 7.6, H21), 5.55 (d, 1H, ^3^*J* = 12.3, H5), 4.95 (d, 1H, ^3^*J* = 12.3, H4). RMN ^13^C δ: 167.2 (CO), 149.7 (C15), 148.6 (C18), 147.4 (C3), 147.0 (C20), 145.5 (C6), 132.0 (C10), 128.9 (C13), 128.72 (C8), 128.70 (C12), 128.5 (C22), 125.9 (C11), 122.5 (C14), 119.8 (C9), 118.1 (C17), 115.7 (C19), 115.6 (C7), 115.3 (C23), 114.6 (C16), 111.9 (C21), 63.0 (C5), 54.2 (C4). Mass analysis [M-H]^+^ (*m*/*z*): 465.1932 found, 465.1927 calculated.

(±)-5-(5′-Bromo-2′-hydroxy-3-methoxyphenyl)-*N*′,1,3-triphenyl-4,5-dihydro-1*H*-pyrazole-4-carbohydrazide (**2h**). Synthesized as described for **2a**, from 0.300 g (0.83 mmol) of **1h**, 0.35 mL of phenylhydrazine (3.6 mmol) to obtain 0.12 g (0.21 mmol, 26% yield, 99.29% purity) of a white fluorescent solid, mp. = 207–210 °C. IR (cm^−1^): 3500 (br), 3370, 3290 (br) (N-H, O-H), 1650 (m, C=O), 1600 (m), 1490 (s), 1440 (m) (C=C, Ph), 1420 (m, CH_3_), 1370 (m), 1270 (s), 750, 692 (vs, C-H Aromatic out of plane). ^1^H NMR δ: 9.88 (br, 1H, CONH), 9.70 (br, 1H, OH), 7.81 (d, 2H, ^3^*J* = 8.2, H11), 7.56 (s, 1H, PhNH), 7.44 (dd, 2H, ^3^*J* = 8.2, ^4^*J* = 7.6, H12), 7.42 (t, 1H, ^3^*J* = 7.6, H13), 7.20 (dd, 2H, ^3^*J* = 8.0, ^3^*J* = 7.6, H8), 7.08 (d, 1H, ^4^*J* = 2.3, H17), 6.93 (dd, 2H, ^3^*J* = 7.7, ^3^*J* = 7.0, H22), 6.92 (d, 2H, ^3^*J* = 8.0, H7), 6.83 (t, 1H, ^3^*J* = 7.6, H9), 6.74 (d, 1H, ^4^*J* = 2.3, H19), 6.60 (t, 1H, ^3^*J* = 7.0, H23), 6.13 (d, 2H, ^3^*J* = 7.7, H21), 5.62 (d, 1H, ^3^*J* = 12.0, H5), 5.00 (d, 1H, ^3^*J* = 12.0, H4), 3.90 (s, 3H, OCH_3_). ^13^C NMR δ: 167.3 (CO), 148.8 (C16), 148.75 (C15), 148.0 (C3), 145.6 (C20), 143.5 (C6), 132.1 (C10), 129.4 (C13), 129.3 (C12), 129.2 (C8), 128.8 (C22), 126.3 (C11), 124.9 (C14), 123.5 (C19), 120.5 (C9), 118.7 (C23), 114.8 (C7), 114.2 (C17), 112.1 (C21), 110.4 (C18), 62.7 (C5), 56.6 (OMe), 54.8 (C4). Mass analysis [M-H]^+^ (m/z): 557.1195 found, 557.1188 calculated.

(±)-5-(3′-Ethoxy-2′-hydroxyphenyl)-*N*′,1,3-triphenyl-4,5-dihydro-1*H*-pyrazole-4-carbohydrazide (**2i**). Synthesized as described for **2a** from 0.300 g (1.01 mmol) of **1i**, 0.35 mL of phenylhydrazine (3.6 mmol) to obtain 0.16 g (0.32 mmol, 32% yield, 99.50% purity) of a white fluorescent solid, mp. = 205–207 °C. IR (cm^−1^): 3496 (w), 3320 (br), 3258, 3058 (br) (N-H, O-H), 1650 (m, C=O), 1600 (m), 1490 (m) 1470 (m) (C=C, Ph), 1440 (m, CH_3_), 1370 (m), 1270 (m), 750 (vs), 690 (vs), 650 (m) (C-H Aromatic out of plane). ^1^H NMR δ: 9.75 (s, 1H, CONH), 9.00 (s, 1H, OH), 7.81 (d, 2H, ^3^*J* = 8.2, H11), 7.55 (s, 1H, PhNH), 7.45 (dd, 2H, ^3^*J* = 8.2, ^3^*J* = 7.6, H12), 7.42 (t, 1H, ^3^*J* = 7.6, H13), 7.16 (dd, 2H, ^3^*J* = 8.2, ^3^*J* = 7.6, H8), 6.93 (d, 2H, ^3^*J* = 8.2, H7), 6.93 (d, 1H, ^3^*J* = 8.2, H17), 6.88 (dd, 2H, ^3^*J* = 8.2, ^4^*J* = 7.7, H22), 6.79 (t, 1H, ^3^*J* = 7.6, H9), 6.66 (d, 1H, ^3^*J* = 7.6, H19), 6.56 (t, 1H, ^3^*J* = 7.7, H23), 6.54 (dd, 1H, ^3^*J* = 7.6, ^3^*J* = 8.2, H18), 6.05 (d, 2H, ^3^*J* = 8.2, H21), 5.67 (d, 1H, ^3^*J* = 12, H5), 4.95 (d, 1H, ^3^*J* = 12, H4), 4.14 (q, 2H, ^3^*J* = 7.0, CH_2_), 1.45 (t, 3H, ^3^*J* = 7.0, CH_3_). RMN ^13^C δ: 167.6 (CO), 148.9 (C16), 147.6 (C15), 146.8 (C3), 145.8 (C20), 144.0 (C6), 132.4 (C10), 129.2 (C13), 129.1 (C12), 129.0 (C8), 128.7 (C22), 126.2 (C11), 122.9 (C14), 121.4 (C19), 120.2 (C9), 119.2 (C18), 118.4 (C23), 114.9 (C7), 112.2 (C21), 112.1 (C17), 64.4 (OCH_2_), 63.1 (C5), 54.7 (C4), 15.3 (CH_3_). Mass analysis [M-H]^+^ (m/z): 493.2244 found, 493.2240 calculated.

### 3.4. Modelling and In Silico Studies

#### 3.4.1. Docking Simulations

Enantiomers (*4S*, *5S*) and (*4R*, *5R*) of DPCH derivatives, Figure S54, were drawn using CHEMSKETCH program 11.12; atomic connectivity was checked with GAUSS VIEW 3.0 and then geometrically optimized using Gaussian 09W at the AM1 level [93]. The catalytic domain-2 of HDAC6 (DD2-HDAC6) (PDB: 5G0J) was retrieved from previous work [94]. The 3D structure of DD2-HDAC6 was prepared using AutoDock Tools 1.5.6 [95]; polar hydrogen atoms and Kollman [96] charges were assigned for receptor and ligands. Validation of the method was performed with TSA with a root-mean-square deviation (RMSD) value of 2.05 Å, Figure S55. The grid box was centered on the receptor with grid points in the *x*, *y*, and *z* of 126 Å^3^, with a grid spacing of 0.375 Å^3^. A Lamarckian genetic algorithm was used as a scoring sample for a randomized population of 100 individuals, on which a 10^7^ energy evaluations were done; 100 runs were performed. A focused molecular docking at Zn^+2^ coordinates was performed using AutoDock 4.2 and AutoDock4Zn force field, which has improved parameters to dock zinc proteins [97]. The most populated cluster conformations and the lowest free energy of binding values (ΔG_b_°) were selected as the most representative. Docking results of the DD2-HDAC6-ligand complexes were analyzed using AutoDock Tools 1.5.6 [98]. Figures were further processed using Pymol v.099 [99].

#### 3.4.2. Theoretical ADME-Tox and Physicochemical Properties

The proposed compounds were submitted to determine their ADME-Tox properties on OSIRIS DataWarrior (v04.06.01) and Osiris Property Explorer [100]. The molecules were drawn using ChemBioDraw Ultra 12.0, and the simplified molecular input line entry specification (SMILES) codes for all compounds were obtained. The following properties were obtained from OSIRIS Property Explorer: mutagenic, tumorigenic, irritant, and reproductive effects—likewise, solubility in water (LogS) and topological surface area (TPSA) values. In the case of the Lipinski’s rules properties, these were determined from OSIRIS DataWarrior: molecular weight (MW), octanol–water partition coefficient (LogP), hydrogen acceptors (HA), hydrogen donors (HD), and rotatable bonds (RB). The size was measured by molar refractivity (MR) [68] and lipophilicity by the partition coefficient [101] parameters that were determined on ACD/ChemSketch and CS ChemDraw Pro v.6 software, respectively. All these biological, toxic, and physicochemical properties were compared with tubacin, TSA, and SAHA.

#### 3.4.3. QSAR Analysis

QSAR was performed under QSAR-2D [81,102]. Estimation of the lipid solubility descriptor (π) values was performed by means of the following equation: π = log(*P_X_/P_H_*), where *P*_x_ and *P*_H_ are the partition coefficients of the substituted and leading compounds, respectively. The Hammett constant in the *para* position (σ*_p_*) was utilized as the criterion of electronic effects [103]. Estimation of the steric descriptor (*E*_S_) values [104,105] was performed by means of the following equation: *E_S_* = log(*MR_X_*/*MR_H_*), where *MR*_x_ and *MR*_H_ are the molar refractivity values of the substituted and the leading compounds, respectively. The correlations were carried out through second-order polynomial regression analyses (*y* = *Ax*^2^ + *Bx* + *C*). The equation constants and parabolic correlation coefficient were analyzed under the Student’s test. The differences were considered significant for a minimal value of *p* < 0.05. Statistical tests were performed on Sigma Stat 3.5 software (Jandel Corp. SPSS INC. San Rafael, CA, USA).

### 3.5. In Vitro Assays

#### 3.5.1. Cell Culture

The cancer cell lines used in this study were obtained from the American Type Tissue Culture Collection (ATCC), Rockville, MD, USA. MCF-7 and MDA-MB-231 are from BC cells, and 3T3/NIH and MCF10A cells were included as nonmalignant cells. BC cell lines and fibroblasts were grown in Dulbecco’s modified Eagle Medium (DMEM) high-glucose with phenol red. The culture medium was supplemented with 10% fetal bovine serum (FBS, BioWest, Miami, FL, USA) as well as 100 U/mL penicillin and 100 mg/mL streptomycin as antibiotic. MCF10A cells were cultured in DMEM/F-12 supplemented with 5% horse serum (Biowest, Miami, FL, USA), 20 ng/mL epidermal growth factor, 10 mg/mL insulin, and 500 ng/mL hydrocortisone. Cell cultures were incubated at 37 °C in a humidified atmosphere of 5% CO_2_ and 95% air. Cells were grown until 80% confluence, treated with trypsin-EDTA (1%) (4 mL, 5 min, 37 °C), and then collected with medium (4 mL). Cells were centrifuged (3 × 10^3^ rpm, 10 min) and resuspended in medium (1–3 mL) and counted with CytoSmart cell counting (CytoSmart Technologies, Eindhoven, The Netherlands). Each cell line (10 × 10^3^ cells per well) was cultured in 96-well plates and allowed to attach for 24 h before the assays. Then, cells were treated with the tested compounds at different concentrations (10–120 µM) for 48 h; all compounds were dissolved in DMSO to produce a final concentration of DMSO of (0.1%).

#### 3.5.2. Cell Proliferation Assays

Cell proliferation was determined using the MTT [3-(4,5-dimethylthiazol-2-yl)-2,5-diphenyl-tetrazolium bromide, Sigma] assay. For this purpose, MTT (0.500 mg mL^−1^), dissolved in phosphate buffered saline (PBS), was added to each well (after aspirating the medium) and incubated for 3 h at 37 °C in 5% CO_2_. The MTT/PBS was removed, and 100 µL of DMSO was added to each well to dissolve the formazan crystals. Absorbance was measured with a microplate reader (ThermoScientific, Multiskan^TM^ Sky) at a wavelength of 550 nm. The quantity of formazan produced is directly proportional to the number of living cells. Results are expressed as the percentage of viable cells ± standard deviation in relation to the control (cell culture medium with 0.1% of DMSO), whose viability was designated as 100%. Each data point was performed in octuplicate in three independent experiments, and the results were reported as the mean absorption ± SD.

#### 3.5.3. In Vitro HDAC6 Inhibition

The HDAC6 activity was measured using the Fluor de Lys-HDAC6 assay kit (ENZO Life Sciences). The method consists of deacetylation of the substrate (Fluor de lys-SIRT1) in the presence of human recombinant HDAC6. Then, the deacetylated substrate is incubated at room temperature for 45 min with Fluor de Lys-developer II to generate a fluorophore that can be measured by fluorescence (Fluorescence Spectrometer LS 55 PerkinElmer) at an excitation/emission wavelength of 360/460 nm. The HDAC6 inhibition by TSA was determined using different concentrations (0.05, 5, 50, and 250 nM), while the inhibition by compound **2b** was evaluated with five concentrations (0.5, 5, 10, 35, and 50 µM). The HADC6 activity was expressed in percentage, and it was calculated with the following equation:

%HDAC6 Activity = absorbance of inhibition × 100/ absorbance of control

#### 3.5.4. Confocal Fluorescence Microscopy

An aliquot of MCF-7 breast cancer cells was seeded in petri dishes with a coverslip in clear media (supplemented media phenol-red-free). The cells were incubated at 37 °C overnight in 5% CO_2_. Once the cells were adhered, the tested compound was added at 10 µM concentrations for 30 min. Then, the cells were washed several times with PBS and immersed in cold ethanol. Micrographs were acquired with confocal laser scanning microscope LSM 710 NLO, Carl Zeiss, Germany.

#### 3.5.5. Wound Closure Assay

BC cells (1.5 × 10^5^) cells were 24-well plated and allowed to reach 100% confluence. Cell monolayers were scratched with a 200 µL sterile pipette tip to form wound gaps, and the media and cell debris were carefully aspirated. Culture media was replaced and compounds **2b** and **2c** at 15 µM were added. The wound closure was monitored by microscopy at 16, 24, and 48 h. The wound area was measured by quadruplicate in two independent experiments and expressed as percentage of the control (cells culture medium with 0.1% of DMSO).

#### 3.5.6. DPPH Assay (2,2-Diphenyl-1-picrylhydrazyl)

Into a 96-well plate, we poured 100 μL of DPPH 0.20 mM in absolute methanol and 100 μL of the appropriate compound (6.25, 12.5, 25, 50, 100, 200 µM final concentrations) dissolved in DMSO, the mixtures were incubated for 30 min at room temperature protected from light [106]. Each assay was performed in triplicate with ascorbic acid (AA) as a standard. The absorbance was measured at 517 nm in a transparent 96-well test microplate (Multiskan-EX Thermo Scientific, Waltham, MA, USA). The results are shown as percentage of DPPH radical reduced at each concentration. Therefore, the antioxidant activity (DPPH scavenging) of each compound was calculated by the following equation: [1 − (A_1_ − A_2_)/(A_DPPH_ − A_S_)] × 100, where: A_1_ = absorbance of the compound with DPPH, A_2_ = absorbance of the compound, A_DPPH_ = absorbance of DPPH (diluted 1:1 with solvent) and A_S_ = absorbance of the solvent. The experiments were performed in triplicate with several concentrations and the IC_50_ values were calculated using GraphPad Prism 8.

### 3.6. Statistical Analysis

Where needed, results were compared by one-way ANOVA with Dunnett post-test. GraphPad Prism version 8 for Windows was used for statistical analysis. A difference was considered statistically significant if *p* ≤ 0.05. The half-maximal inhibitory concentration (IC_50_) was calculated from the dose–response curves through a logarithmic analysis of HillSlope.

## 4. Conclusions

In summary, the synthesis and chemical characterization of nine new 4,5-dihydropyazole-carbohydrazide derivatives with dual antioxidant and antiproliferative activities on BC cell lines are described. The synthesized compounds had more favorable physicochemical and ADME-Tox characteristics than tubacin, but were comparable to TSA and SAHA, the known HDAC6 inhibitors. An antiproliferative effect against cancer cell lines MCF-7 and MDA-MB-231, as well as low cytotoxicity against normal breast cells, was demonstrated. In particular, compounds with R = H (**2a**), 6-OMe (**2c**), and 8-OEt (**2i**) showed the smallest IC_50_ values against BC cells and the smallest cytotoxicity towards nonmalignant breast cells, being capable of crossing the cell membrane. Furthermore, compounds **2b** (6-Cl) and **2c** (6-OMe) diminished the motility of TNBC cells and inhibited the human HDAC6 with free binding energies like TSA and SAHA. QSAR supported a size effect, probably by blocking the entrance of the DD2 catalytic domain, with close similarity to the mode of action of tubacin. Finally, these compounds are effective dual anticancer–antioxidant agents with reduced cytotoxicity in healthy cells. Further studies on other HDACs isoforms are currently in progress.

## Figures and Tables

**Table 1:** Physicochemical properties of **2a**–**i** and prediction of oral absorption (%ABS) compared with the reference compounds (tubacin, TSA, and SAHA).

Comp.	MW	LogP	HA	HD	RB	VLR	LogS	TPSA [Å^2^]	MR [cm^3^/mol^−1^]	%ABS
**2a**	448.52	2.7	6	3	6	0	−5.0	77.0	132.1	82
**2b**	482.97	3.3	6	3	6	0	−5.73	77.0	136.7	82
**2c**	478.55	2.7	7	3	7	0	−5.01	86.2	139.4	80
**2d**	527.42	3.4	6	3	6	1	−5.83	77.0	139.8	82
**2e**	464.52	2.2	7	4	6	0	−4.70	97.2	133.9	76
**2f**	464.52	2.2	7	4	6	0	−4.70	97.2	133.9	76
**2g**	464.52	2.2	7	4	6	0	−4.70	97.2	133.91	76
**2h**	557.44	3.0	7	3	7	1	−5.85	86.2	147.0	80
**2i**	492.57	2.6	7	3	8	0	−5.31	86.2	144.2	80
Tubacin	721.87	7.2	10	4	16	3	−9.41	168.4	200.0	51
TSA	302.37	2.2	5	2	6	0	−3.26	69.6	91.7	85
SAHA	264.32	2.3	5	3	8	0	−3.33	78.4	70.6	82

Abbreviations: MW = molecular weight (g mol^−1^); LogP = logarithm of octanol–water partition coefficient; HA = hydrogen acceptors; HD = hydrogen donors; RB = rotatable bonds; VLR = violations of Lipinski’s rules; LogS = logarithm of the solubility in water; TPSA = topological polar surface area; %ABS = absorption percentage; NA = not applicable.

**Table 2:** Cytotoxic activity of **2a**–**i** and reference compounds (IC_50_ µM) on several cell lines.

Compounds	MCF-7	MDA-MB-231	3T3/NIH	MCF-10A
**2a**	41 ± 5	60 ± 2	>100	>100
**2b**	35 ± 3	24 ± 2	19 ± 2	33 ± 4
**2c**	26 ± 1	33 ± 3	>100	>100
**2d**	24 ± 2	26 ± 1	25 ± 3	22 ± 3
**2e**	24 ± 3	32 ± 3	32 ± 2	55 ± 5
**2f**	28 ± 2	33 ± 2	36 ± 2	45 ± 4
**2g**	23 ± 1	33 ± 3	28 ± 2	30 ± 3
**2h**	101 ± 2	108 ± 1	103 ± 2	>100
**2i**	71 ± 5	64 ± 4	>100	>100
TSA	0.5 ± 2	0.4 ± 1	1 ± 2	0.2 ± 0.06
SAHA	8 ± 1	2.5 ± 1	10 ± 2	12 ± 1.5

## Data Availability

Data is contained within the article and Supplementary Materials.

## Supplementary Materials

The following are available online at https://www.mdpi.com/article/10.3390/ph15060690/s1, Figure S1: NOE spectra of compound 5-(2-hydroxyphenyl)-*N*′,1,3-triphenyl-4,5-dihydro-1*H*-pyrazole-4-carbohydrazide (**2a**); Figure S2: Binding conformation around DD2-HDAC6 catalytic domain obtained through blind docking; Figures S3–S4: Half-maximal inhibitory concentration 50 (IC_50_) in BC cell lines MCF-7 and MDA-MB-231; Figures S5–S8: Antiproliferative activity in the non-malignant cell lines 3T3/NIH and MCF10A; Figure S9: (a) π values and σ_H_ values of DPCH derivatives on healthy cellular line 3T3/NIH; Figure S10: Comparison of the radical-scavenging activity of compounds **2a**–**i** and ascorbic acid; Figures S11–S28: ^1^H and ^13^C NMR spectra of compound **2a**–**i** in DMSO-d6; Figures S29–S30: COSY and HETCOR spectra of compound 5-(2-hydroxyphenyl)-*N*′,1,3-triphenyl-4,5-dihydro-1H-pyrazole-4-carbohydrazide (**2a**); Figures S31–S39: IR spectra of compounds **2a**–**i**; Figures S40–S44: Mass spectra of compounds **2a**–**i**; Figures S45–S53: HPLC chromatograms-purity of compounds **2a**–**i**; Figure S54: Enantiomers (4S, 5S) A and (4R, 5R) B of modelled 4,5-dihydropyrazole derivatives; Figure S55: Overlay of TSA in the DD2-HDAC6 domain with an RMSD value of 2.05; Table S1: Bond lengths (Å), Bond and torsion angles (°) of **2a**; Table S2: Hydrogen bonding geometry parameters of **2a**; Table S3: Free binding energy ΔGb° (kcal/moL) and Kd (µM) values obtained by docking the DD2-HDAC6 domain with 4,5-dihydropyrazole derivatives **2a**–**i**; Table S4: Interactions among of the 4,5-dihydropyrazole derivatives compared with tubacin, TSA, and SAHA with the DD2-HDAC6 structure (PDB: 5G0J); Table S5: Toxicity profile of the 4,5-dihydropyrazole derivatives compared with tubacin and TSA; Table S6: Crystal data and details of the structure determination for **2a**.
