# Supplementary material for: Dihydropyrazole-Carbohydrazide Derivatives with Dual Activity as Antioxidant and Anti-Proliferative Drugs on Breast Cancer Targeting the HDAC6
Source: Pharmaceuticals (Basel). 2022 May 31;15(6):690. doi: 10.3390/ph15060690 (PMC9230091; doi:10.3390/ph15060690)
Supplement: Supplementary file 1 [file pharmaceuticals-15-00690-s001.zip › pharmaceuticals-1687615-supplementary.pdf]

SUPPLEMENTARY MATERIAL

Dihydropyrazole-carbohydrazide derivatives with dual activity as antioxidant and anti-proliferative drugs on breast cancer targeting the HDAC6

Irving Balbuena-Rebolledo[1,2,3], Astrid M. Rivera-Antonio[1,2], Yudibeth Sixto-Lopez[3], José Correa-Basurto[3*], Martha C. Rosales-Hernández[2], Jessica Elena Mendieta-Wejebe[2], Francisco J. Martínez-Martínez[4], Ivonne María Olivares-Corichi[5], José Rubén García-Sánchez[5], Juan Alberto Guevara-Salazar[6], Martiniano Bello[3], Itzia I. Padilla-Martínez[1*]

1 Laboratorio de Química Supramolecular y Nanociencias, Unidad Profesional Interdisciplinaria de Biotecnología, Instituto Politécnico Nacional, Avenida Acueducto s/n, Barrio la Laguna Ticomán, Ciudad de México 07340, México.

2 Laboratorio de Biofísica y Biocatálisis, Sección de Estudios de Posgrado e Investigación, Escuela Superior de Medicina, Instituto Politécnico Nacional, Plan de San Luis y Salvador Díaz Mirón s/n, Casco de Santo Tomas, Ciudad de México 11340, México.

3 Laboratorio de Diseño y Desarrollo de Nuevos Fármacos e Innovación Biotecnológica, Escuela Superior de Medicina, Instituto Politécnico Nacional, Plan de San Luis y Díaz Mirón, s/n, Col. Casco de Santo Tomas, 11340, Ciudad de México, México.

4 Facultad de Ciencias Químicas, Universidad de Colima, Km. 9 Carretera Colima-Coquimatlán, C.P. 28400, Coquimatlán, Colima, México.

5 Laboratorio de Oncología Molecular y Estrés Oxidativo de la Escuela Superior de Medicina, Instituto Politécnico Nacional, Plan de San Luis y Díaz Mirón, s/n, Col. Casco de Santo Tomas, 11340, Ciudad de México, México.

6 Departamento de Farmacología. Escuela Superior de Medicina, Instituto Politécnico Nacional, Plan de San Luis y Díaz Mirón, S/N, México, D.F. 11340, México.

* Correspondence: jcorreab@ipn.mx (J.C.-B.); ipadillamar@ipn.mx

List of contents:

Preparation, spectroscopic and physico-chemical characterization of compounds **1a-i**

1. **Figure S1.** NOE spectra of compound 5-(2-hydroxyphenyl)-N',1,3-triphenyl-4,5-dihydro-1*H*-pyrazole-4-carbohydrazide (**2a**).
2. **Figure S2.** Binding conformation around DD2-HDAC6 catalytic domain obtained through blind docking. Compounds: (a) **2c**, (b) **2d**, (c) **2f**, (d) **2g**, (e) **2h** and (f) **2i**, compounds are depicted as cyan ball and stick, while interacting residues as green sticks, Zn is depicted as yellow sphere. Figure was built with PyMOL 0.99rc6.
3. **Figures S3-S4.** Half-maximal inhibitory concentration 50 (IC_{50}) in BC cell lines MCF-7 and MDA-MB-231.
4. **Figures S5-S8.** Antiproliferative activity in the non-malignant cell lines 3T3/NIH and MCF10A.
5. **Figure S9.** (a) π values and σ_H values of DPCH derivatives on healthy cellular line 3T3/NIH.
6. **Figure S10.** Comparison of the radical-scavenging activity of compounds **2a-i** and ascorbic acid
7. **Figures S11-S28.** ^{1}H and ^{13}C NMR spectra of compound **2a-i** in DMSO-d6.
8. **Figures S29-S30.** COSY and HETCOR spectra of compound 5-(2-hydroxyphenyl)-N',1,3-triphenyl-4,5-dihydro-1*H*-pyrazole-4-carbohydrazide (**2a**).
9. **Figures S31-S39.** IR spectra of compounds **2a-i**.
10. **Figures S40-S44.** Mass spectra of compounds **2a-i.**
11. **Figure S45-S53.** HPLC chromatograms-purity of compounds **2a-i**.
12. **Figure S54.** Enantiomers (*4S*, *5S*) A and (*4R*, *5R*) B of modelled 4,5-dihydropyrazole derivatives.
13. **Figure S55.** Overlay of TSA in the DD2-HDAC6 domain with an RMSD value of 2.05.

14. **Table S1.** Bond lengths (Å), Bond and torsion angles (°) of **2a**.
15. **Table S2.** Hydrogen bonding geometry parameters of **2a**
16. **Table S3.** Free binding energy $\Delta G_b°$ (kcal/moL) and K_d (µM) values obtained by docking the DD2-HDAC6 domain with 4,5-dihydropyrazole derivatives **2a-i**. **A** is the (*4S*, *5S*) and **B** is the (*4R*, *5R*) enantiomer.
17. **Table S4.** Interactions among of the 4,5-dihydropyrazole derivatives compared with tubacin, TSA and SAHA with the DD2-HDAC6 structure (PDB: 5G0J), obtained by docking studies.
18. **Table S5.** Toxicity profile of the 4,5-dihydropyrazole derivatives compared with tubacin and TSA.
19. **Table S6.** Crystal data and details of the structure determination for **2a**.

Preparation and spectroscopic and physicochemical characterization of compounds **1a-i**.

3-benzoyl-2*H*-1-benzopyran-2-one (**1a**).

It was obtained from 5.00 mL (46.7 mmol) of salicylaldehyde and 8.90 mL (51.4 mmol) of ethyl benzoyl acetate as pale-yellow solid: 10.5 g (42.0 mmol, 90 % yield), mp = 137-139 °C. ^{1}H NMR δ: 8.40 (s, 1H, H4), 7.91 (dd, 2H, ^3J = 8.2, ^4J = 1.3, H13), 7.84 (dd, 1H, ^3J = 7.8, ^4J = 1.8, H5), 7.71 (dt, 1H, ^3J = 8.0, ^4J = 1.8, H7), 7.68 (dd, 1H, ^3J = 7.5, 8.0, H15), 7.52 (t, 2H, ^3J = 7.7, H14), 7.47 (d, 1H, ^3J = 7.9, H8), 7.40 (t, 1H, ^3J = 7.5, H6). ^{13}C NMR δ: 192.2 (C11), 158.5 (C2), 154.6 (C9), 145.8 (C4), 136.5 (C12), 134.4 (C15), 134.0 (C7), 130.2 (C5), 130.0 (C13), 129.2 (C14), 126.8 (C3), 125.3 (C6), 118.7 (C10), 116.8 (C8).

3-benzoyl-6-chloro-2*H*-chromen-2-one (**1b**). $CDCl_3$

^{1}H NMR δ: 7.97 (s, 1H, H4), 7.86 (d, 2H, ^3J = 7.0 H13), 7.62 (t, 1H, ^3J = 7.5, H15), 7.58 (d, 1H, ^3J = 7.4, H7), 7.57 (s, 1H, H5), 7.48 (t, 2H, ^3J = 7.3, H14), 7.35 (d, 1H, ^3J = 9.2, H8). ^{13}C NMR δ: 191.1 (C11), 157.8 (C2), 153.0 (C9), 143.8 (C4), 131.0 (C6), 135.9 (C12), 134.0 (C7), 133.5 (C15), 131.0 (C6), 130.3 (C6), 129.6 (C13,C3), 129.5 (C7), 128.7 (C14), 128.2 (C5), 126.8 (C5), 119.1 (C10), 118.4 (C8), 118.0 (C8).

3-benzoyl-6-methoxy-2*H*-chromen-2-one (**1c**). $CDCl_3$

^{1}H NMR δ: 7.98 (s, 1H, H4), 7.82 (d, 2H, ^3J = 7.0, H13), 7.55 (t, 1H, ^3J = 7.4, H15), 7.41 (t, 2H, ^3J = 7.6, H14), 7.25 (d, 1H, ^3J = 8.8, H8), 7.16 (dd, 1H, ^3J = 8.8, ^4J = 2.6, H7), 6.97 (dd, 1H, ^4J = 3.0, H5). ^{13}C NMR

δ: 191.8 (C11), 158.7 (C2), 156.4 (C9), 149.2 (C6), 145.4 (C4), 136.2 (C12), 133.9 (C15), 129.6 (C13), 128.6 (C14), 127.1 (C3), 121.8 (C7), 118.5 (C10), 117.9 (C8), 110.7 (C5), 56.0 (OMe).

3-benzoyl-6-bromo-2*H*-chromen-2-one (**1d**). $CDCl_3$

^{1}H NMR δ: 7.96 (s, 1H, H4), 7.85 (d, 2H, ^3J = 8.2, H13), 7.61 (t, 1H, ^3J = 7.6, H15), 7.72 (s, 1H, H5), 7.70 (dd, 1H, ^4J = 2.3, ^3J = 8.2, H7), 7.47 (t, 2H, ^3J = 7.6, H14), 7.27 (m, 1H, H8). ^{13}C NMR δ: 191.1 (C11), 157.8 (C2), 153.5 (C9), 143.8 (C4), 136.3 (C7), 135.8 (C12), 134.1 (C15), 131.3 (C5), 129.6 (C13), 128.7 (C14), 128.0 (C3), 119.6 (C10), 118.7 (C8), 117.5 (C6).

3-benzoyl-8-hydroxy-2*H*-chromen-2-one (**1e**). $CDCl_3$

^{1}H NMR δ: 10.4 (br, 1H, OH), 8.34 (s, 1H, H4), 7.89 (d, 2H, ^3J = 8.2, H13), 7.66 (t, 1H, ^3J = 7.4, H15), 7.51 (t, 2H, ^3J = 7.6, H14), 7.24 (m, 1H, H6), 7.19 (m, 2H, H5, H7). ^{13}C NMR δ: 192.3 (C11), 158.4 (C2), 146.2 (C9), 145.1 (C4), 143.2 (C8), 136.6 (C12), 134.3 (C15), 129.9 (C13), 129.2 (C14), 126.7 (C6), 125.3 (C3), 120.2 (C5), 120.1 (C7), 119.6 (C10).

3-benzoyl-7-hydroxy-2*H*-chromen-2-one (**1f**). DMSO-d6

^{1}H NMR δ: 8.30 (s, 1H, H4), 7.81 (d, 2H, ^3J = 7.6, H13), 7.67 (d, 1H, ^3J = 8.8, H5), 7.62 (t, 1H, ^3J = 7.1, H15), 7.48 (t, 2H, ^3J = 7.6, 7.4, H14), 6.82 (d, 1H, ^3J = 8.2, H6), 6.75 (s, 1H, H8). ^{13}C NMR δ: 192.5 (C11), 163.8 (C2), 158.8 (C7), 157.1 (C9), 147.3 (C4), 137.2 (C12), 133.8 (C15), 132.1 (C5), 129.8 (C13), 129.0 (C14), 121.5 (C7), 114.4 (C6), 111.2 (C10), 102.5 (C8).

3-benzoyl-6-hydroxy-2*H*-chromen-2-one (**1g**). DMSO-d6

^{1}H NMR δ: 9.8 (br, 1H, OH), 8.28 (s, 1H, H4), 7.87 (d, 2H, ^3J = 8.2, H13), 7.66 (t, 1H, ^3J = 7.4, H15), 7.51 (t, 2H, ^3J = 7.6, H14), 7.31 (m, 1H, H8), 7.15 (s, 1H, H5), 7.14 (m, 1H, H7). ^{13}C NMR δ: 192.1 (C11), 158.4 (C2), 154.1 (C9), 147.5 (C4), 145.3 (C6), 136.0 (C15), 134.0 (C13), 129.5 (C14), 128.8 (C3), 126.3 (C5), 121.8 (C8), 118.7 (C7), 117.3 (C10), 113.5 (C8).

3-benzoyl-6-bromo-8-methoxy-2*H*-chromen-2-one (**1h**). DMSO-d6

^{1}H NMR δ: 8.34 (s, 1H, H4), 7.94 (d, 2H, H13, ^3J = 7.6), 7.71 (t, 1H, H15, ^3J = 7.6), 7.64 (d, 1H, H5, ^4J = 1.2), 7.58 (d, 1H, H7, ^4J = 1.2), 7.55 (t, 2H, H14, ^3J = 7.6). ^{13}C NMR δ: 191.4 (C11), 157.3 (C2), 147.4 (C9), 144.2 (C4), 142.9 (C8), 135.8 (C12), 134.1 (C15), 129.6 (C13), 128.8 (C14), 127.6 (C3), 122.6 (C7), 120.1 (C5), 118.2 (C10), 116.2 (C6), 56.8 (OCH_3).

3-benzoyl-8-ethoxy-2*H*-chromen-2-one (**1i**). $CDCl_3$

^{1}H NMR δ: 8.09 (s, 1H, H4), 7.91 (dd, 2H, ^{3}J = 8.2, ^{4}J = 1.2, H13), 7.64 (tt, 1H, ^{3}J = 7.4, ^{4}J = 1.2, H15), 7.50 (t, 2H, ^{3}J = 7.6, H14), 7.29 (t, 1H, J = 7.6, H6), 7.20 (d, 1H, ^{3}J = 7.6, ^{4}J = 1.8, H5), 7.18 (d, 1H, ^{3}J = 7.6, H7). 4.24 (q, 2H, ^{3}J = 7.1, OCH_2), 1.55 (t, 3H, ^{3}J = 7.1, CH_3). ^{13}C NMR δ: 191.8 (C11), 158.1 (C2), 146.6 (C8), 145.7 (C4), 144.6 (C9), 136.2 (C12), 133.8 (C15), 129.6 (C13), 128.6 (C14), 127.2 (C3), 124.8 (C6), 120.3 (C7), 118.9 (C10), 116.4 (C5), 65.1 (OCH_2), 14.7 (CH_3).

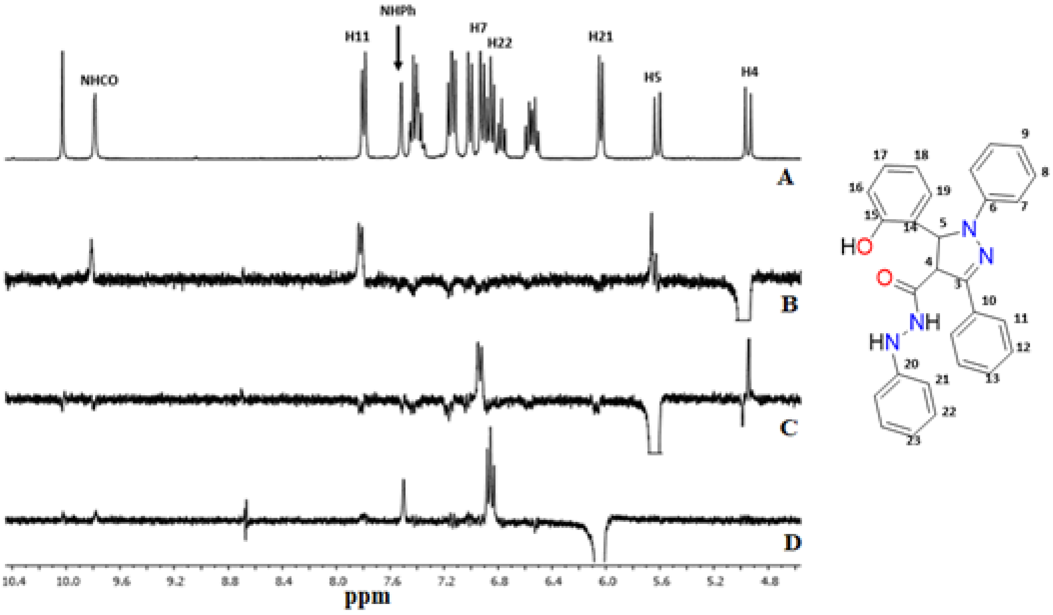

Figure S1. NOE spectra of compound 5-(2-hydroxyphenyl)-N',1,3-triphenyl-4,5-dihydro-1H-pyrazole-4-carbohydrazide (**2a**).

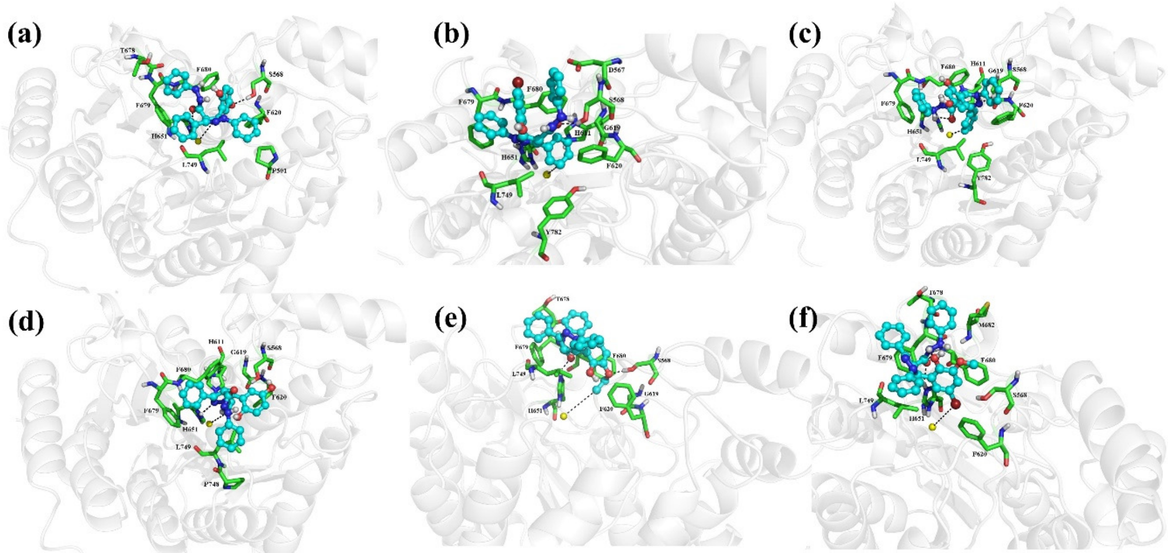

Figure S2. Binding conformation around DD2-HDAC6 catalytic domain obtained through blind docking. Compounds: (a) **2c**, (b) **2d**, (c) **2f**, (d) **2g**, (e) **2h** and (f) **2i**, compounds are depicted as cyan

ball and stick, while interacting residues as green sticks, Zn is depicted as yellow sphere. Figure was built with PyMOL 0.99rc6.

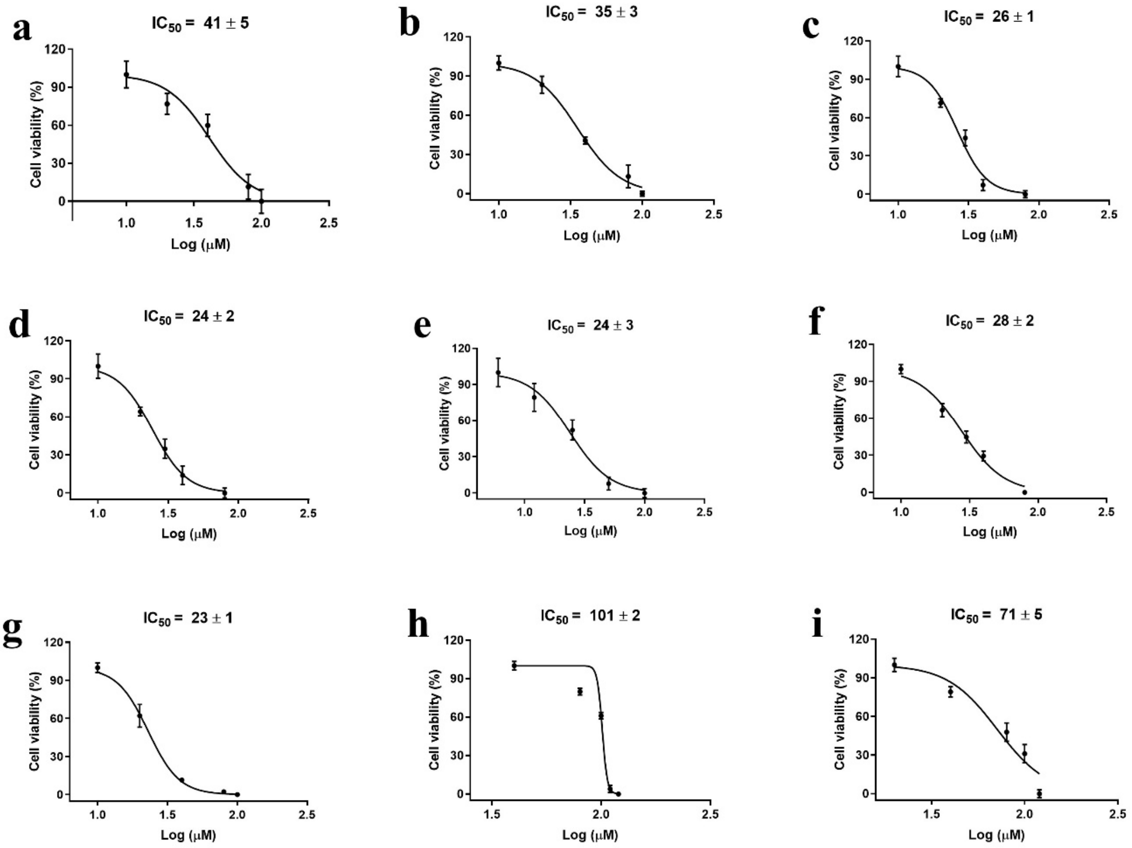

Figure S3. Inhibitory concentration 50 (IC_{50}) from the biological study in the breast cancer cell line MCF-7 (a) **2a**, (b) **2b**, (c) **2c**, (d) **2d**, (e) **2e**, (f) **2f**, (g) **2g**, (h) **2h** and (i) **2i**. All assays were performed in triplicate. The results are expressed as the mean ± SD (standard deviation) using Student´s t-test.

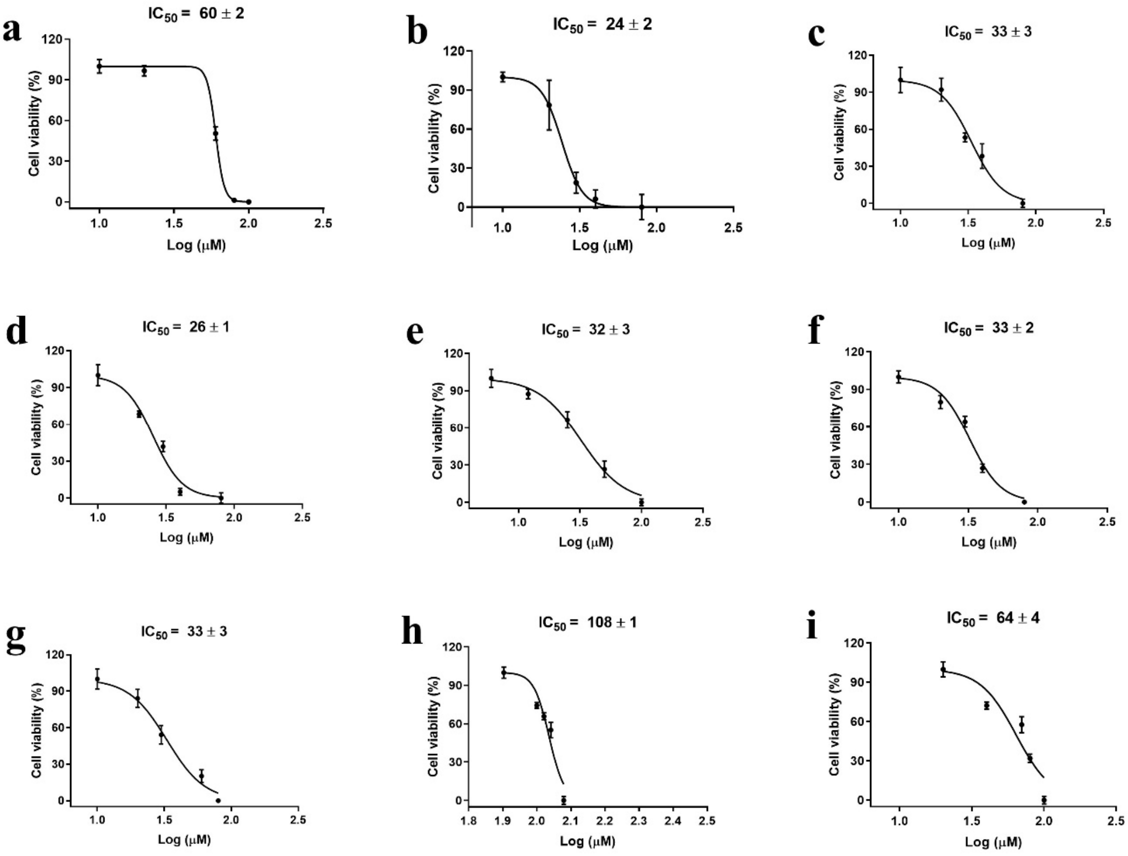

Figure S4. Inhibitory concentration 50 (IC_{50}) from the biological study in the breast cancer cell line MDA-MB-231 (a) **2a**, (b) **2b**, (c) **2c**, (d) **2d**, (e) **2e**, (f) **2f**, (g) **2g**, (h) **2h** and (i) **2i**. All assays were performed in triplicate. The results are expressed as the mean ± SD (standard deviation) using Student´s t-test.

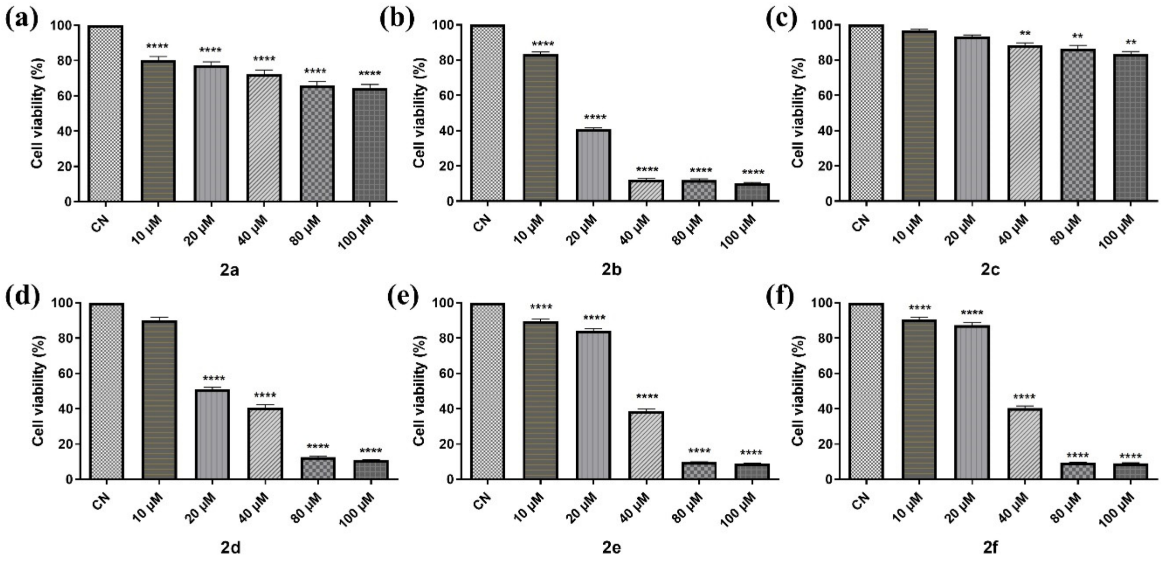

Figure S5. Antiproliferative activity from the biological study in the non-tumrigenic cell line 3T3/NIH (a) **2a**, (b) **2b**, (c) **2c**, (d) **2d**, (e) **2e**, (f) **2f**. All assays were performed in triplicate.

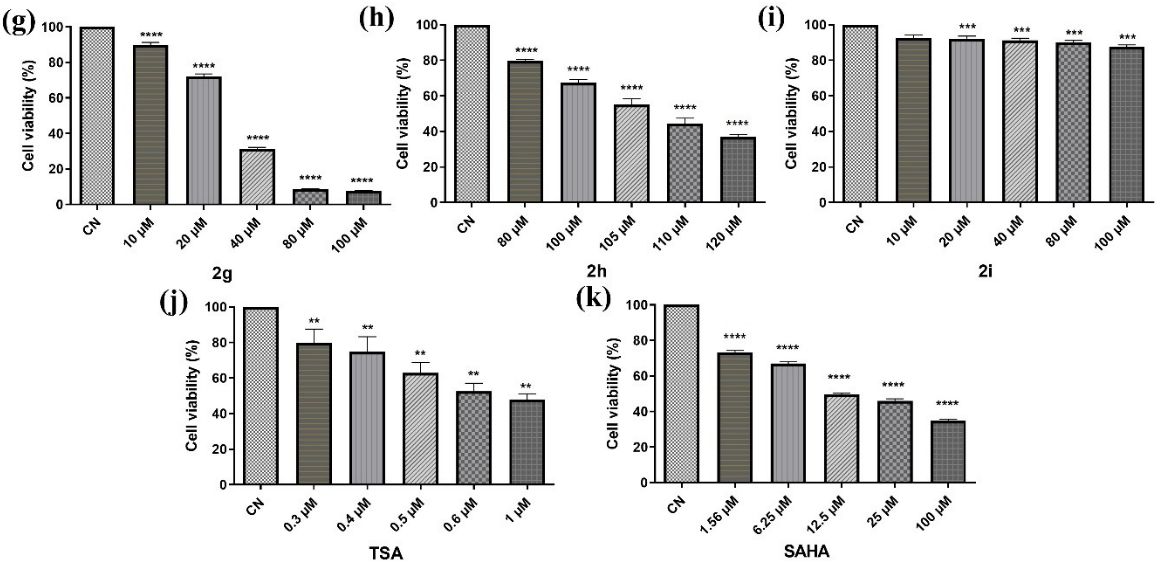

Figure S6. Antiproliferative activity from the biological study in the non-tumorigenic cell line 3T3/NIH (g) **2g**, (h) **2h** and (i) **2i**, (j) TSA, (k) SAHA. All assays were performed in triplicate.

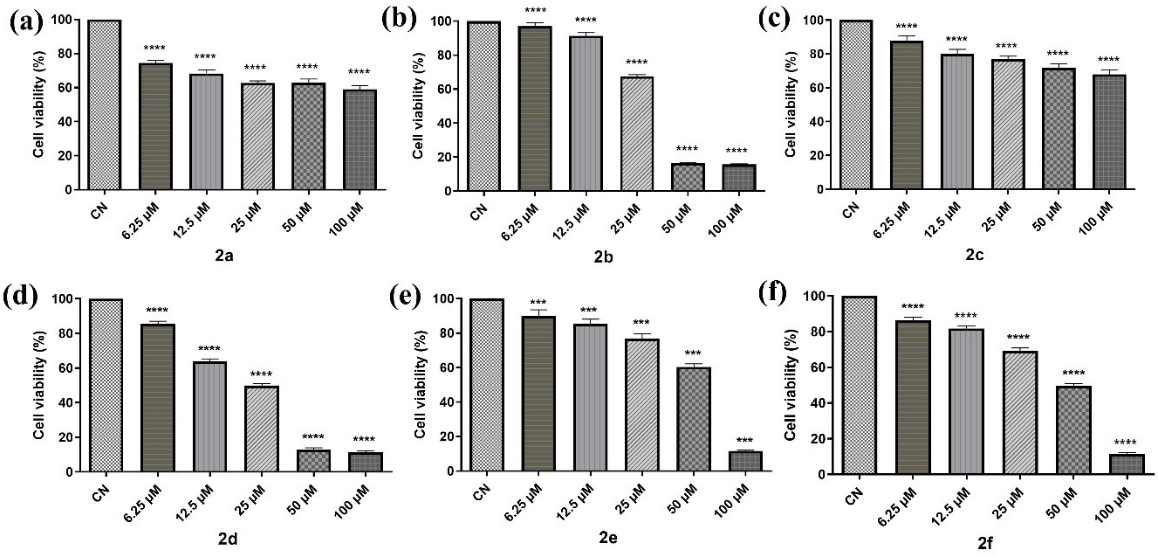

Figure S7. Antiproliferative activity from the biological study in the MCF10A cells (nonmalignant breast epithelial cells) (a) **2a**, (b) **2b**, (c) **2c**, (d) **2d**, (e) **2e**, (f) **2f**. All assays were performed in triplicate.

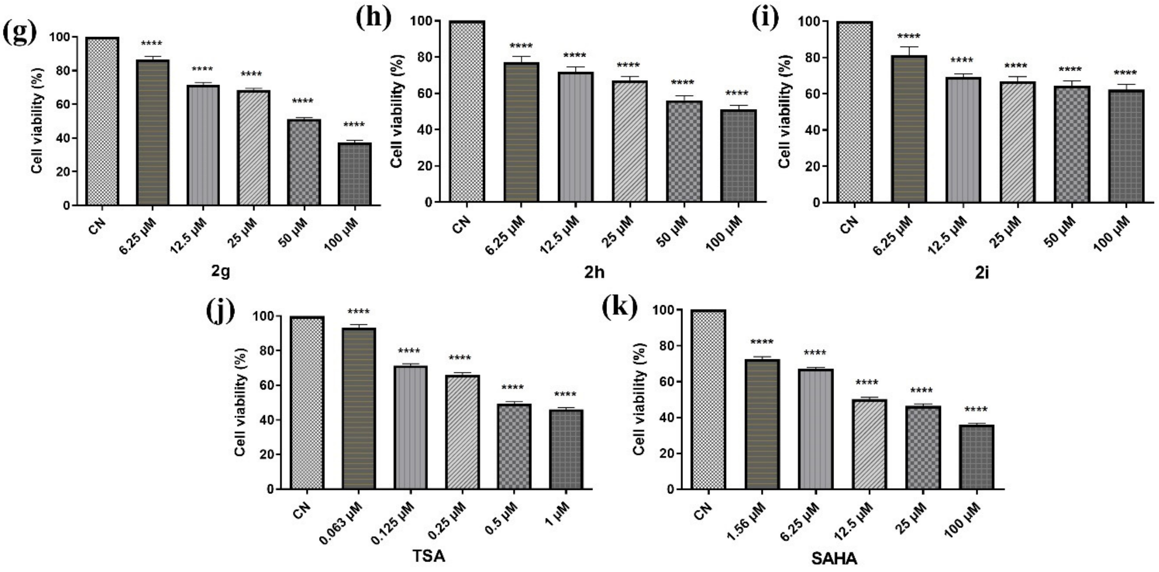

Figure S8. Antiproliferative activity from the biological study MCF10A cells (nonmalignant breast epithelial cells) (g) **2g**, (h) **2h** and (i) **2i**, (j) TSA, (k) SAHA. All assays were performed in triplicate.

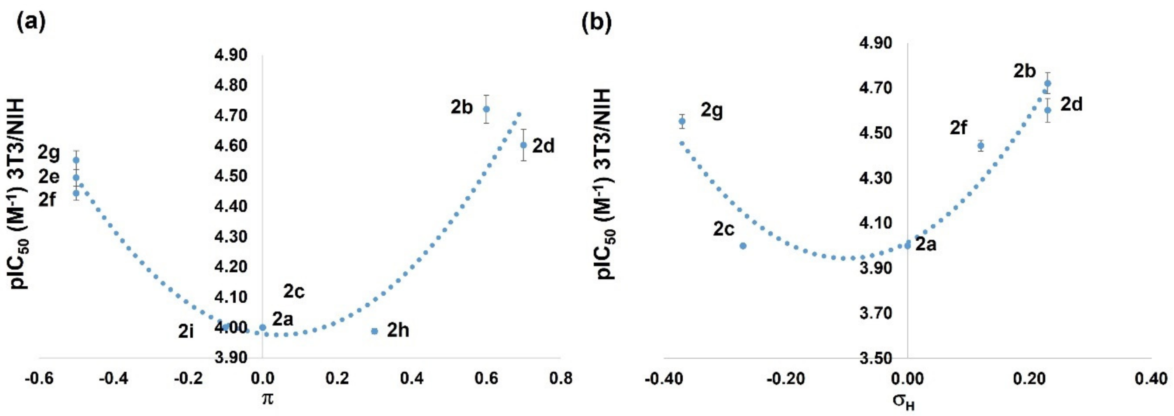

Figure S9. (a) π values and (b) σ_H values of DPCH derivatives on healthy cellular line 3T3/NIH.

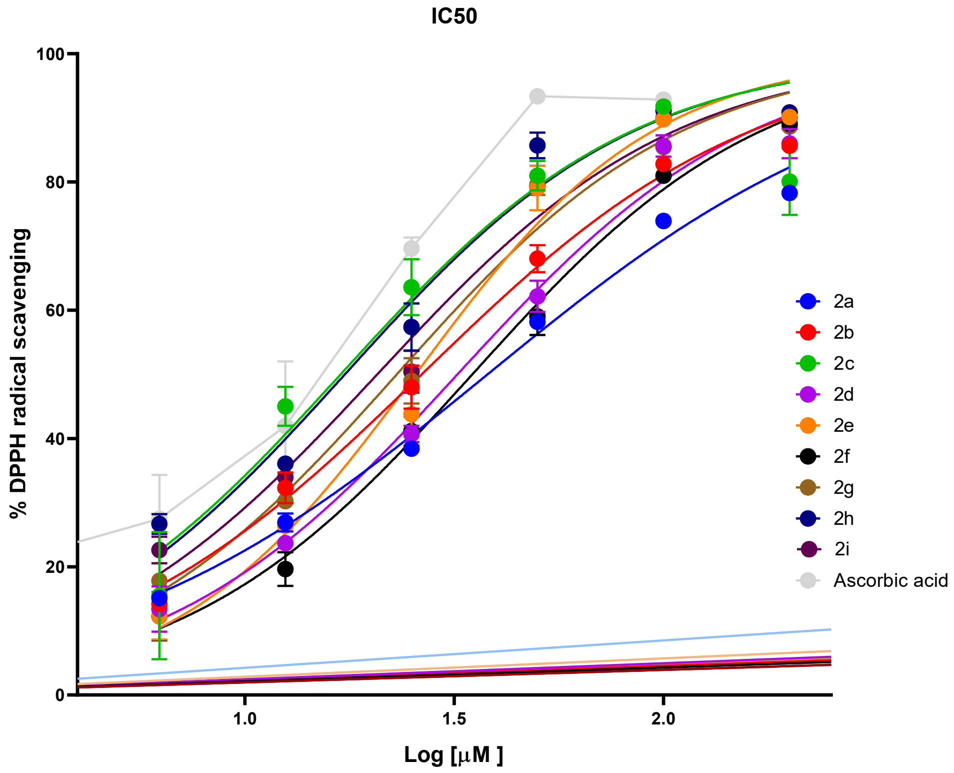

Figure S10. IC_{50} Comparison of the radical-scavenging activity of compounds **2a-i** at different concentrations and ascorbic acid as reference compound. All assays were performed in triplicate.

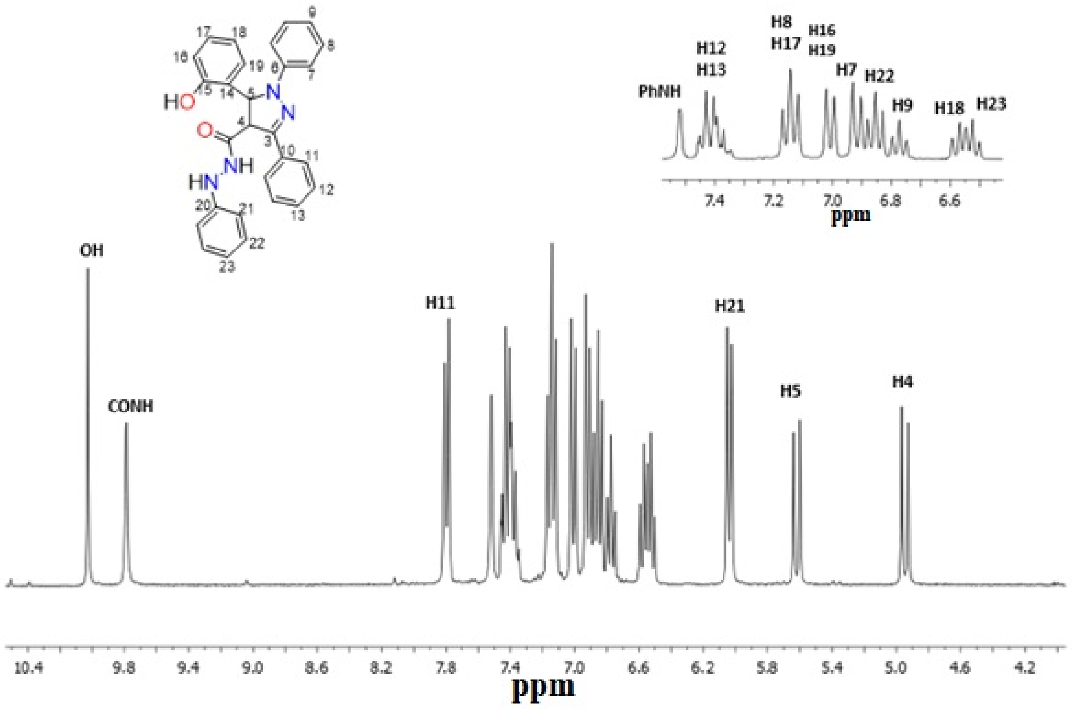

Figure S11. ^{1}H NMR spectrum of compound **2a**, DMSO-d6.

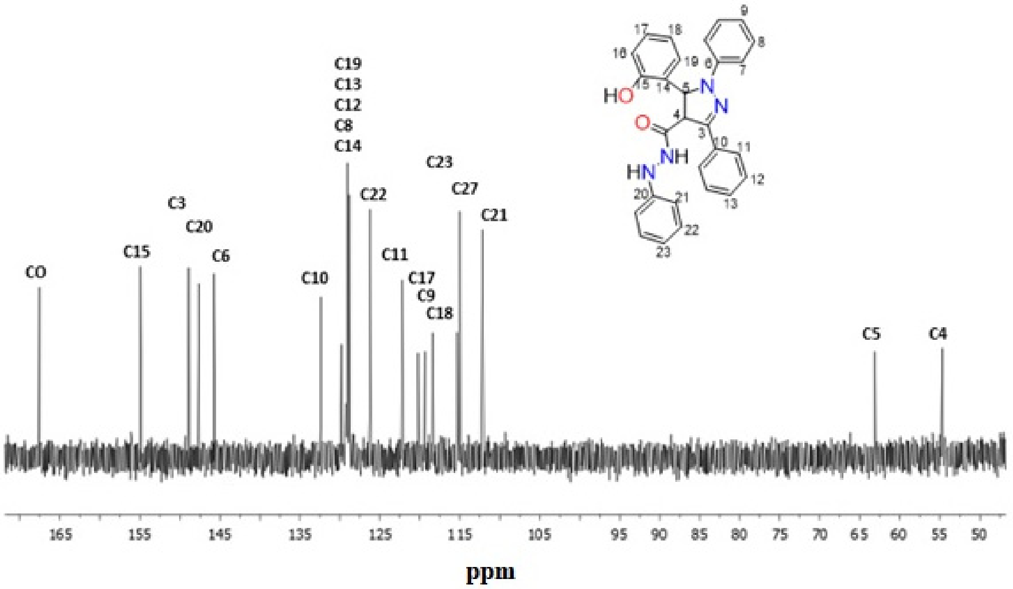

Figure S12. ^{13}C NMR spectrum of the compound **2a**, DMSO-d6.

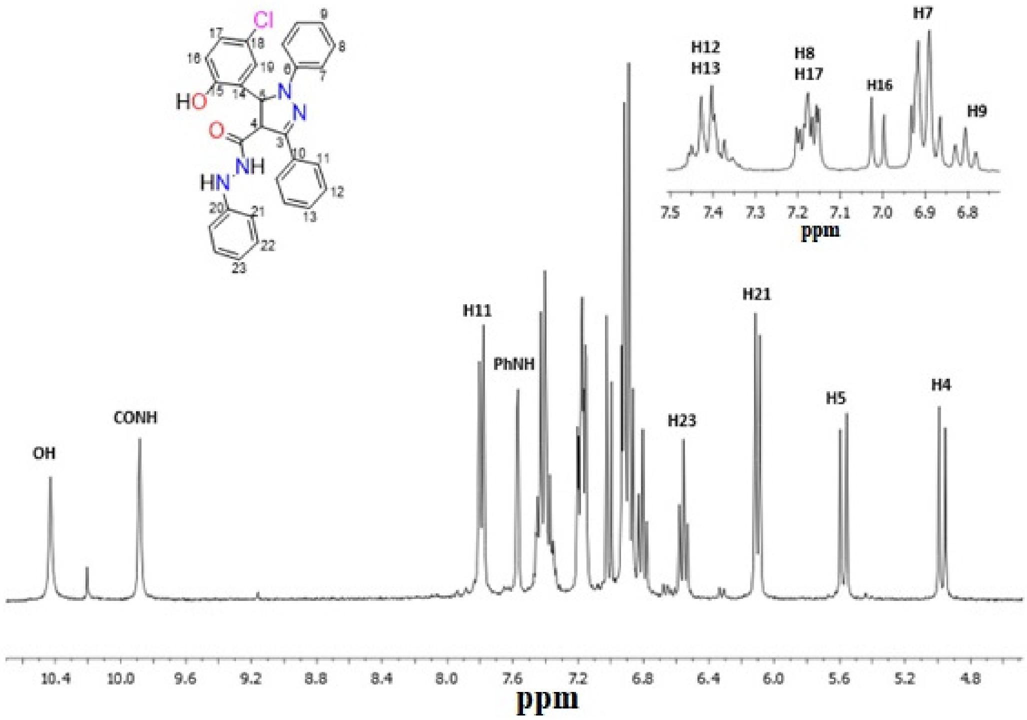

Figure S13. ^{1}H NMR spectrum of compound **2b**, DMSO-d6.

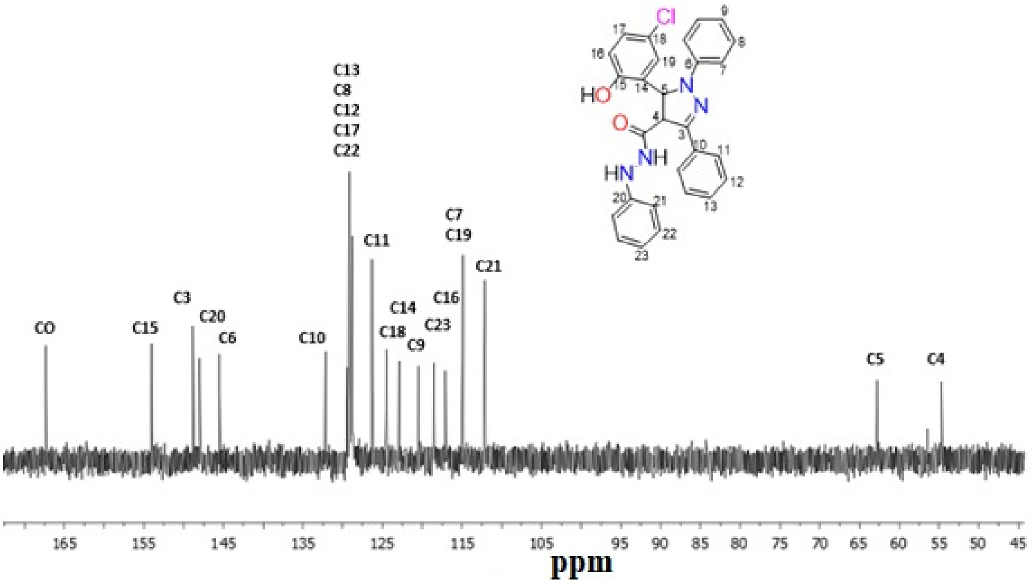

Figure S14. ^{13}C NMR spectrum of the compound **2b**, DMSO-d6.

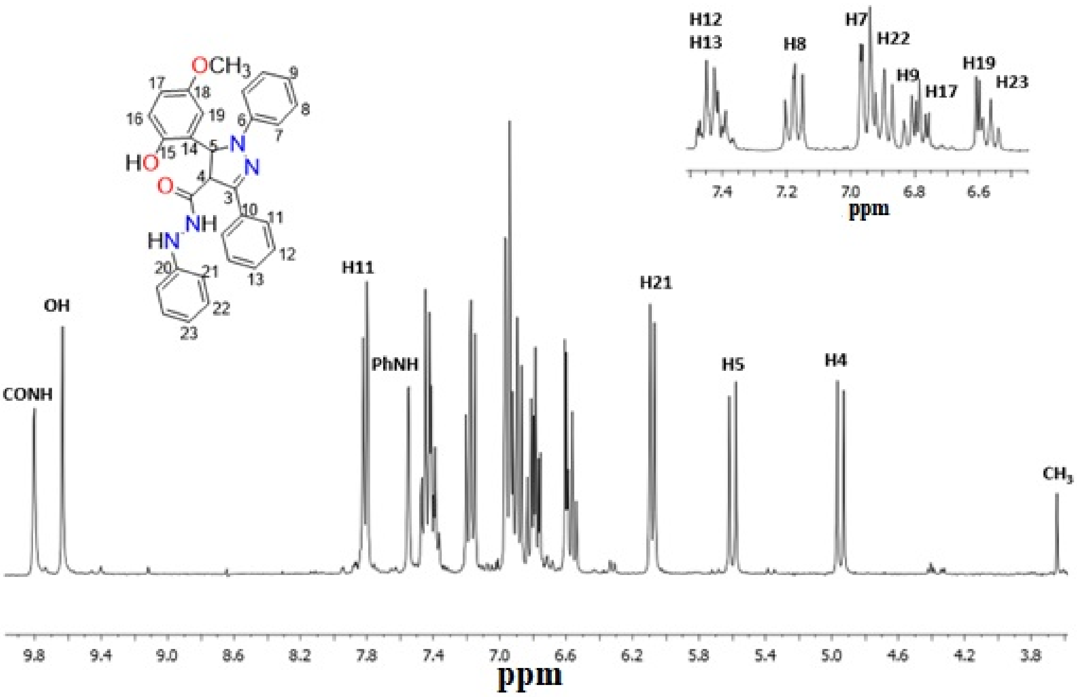

Figure S15. ^{1}H NMR spectrum of compound **2c**, DMSO-d6.

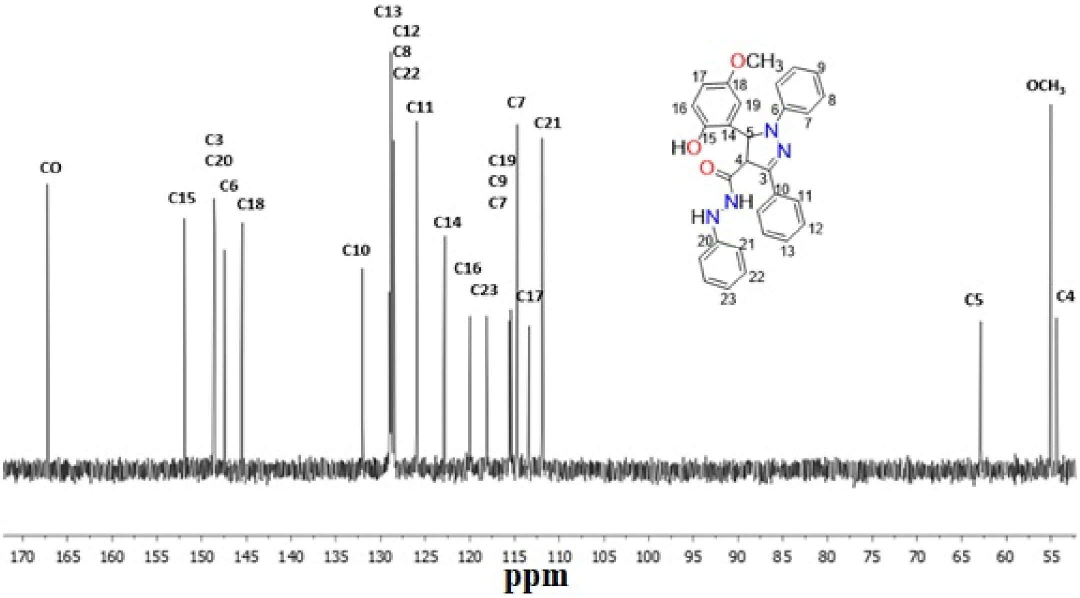

Figure S16. ^{13}C NMR spectrum of the compound **2c**, DMSO-d6.

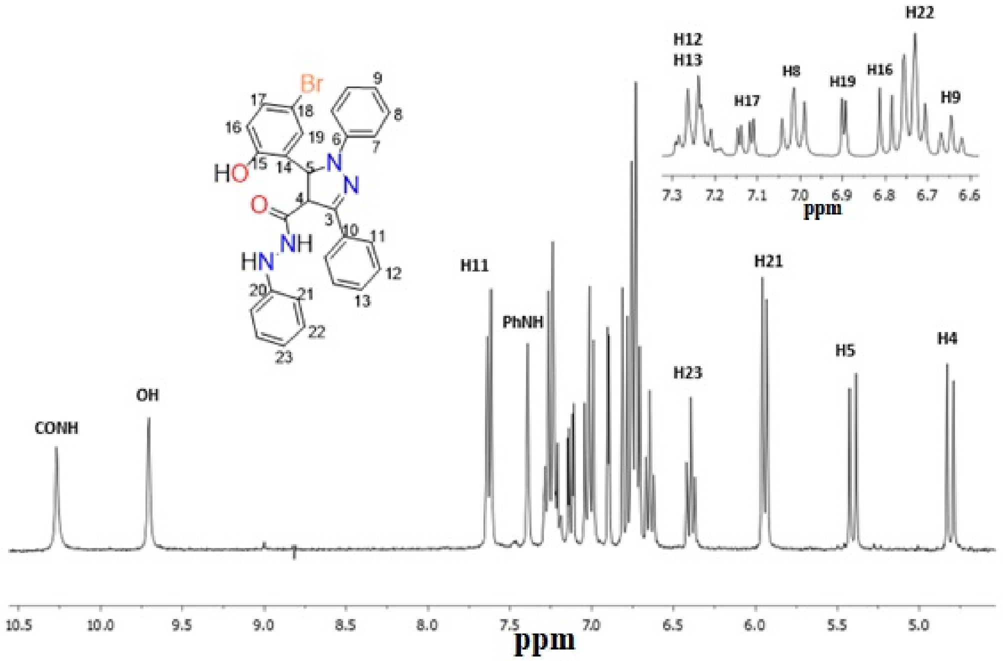

Figure S17. ^{1}H NMR spectrum of compound **2d**, DMSO-d6.

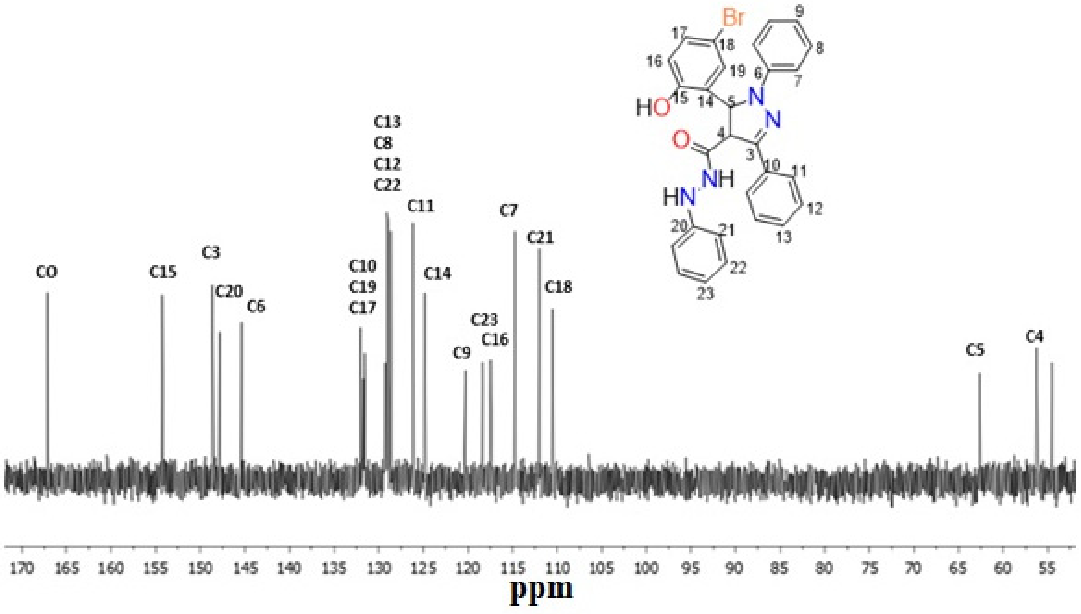

Figure S18. ^{13}C NMR spectrum of the compound **2d**, DMSO-d6.

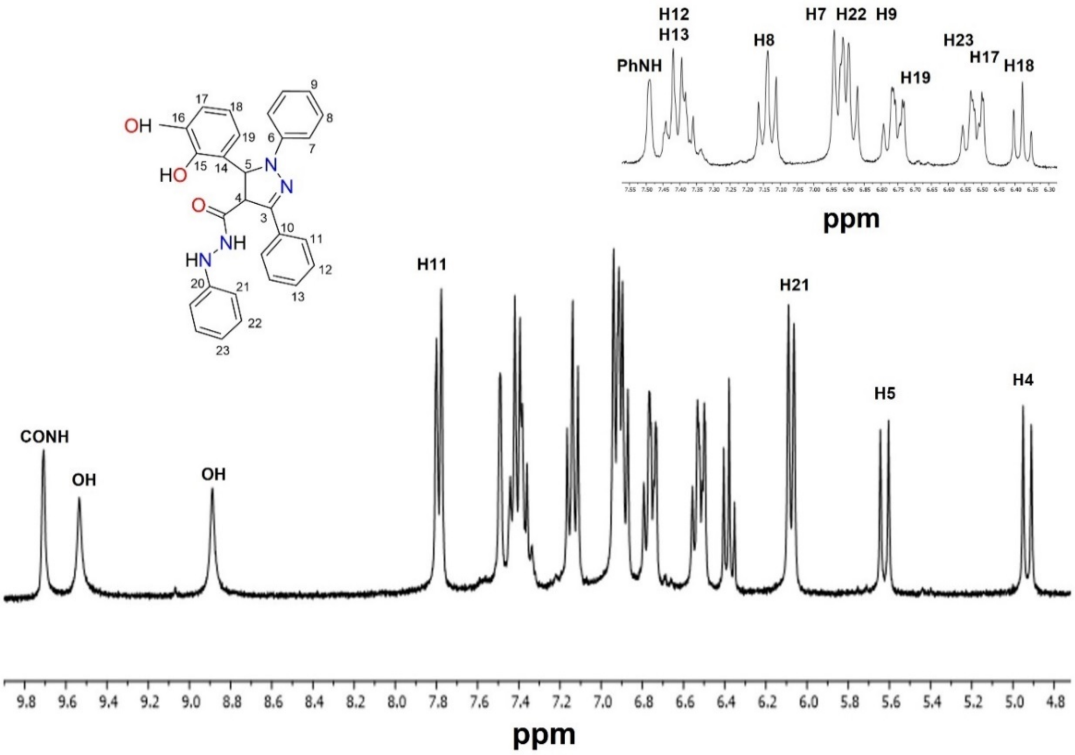

Figure S19. ^{1}H NMR spectrum of compound **2e**, DMSO-d6.

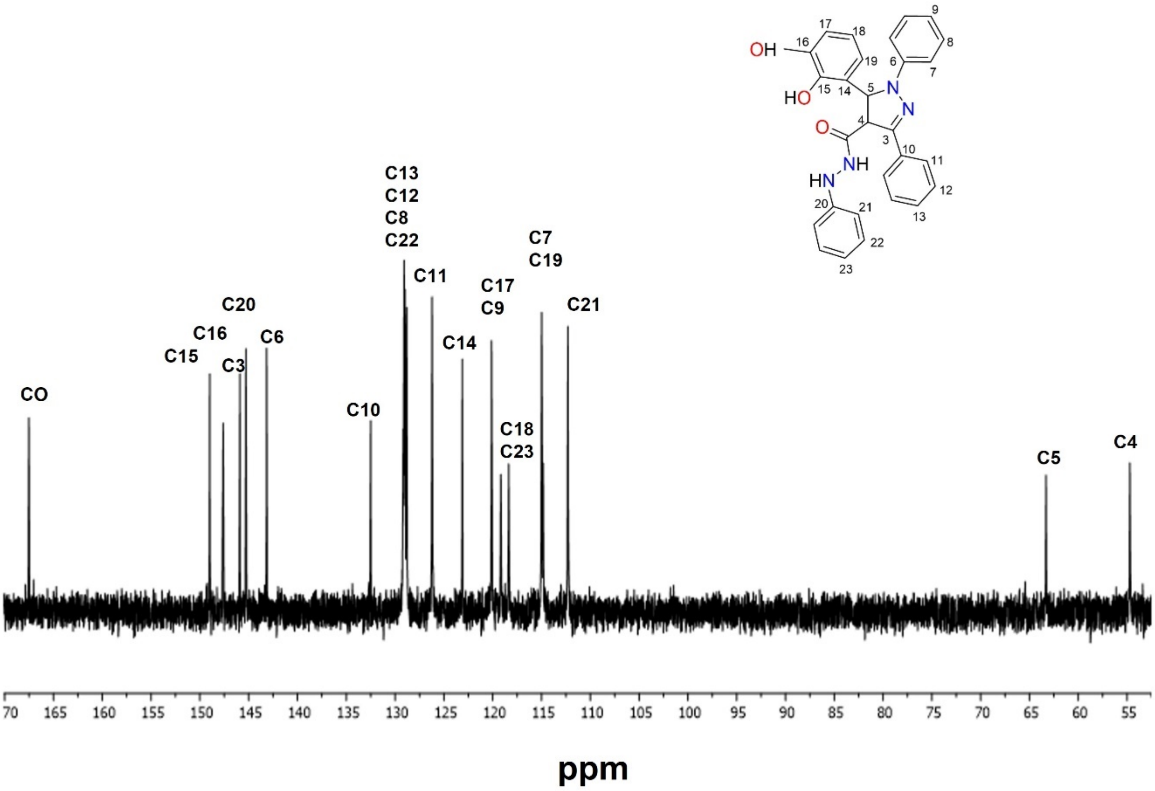

Figure S20. ^{13}C NMR spectrum of the compound **2e**, DMSO-d6.

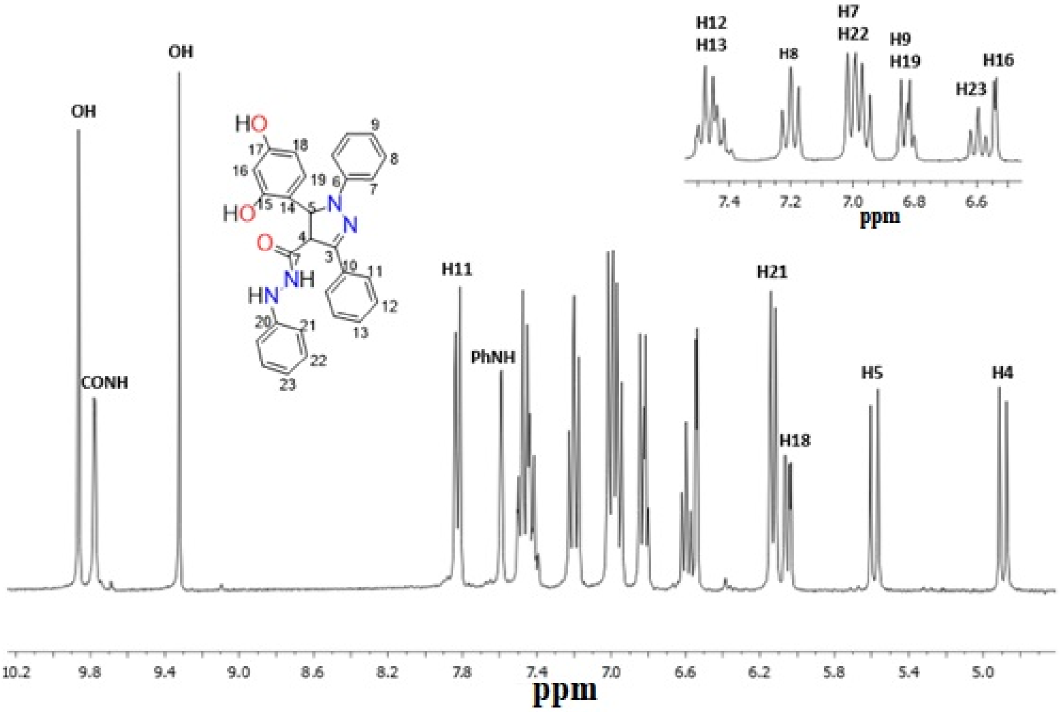

Figure S21. ^{1}H NMR spectrum of compound **2f**, DMSO-d6.

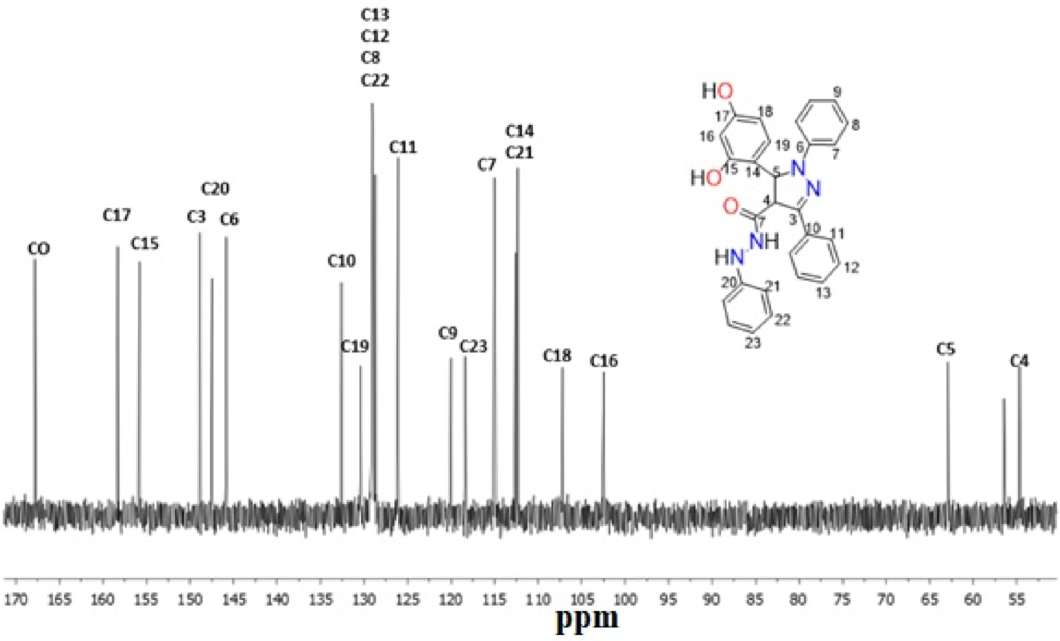

Figure S22. ^{13}C NMR spectrum of the compound **2f**, DMSO-d6.

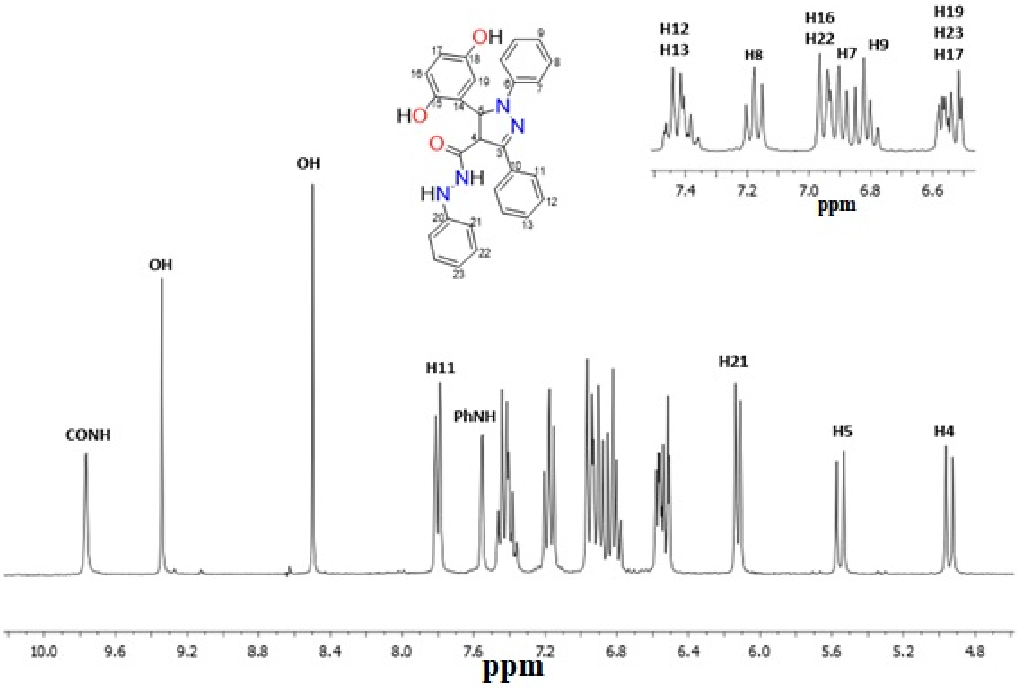

Figure S23. ^{1}H NMR spectrum of compound **2g**, DMSO-d6.

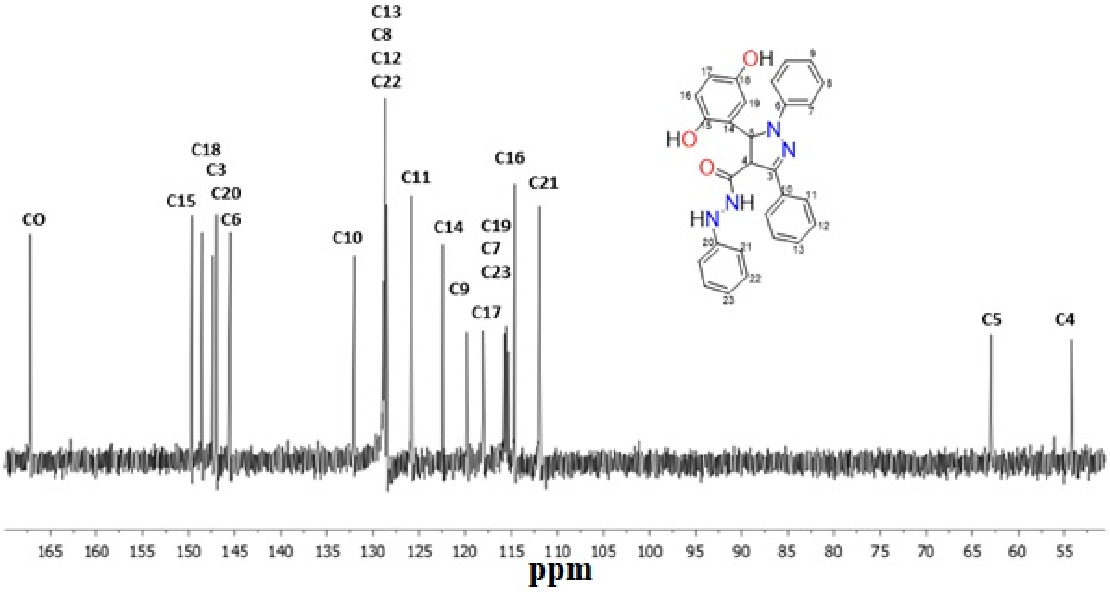

Figure S24. ^{13}C NMR spectrum of the compound **2g**, DMSO-d6.

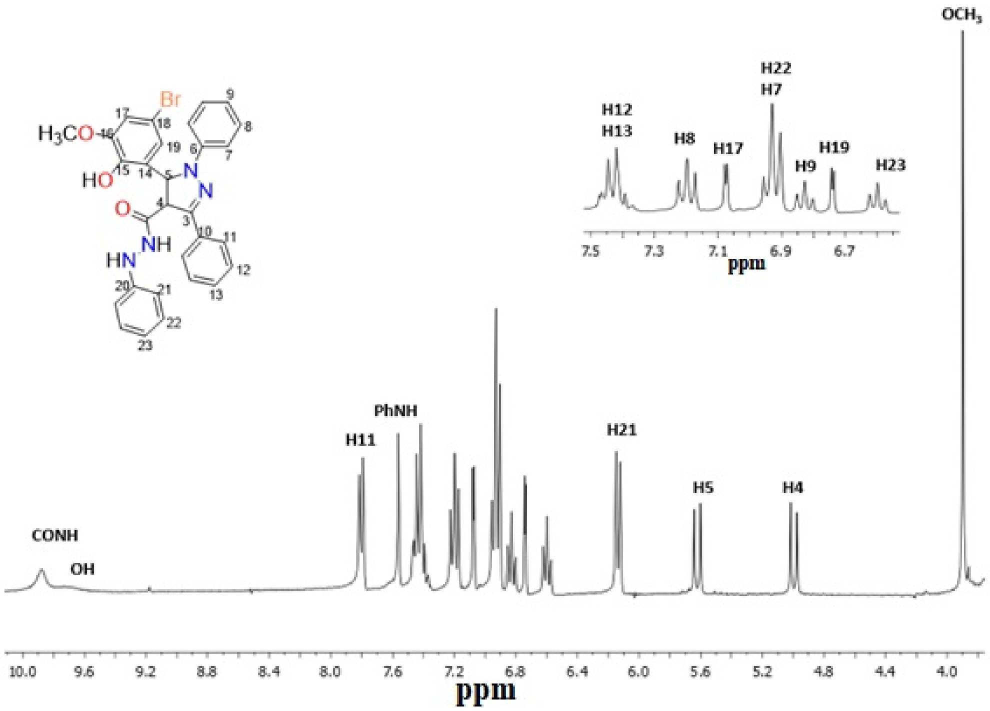

Figure S25. ^{1}H NMR spectrum of compound **2h**, DMSO-d6.

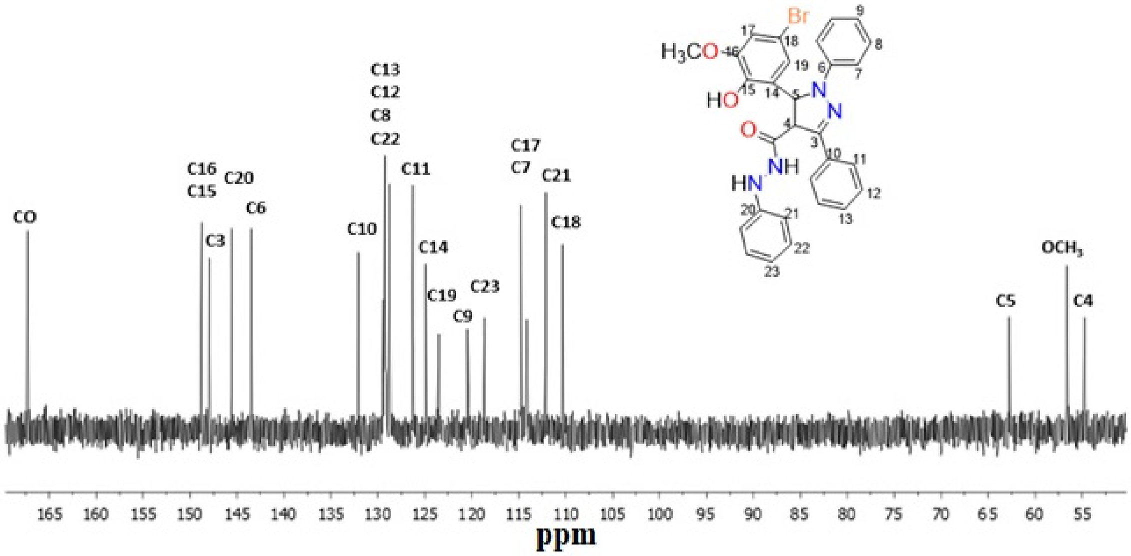

Figure S26. ^{13}C NMR spectrum of the compound **2h**, DMSO-d6.

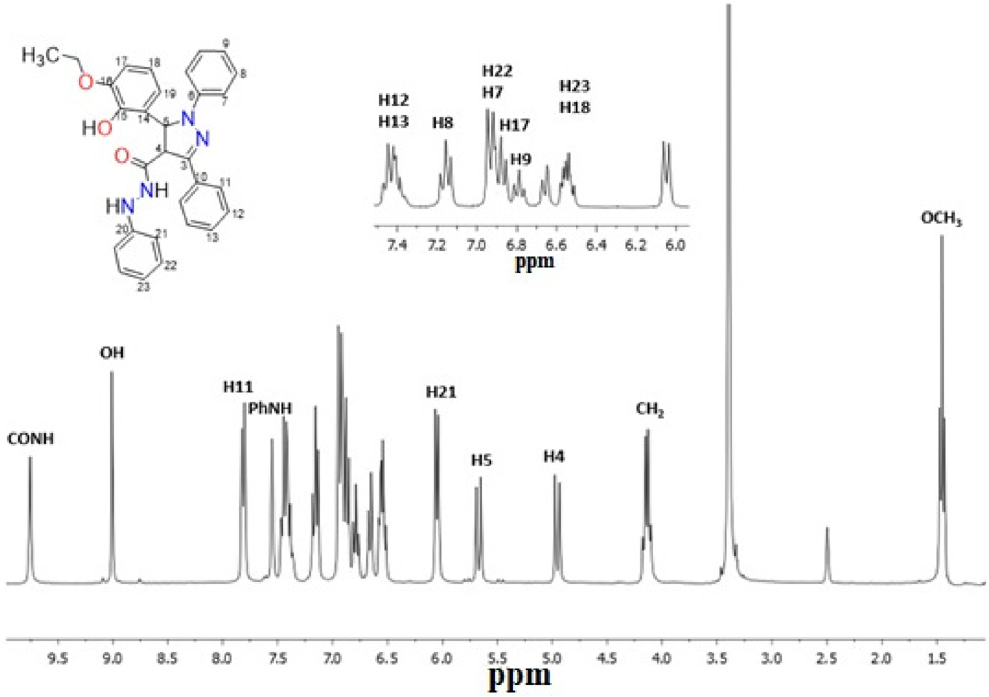

Figure S27. ^{1}H NMR spectrum of compound **2i**, DMSO-d6.

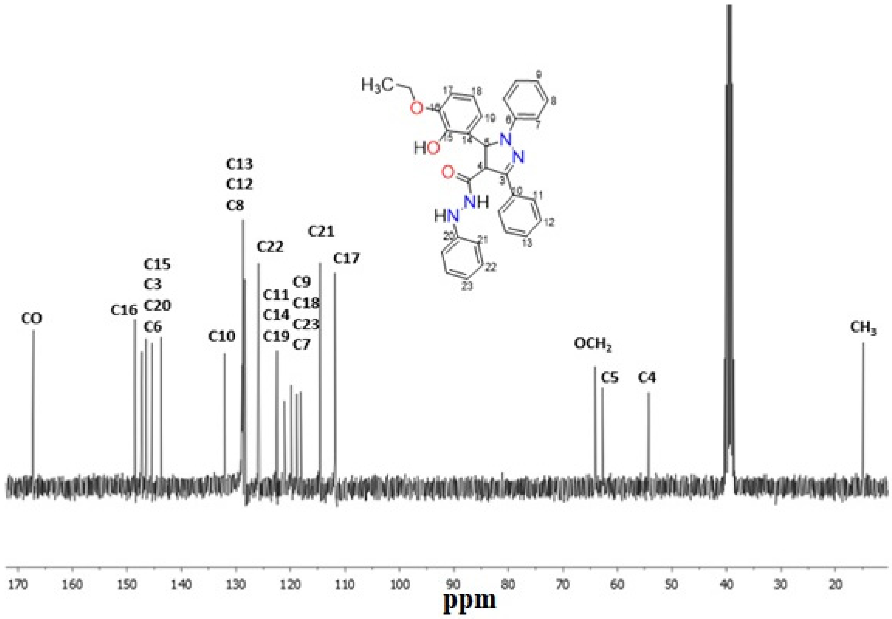

Figure S28. ^{13}C NMR spectrum of the compound **2i**, DMSO-d6.

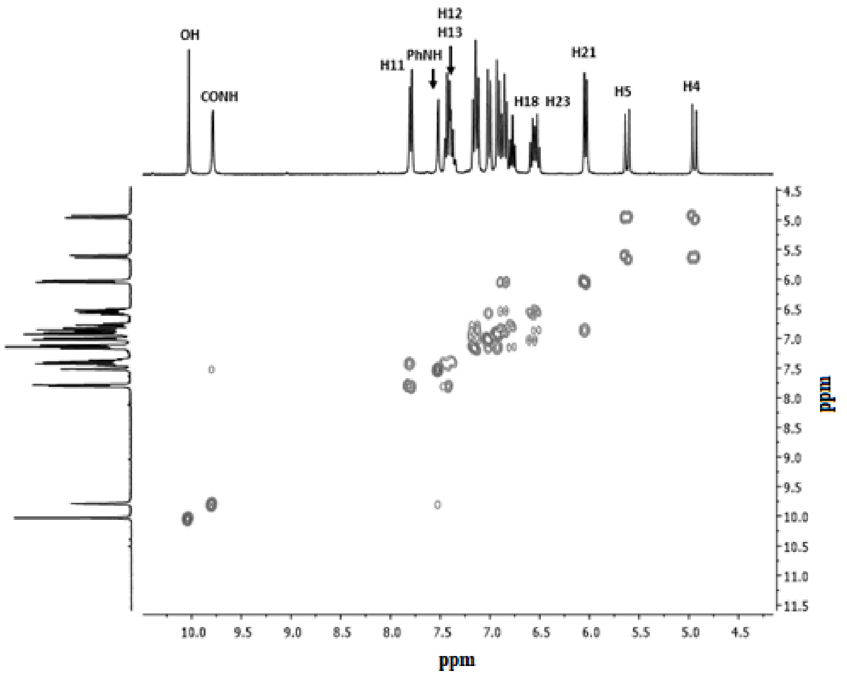

Figure S29. COSY spectrum of compound 5-(2-hydroxyphenyl)-N',1,3-triphenyl-4,5-dihydro-1*H*-pyrazole-4-carbohydrazide (**2a**).

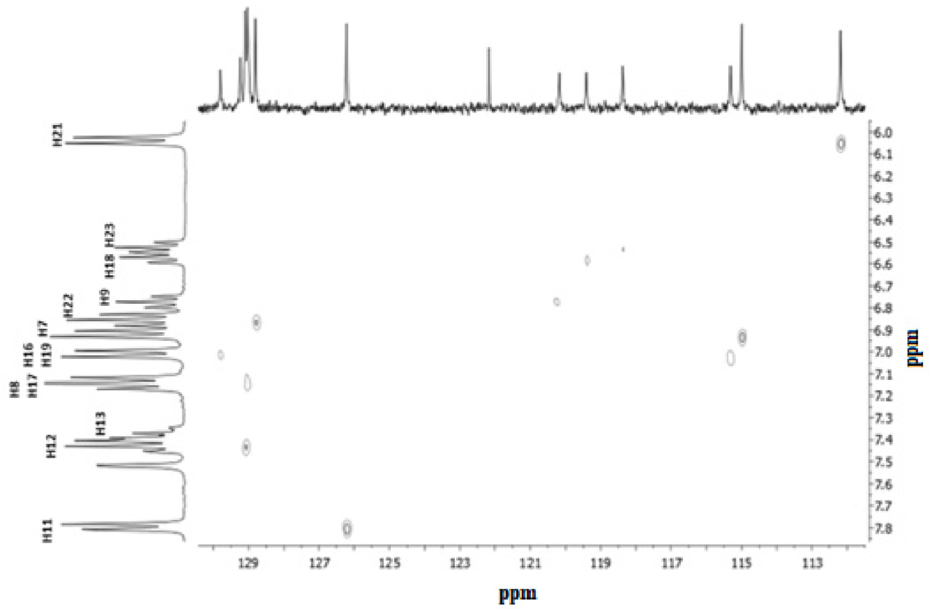

Figure S30. HETCOR spectrum of compound 5-(2-hydroxyphenyl)-N',1,3-triphenyl-4,5-dihydro-1*H*-pyrazole-4-carbohydrazide (**2a**).

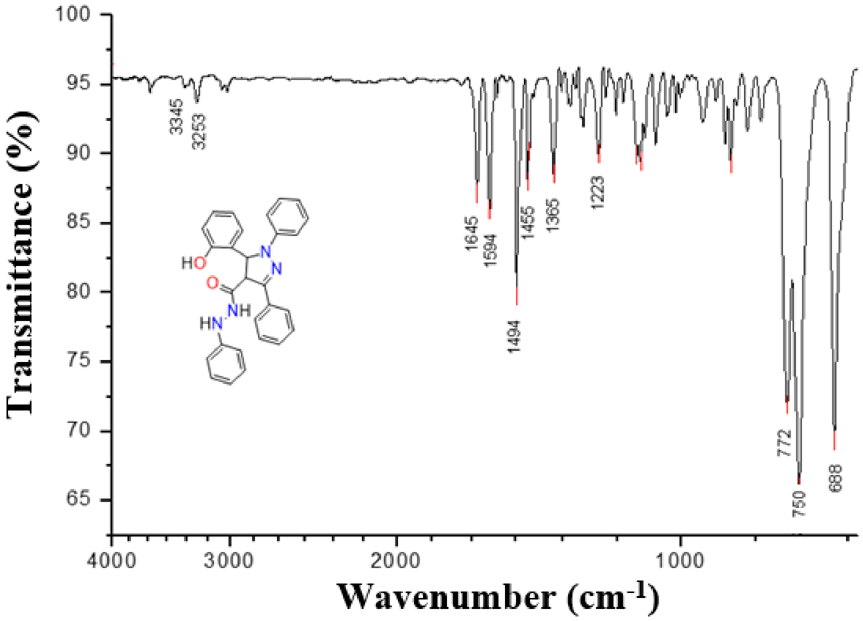

Figure S31. IR spectrum (cm-1) of compound **2a**.

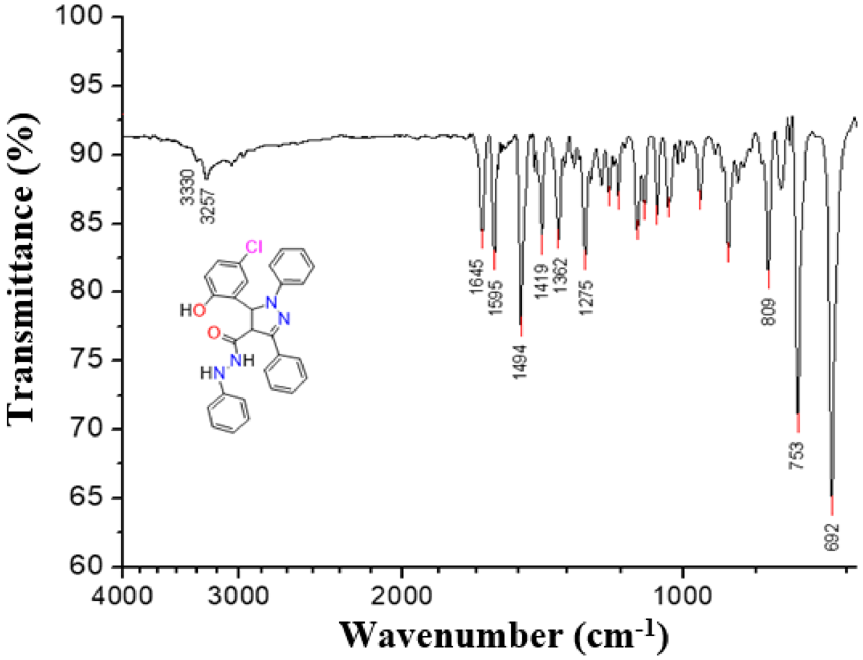

Figure S32. IR spectrum (cm-1) of compound **2b**.

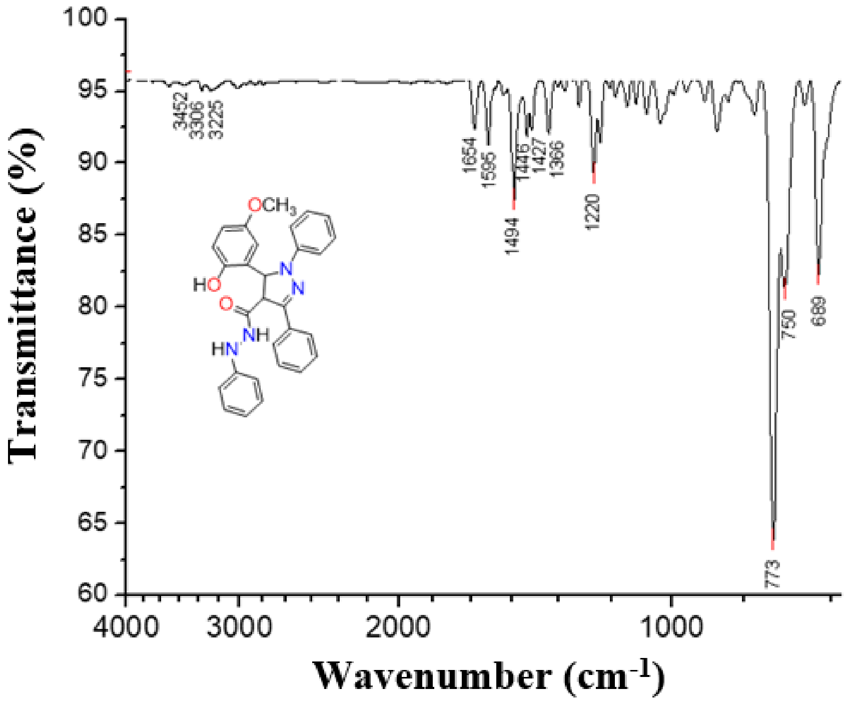

Figure S33. IR spectrum (cm-1) of compound **2c**.

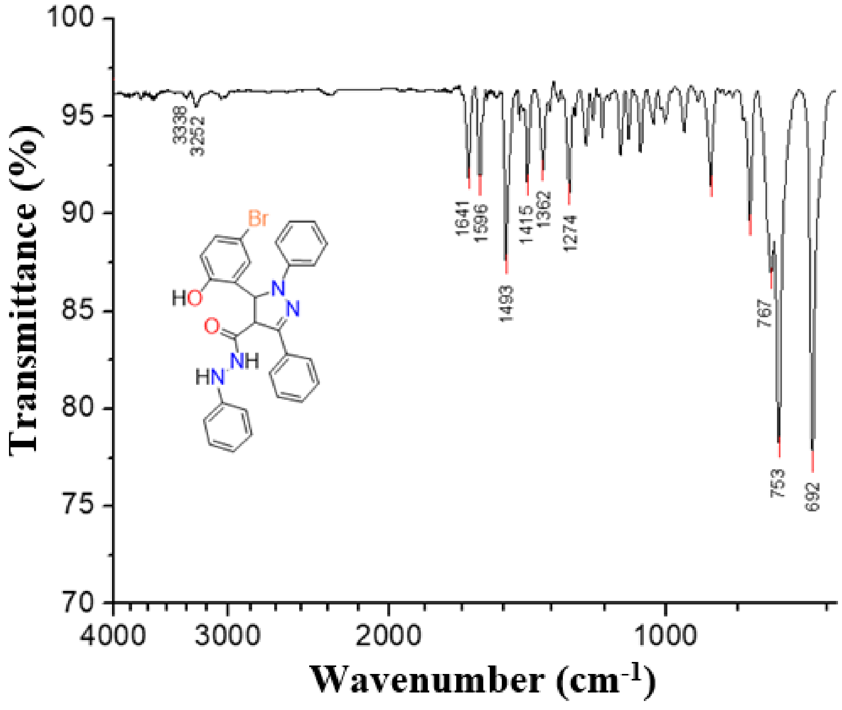

Figure S34. IR spectrum (cm-1) of compound **2d**.

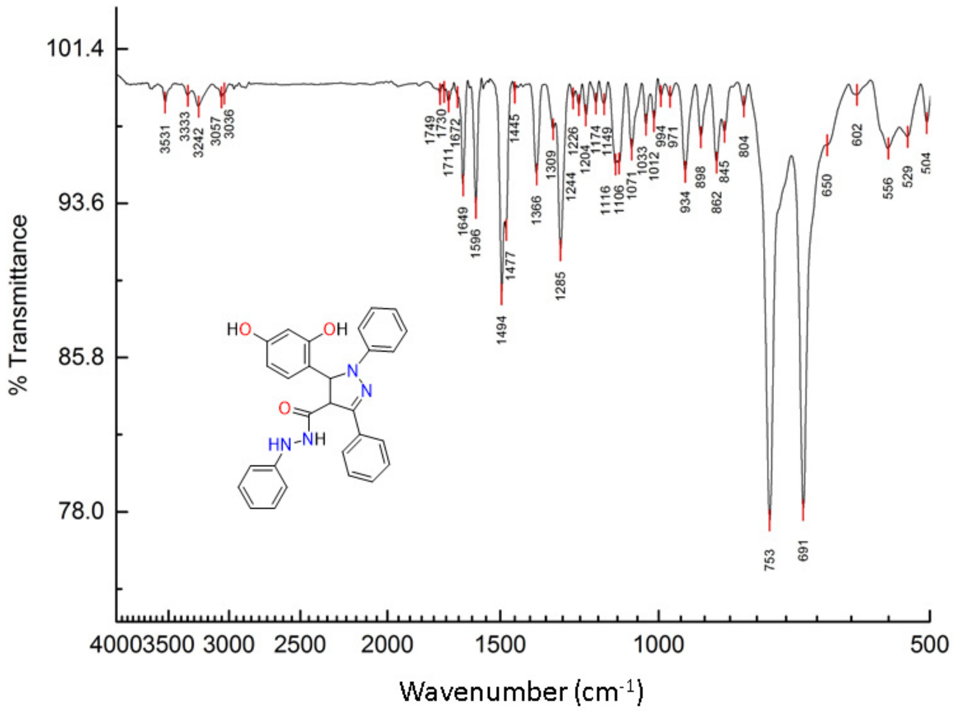

Figure S35. IR spectrum (cm-1) of compound **2e**.

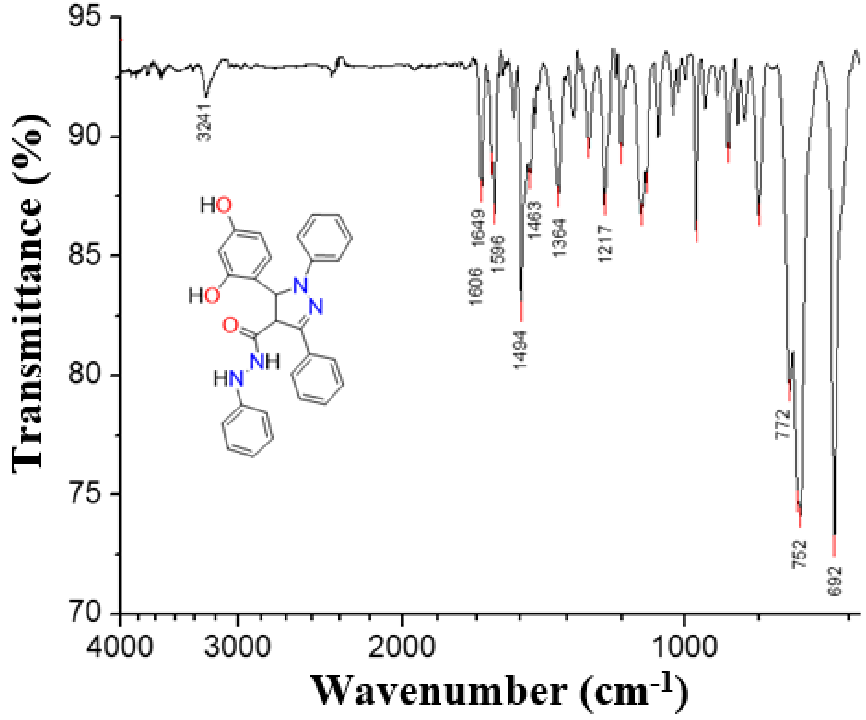

Figure S36. IR spectrum (cm-1) of compound **2f**.

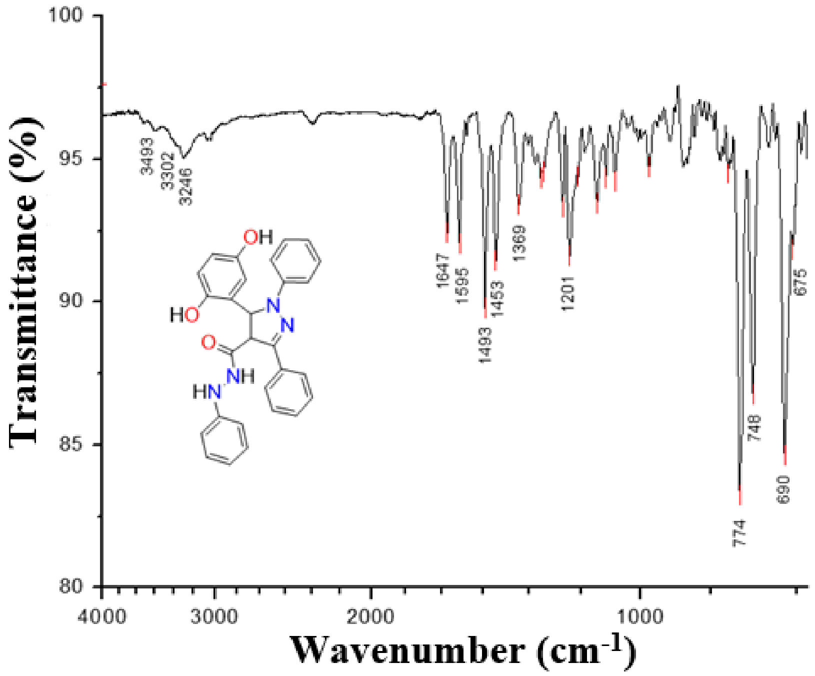

Figure S37. IR spectrum (cm-1) of compound **2g**.

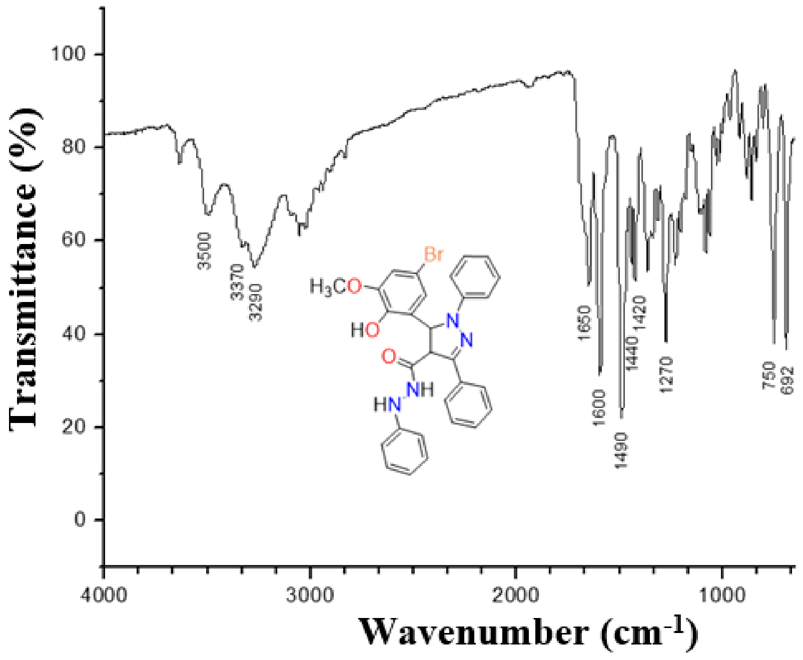

Figure S38. IR spectrum (cm-1) of compound **2h**.

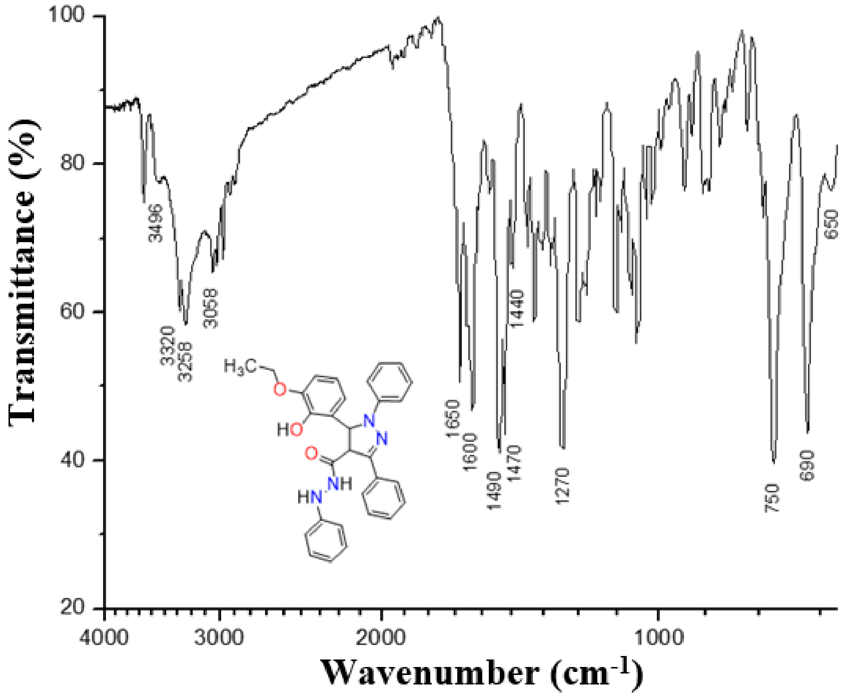

Figure S39. IR spectrum (cm-1) of compound **2i**.

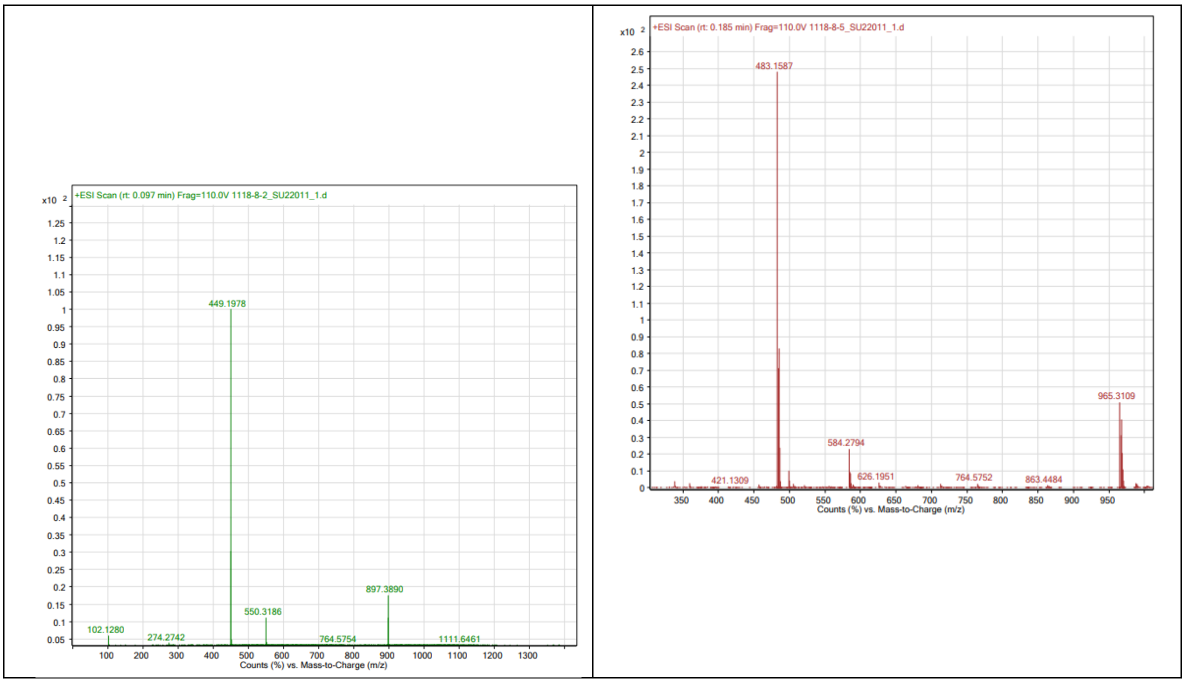

Figure S40. Mass spectra of compounds **2a** (left) and **2b** (right).

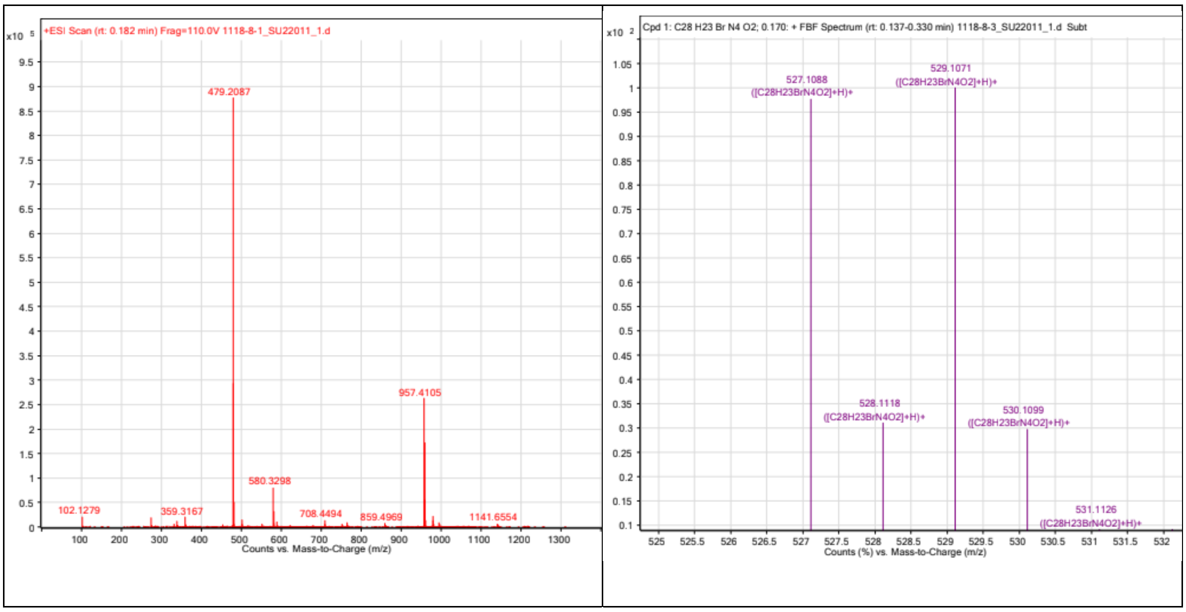

Figure S41. Mass spectra of compounds **2c** (left) and **2d** (right).

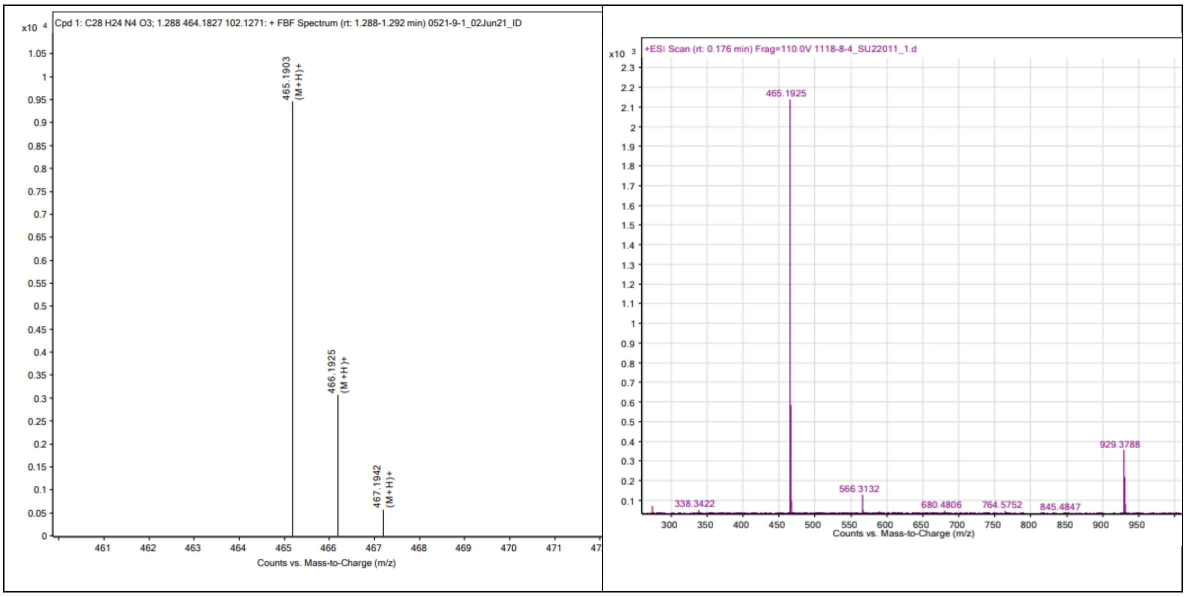

Figure S42. Mass spectra of compounds **2e** (left) and **2f** (right).

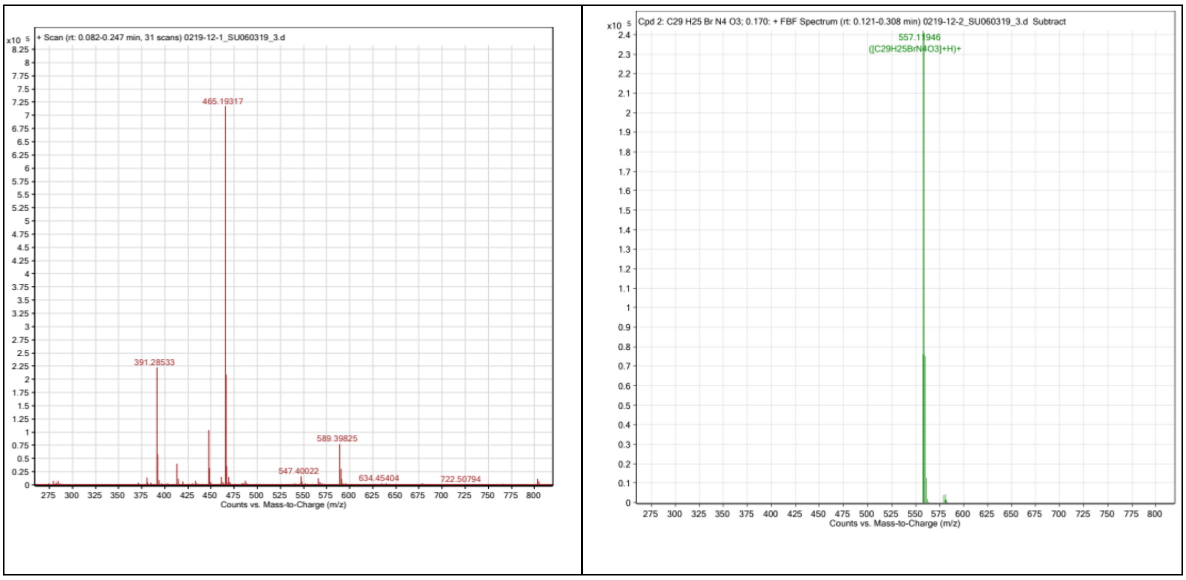

Figure S43. Mass spectra of compounds **2g** (left) and **2h** (right).

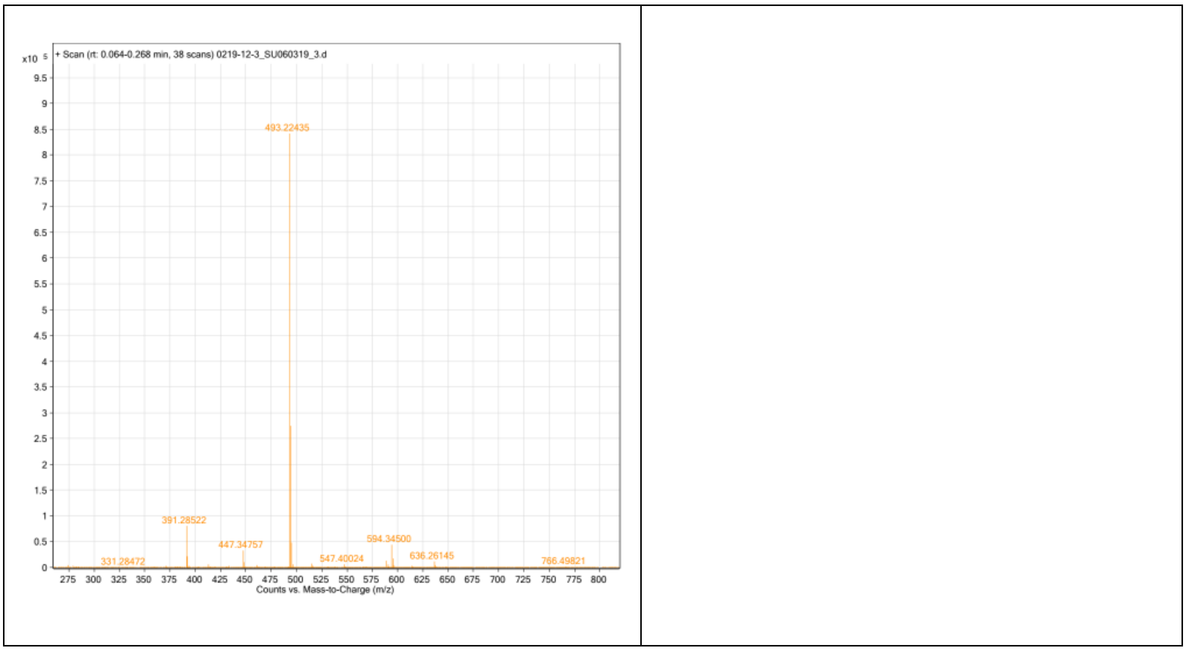

Figure S44. Mass spectra of compound **2i**.

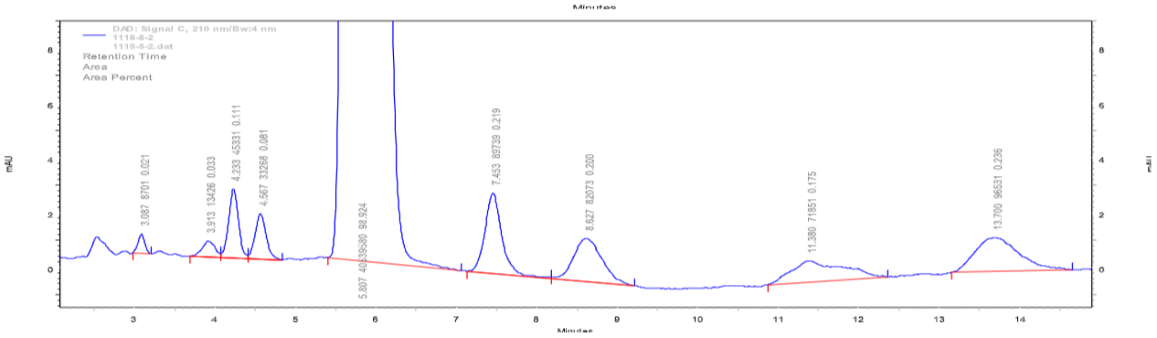

Figure S45. Chromatogram-Purity (98.924%) of **2a**.

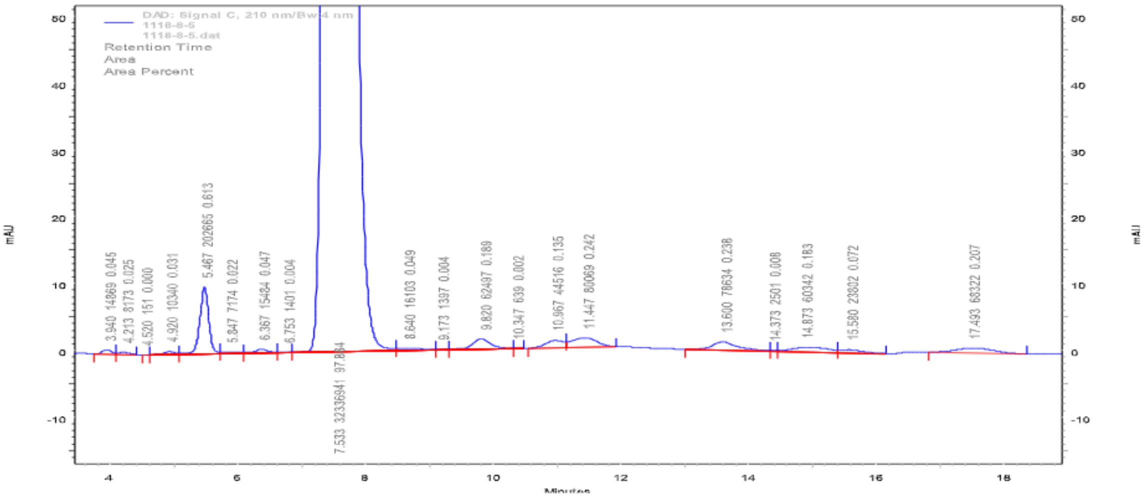

Figure S46. Chromatogram-Purity (97.884%) of **2b**.

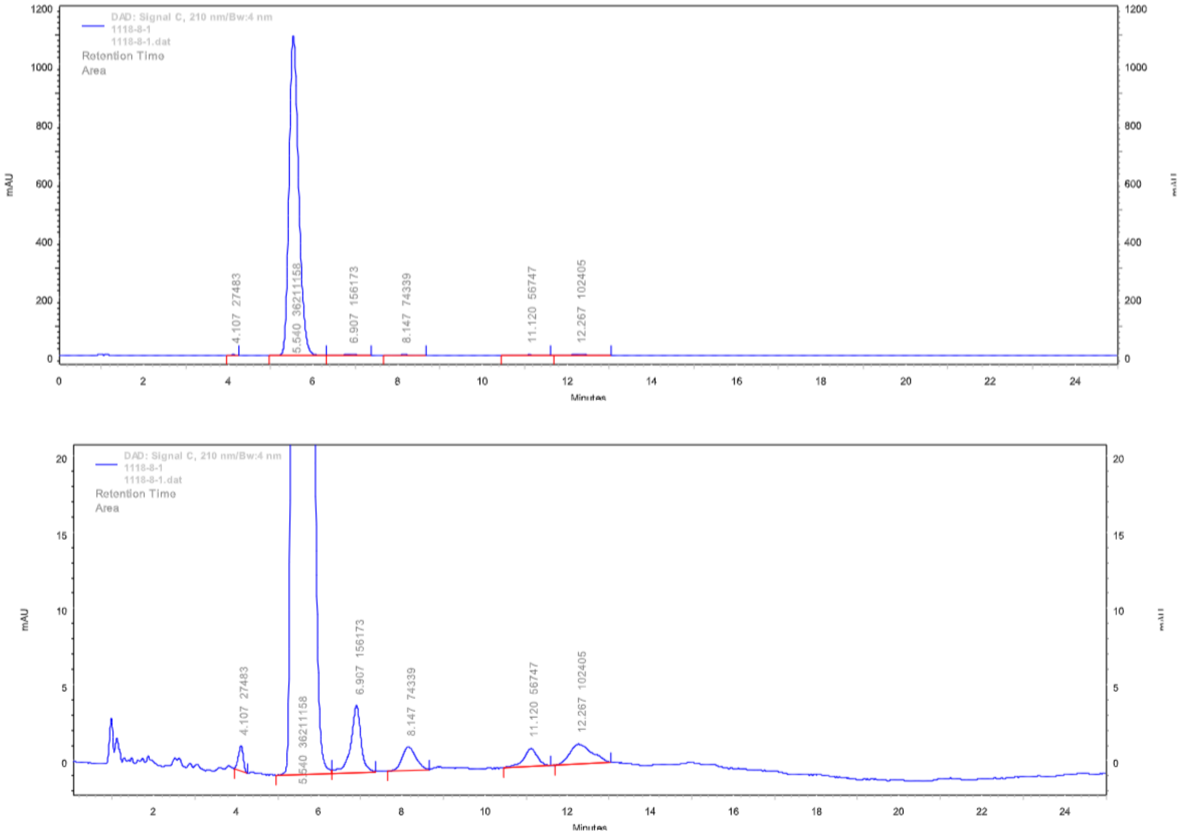

Figure S47. Chromatogram-Purity (98.86%) of **2c**.

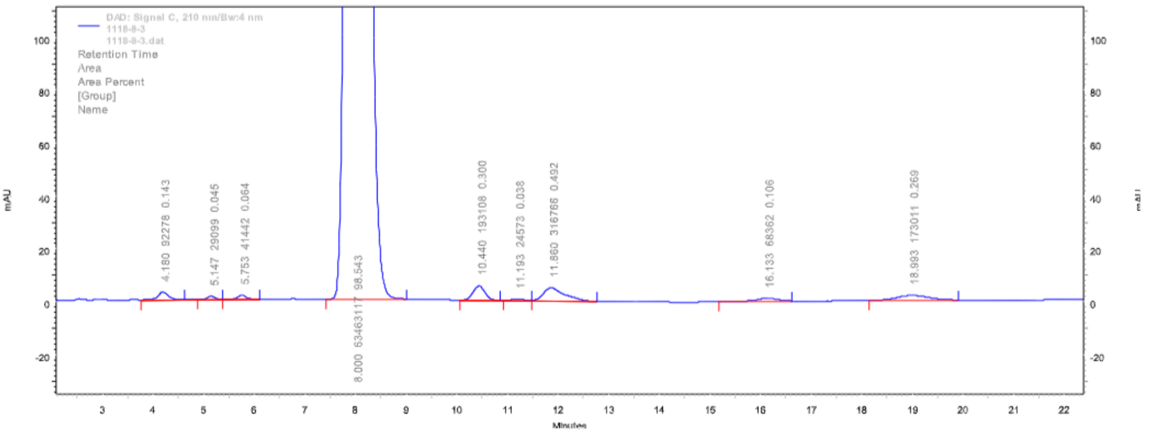

Figure S48. Chromatogram-Purity (98.54%) of **2d**.

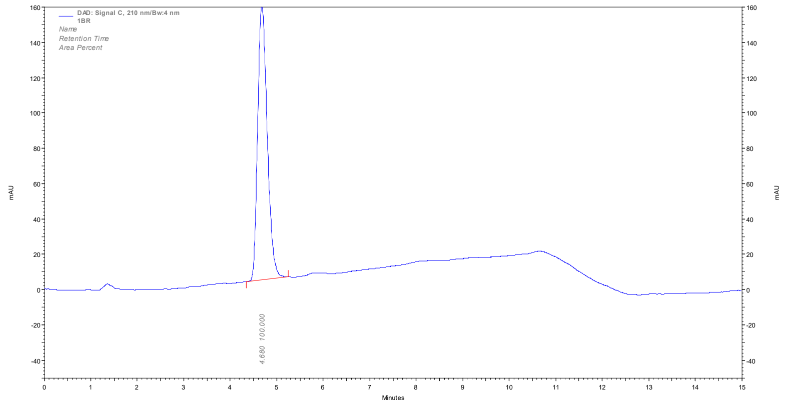

DAD: Signal C,
210 nm/Bw:4 nm
Results

Retention Time	Area	Area %	Height	Height %
4.680	4665572	100.00	326641	100.00
Totals	4665572	100.00	326641	100.00

Figure S49. Chromatogram-Purity (100%) of **2e**.

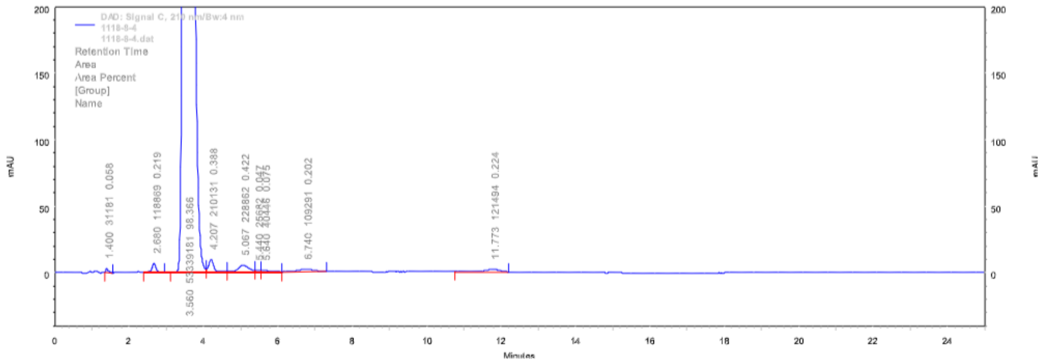

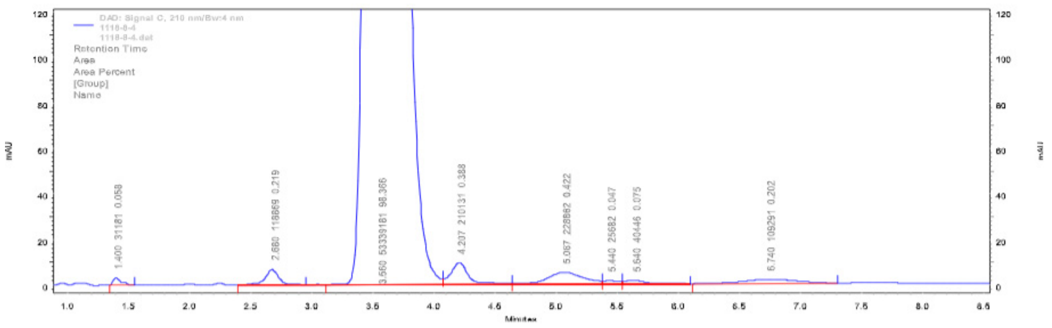

Figure S50. Chromatogram-Purity (98.36%) of **2f**.

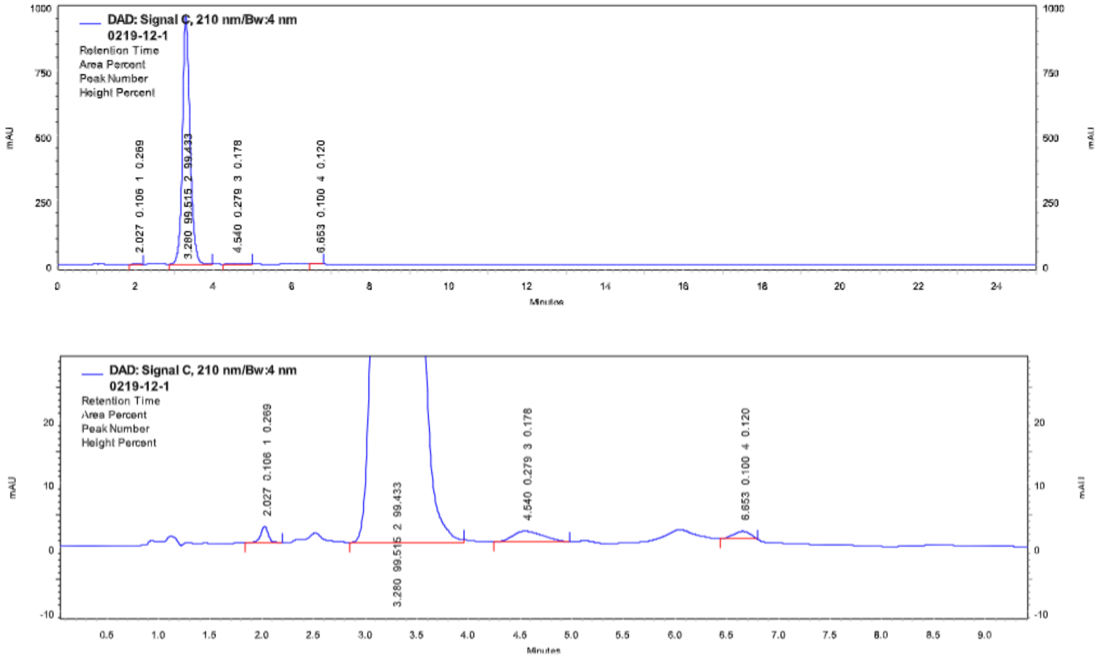

Figure S51. Chromatogram-Purity (99.43%) of **2g**.

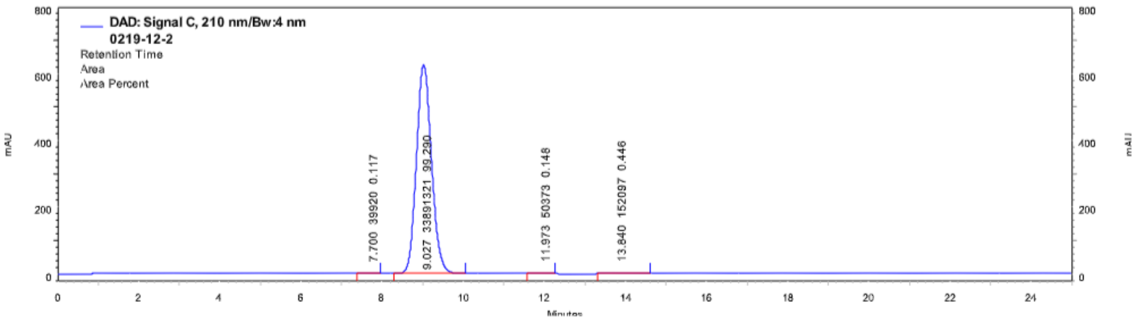

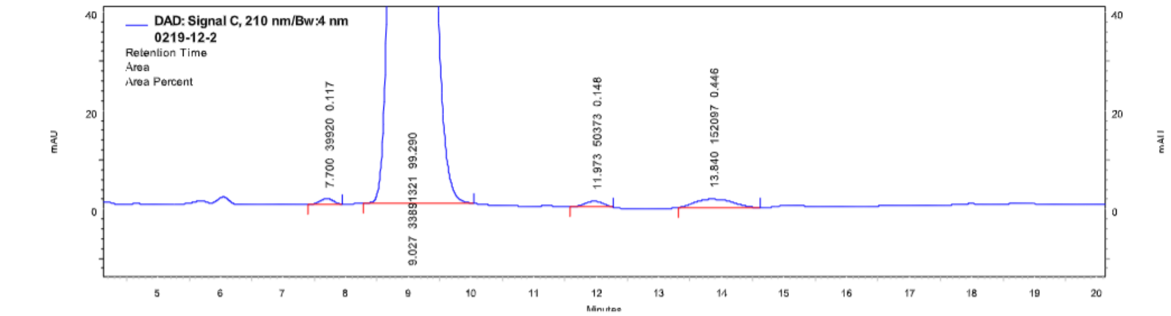

Figure S52. Chromatogram-Purity (99.29%) of **2h**.

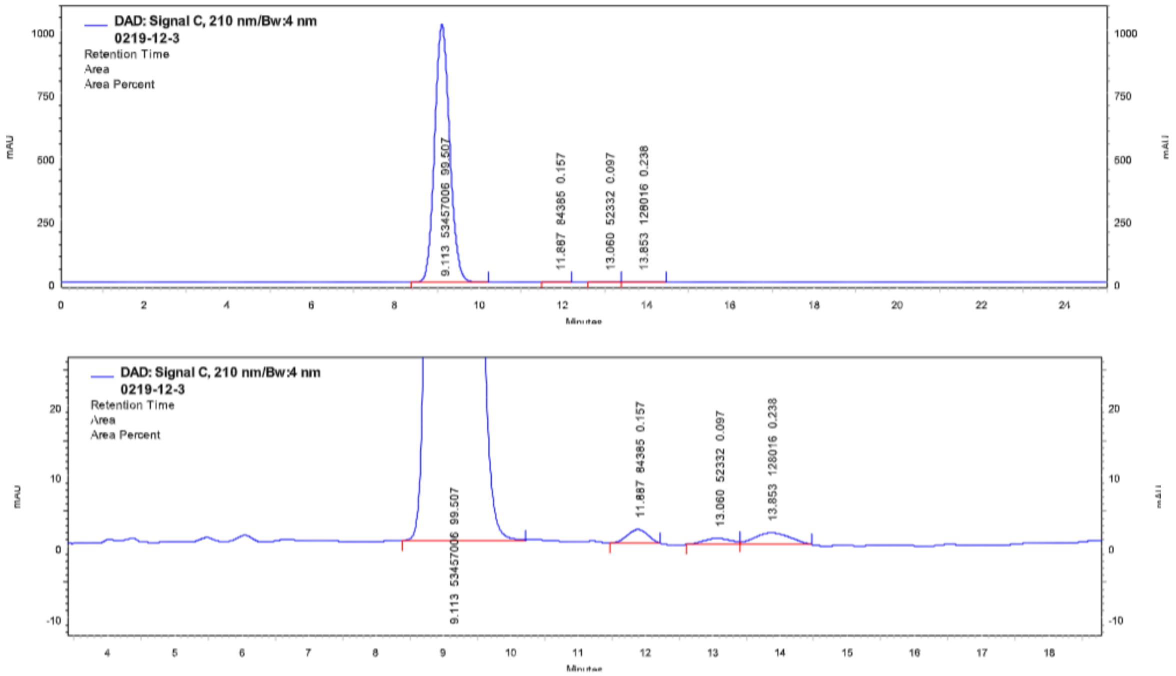

Figure S53. Chromatogram-Purity (99.50%) of **2i**.

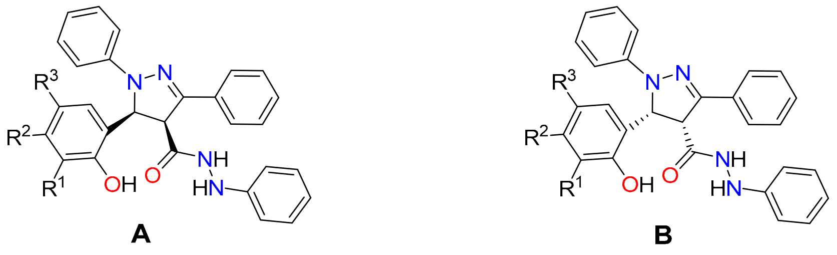

Figure S54. Enantiomers (4S, 5S) A and (4R, 5R) B of modelled 4,5-dihydropyrazole derivatives.

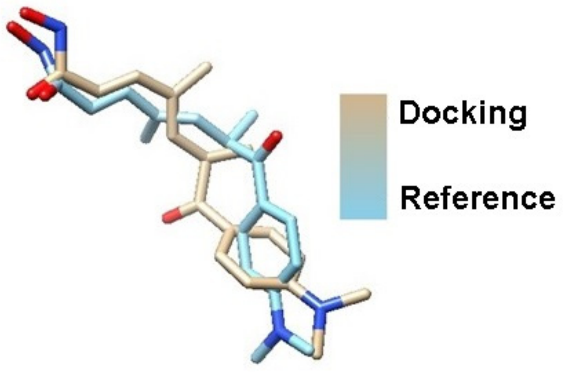

Figure S55. Overlay of TSA in the DD2-HDAC6 domain with an r.m.s.d. value of 2.05.

Table S1. Bond lengths (Å), Bond angles and torsion angles (°).

Atoms	Bond Length (Å)		Atoms	Bond Length (Å)		Atoms	Bond Length (Å)
			O15-C15	1.361(4)		N1-N2	1.395(3)
O24-C24	1.222(3)		N1-C5	1.485(4)		N1-C6	1.418(4)
N2-C3	1.279(4)		N3-N4	1.399(5)		N3-C24	1.338(4)
N4-C20	1.396(4)		C3-C4	1.526(4)		C3>C10	1.469(4)
C4-C5	1.561(4)		C4-C24	1.521(4)		C5-C14	1.519(4)
C6-C7	1.388(4)		C6-C7A	1.391(4)		C7-C8	1.383(5)
C7A-C8A	1.383(5)		C8-C9	1.382(5)		C8A-C9	1.369(5)
C10-C11	1.395(4)		C10-C11A	1.390(4)		C11-C12	1.379(5)
C11A-C12A	1.373(5)		C12-C13	1.372(5)		C12A-C13	1.373(5)
C14-C15	1.392(4)		C14-C19	1.390(4)		C15-C16	1.393(5)
C16-C17	1.372(5)		C17-C18	1.373(5)		C18-C19	1.379(5)
C20-C21	1.396(5)		C20-C21A	1.376(5)		C21-C22	1.372(6)
C21A-C22A	1.382(6)		C22-C23	1.361(6)		C22A-C23	1.378(6)
O15>H15	0.77(4)		N3>H3	0.84(4)		N4>H4	0.76(4)
S25-O25	1.496(3)		S25-C26	1.728(6)		S25-C27	1.734(4)
Atoms	Bond angle (°)		Atoms	Bond angle (°)		Atoms	Bond angle (°)

N2-N1-C5	110.7(2)		N2-N1-C6	114.5(2)		C5-N1-C6	121.8(2)
N1-N2-C3	110.1(2)		N4-N3-C24	119.4(3)		N3-N4-C20	118.3(3)
N2-C3-C4	114.0(2)		N2-C3-C10	120.4(2)		C4-C3-C10	125.6(2)
C3-C4-C5	100.6(2)		C3-C4-C24	108.5(2)		C5-C4-C24	115.7(2)
N1-C5-C4	102.7(2)		C15-O15-H15	110(3)		N4-N3-H3	122(2)
C24-N3-H3	117.9(19)		N3-N4-H4	114(3)		C20-N4-H4	116(3)
N1-C5-C14	113.3(2)		C4-C5-C14	117.4(2)		O24-C24-N3	123.0(3)
N3-C24-C4	114.8(3)		O24-C24-C4	122.1(2)		N4-C20-C21	123.8(3)
Atoms	Torsion angle (°)		Atoms	Torsion angle (°)		Atoms	Torsion angle (°)
C4C3N2N1	-0.7(3)		C3C4C5N1	14.20(19)		N2N1C5C4	-12.7(2)
C5N1N2C3	-8.8(3)		N2N1C6C7	19.3(4)		N1C5C14C19	-7.4(4)
C5C4C24N3	118.0(3)		N2C3C10C11	22.7(4)		N3N4C20C21	-168.3(3)
H15O15C15C16	-6(3)		H3N3N4H4	67(4)		H3N3C24O24	174(2)
S25-O25	1.496(3)		S25-C26	1.728(6)		S25-C27	1.734(4)

Table S2. Hydrogen bonding geometry parameters of **2a**.

D—H···A		D—H (Å)	H···A (Å)	D···A (Å)	D—H···A (°)
N3—H3···O24i	x, −y + ½, z + ½	0.83 (3)	2.43 (3)	3.198 (4)	154 (3)
N4—H4···O25ii	−x+1, y − ½, −z+ ½	0.76 (4)	2.48 (4)	3.209 (5)	162 (4)
O15—H15···O25iii	−x+1, −y+1, −z+1	0.78 (3)	1.94 (4)	2.702 (3)	169 (4)
C4—H4A···O24i	x, −y + ½, z + ½	0.98	2.57	3.462 (3)	151

Table S3. Free binding energy $\Delta G_b°$ (kcal/moL) and K_d (μM) values obtained by docking the DD2-HDAC6 domain with 4,5-dihydropyrazole derivatives **2a-i**. **A** is the (*4S*, *5S*) and **B** is the (*4R*, *5R*) enantiomer.

Compounds	$-\Delta G_b°$ (kcal/mol)	K_d (μM)
**2a A**	7.80	1.93
**2a B**	7.19	5.39
**2b A**	7.86	1.74
**2b B**	7.84	1.80
**2c A**	7.43	3.57
**2c B**	7.61	2.63
**2d A**	7.82	1.84

**2d B**	7.71	2.24
**2e A**	7.28	4.60
**2e B**	7.24	4.95
**2f A**	6.85	9.47
**2f B**	6.96	7.92
**2g A**	7.48	3.28
**2g B**	7.42	3.64
**2h A**	7.85	1.77
**2h B**	7.37	3.94
**2i A**	7.25	4.82
**2i B**	7.01	7.29
Tubacin	9.97	0.32
TSA	8.59	0.05
SAHA	7.02	7.14

Table S4. Interactions among of the 4,5-dihydropyrazole derivatives compared with Tubacin, TSA and SAHA with the DD2-HDAC6 structure (PDB: 5G0J), obtained by docking studies.

	1	2	3	4	5	6	7	8	9	10	11	12	13	14	15	16	17	18	19	20	21	22
	H	P	D	S	I	Y	H	H	G	F	H	R	T	F	F	M	D	P	L	G	G	Y
Compounds	500	501	567	568	569	570	610	611	619	620	651	673	678	679	680	682	742	748	749	750	751	782
**2a1**		■		■				■	■	■	■			■	■			■	■			
**2a2**	■	■		■				■		■	■			■	■				■			■
**2b1**		■		■				■	■	■	■			■	■				■			
**2b2**			■	■				■	■	■	■			■	■	■			■			■
**2c1**		■		■						■	■		■	■	■				■			
**2c2**				■						■	■	■	■	■	■				■			
**2d1**			■	■				■	■	■	■			■	■				■			■
**2d2**			■	■				■	■	■	■			■	■	■			■			
**2e1**				■				■	■	■	■			■	■				■			■
**2e2**			■	■				■	■	■	■		■	■	■				■			
**2f1**				■				■	■	■	■			■	■			■	■			
**2f2**				■					■	■	■		■	■	■				■			
**2g1**				■						■	■		■	■	■	■			■			
**2g2**			■	■				■	■	■	■			■	■	■			■			
**2h1**				■					■	■	■		■	■	■				■			
**2h2**				■						■	■			■	■				■			
**2i1**	■			■			■	■	■	■	■			■	■				■			■
**2i2**			■	■			■	■	■	■	■			■	■				■			■
Tubacin	■			■	■	■		■	■	■	■	■	■	■	■			■	■	■	■	
TSA				■			■	■		■	■				■			■	■			■

SAHA																						

Table S5. Toxicity profile of the 4,5-dihydropyrazole derivatives compared with tubacin and TSA.

Compounds	Substituent	M	T	I	R
**2a**	H				
**2b**	5-Cl				
**2c**	5-OCH_3				
**2d**	5-Br				
**2e**	3-OH				
**2f**	4-OH				
**2g**	5-OH				
**2h**	3-OCH_3, 5-Br				
**2i**	3-OCH_2CH_3				
Tubacin	NA				
TSA	NA				
SAHA	NA				

Predicted toxico-biological properties in DataWarrior server, were **M** = Mutagenic; **T** = Tumorigenic; **I** = Irritant; **R** = Reproductive effects. **Green** = Safe; **Yellow** = Medium risk; **Red**= High risk. NA= not applicable.

Table S6. Crystal data and details of the structure determination for **2a**.

Crystal Data			Data Collection	
Formula	$C_{28}H_{24}N_4O_2{\cdot}C_2H_6OS$		Temperature (K)	293(2)
Formula Weight	526.64		Radiation [Å]	MoKa 0.71073
Crystal System	Monoclinic		Theta Min-Max [Deg]	3.0, 25.0
Space group	$P2_1/c$ (No. 14)		Dataset	15:-15 ; 28:-28 ; 10:-10
a [Å]	12.6818(12)		Tot., Uniq. Data, R(int)	80540, 4731, 0.090
b [Å]	23.981(2)		Observed Data [I > 2.0 sigma(I)]	3069
c [Å]	9.0955(9)		Refinement	
α, β, γ [°]	90, 103.661(9), 90		Nref, Npar	4731, 357
V [Å^3]	2687.9(4)		R, wR2, S	0.057, 0.153, 0.00
Z	4		Max. and Av. Shift/Error	0.00, 0.00

D(calc) [g/cm^3]	1.301		Min. and Max. Resd. Dens. [e/Å^3]	-0.529, 0.181
Mu(MoKa) [mm^{-1}]	0.159		GOOF	1.031
F(000)	1112			
Crystal Size [mm]	0.30 x 0.25 x 0.20			
